# Self-Assembled Ordered Nanostructure of Zwitterionic Co-Solutes Induces Localized High-Concentration Electrolytes for Ultrastable and Efficient Zinc Metal Anodes

**DOI:** 10.1007/s40820-025-02040-4

**Published:** 2026-01-04

**Authors:** Shengyang Huang, Zuyang Hu, Xin Wang Mo, Yeonju Park, Jun Su Kim, Gun Jang, Dong Hyun Min, Hao Fu, Peixun Xiong, Zhipeng Wen, Young Mee Jung, Jaeyun Kim, Hyunjoo Lee, Chihyun Hwang, Youngkwon Kim, Cheng Chao Li, Qingyun Dou, Ho Seok Park

**Affiliations:** 1 School of Chemical Engineering, Sungkyunkwan University (SKKU), 2066 Seobu-Ro, Jangan-Gu, Suwon, 16419 Republic of Korea; 2 Guangdong Provincial Key Laboratory of Plant Resources Biorefinery, School of Chemical Engineering and Light Industry, Guangdong University of Technology, Guangzhou, 510006 People’s Republic of China; 3 Department of Chemistry, Institute for Molecular Science and Fusion Technology, Kangwon National University, Chuncheon, 24341 Republic of Korea; 4 Kangwon Radiation Convergence Research Support Center, Kangwon National University, Chuncheon, 24341 Republic of Korea; 5 Clean Energy Center, Korea Institute of Science and Technology (KIST), 5, Hwarang-Ro 14-Gil, Seongbuk-Gu, Seoul, 02792 Republic of Korea; 6 Advanced Batteries Research Center, Korea Electronics Technology Institute (KETI), 25, Saenari-Ro, Seongnam, 13509 Republic of Korea; 7 Department of Materials Science and Engineering, Sun Yat-Sen University, Guangzhou, 510275 People’s Republic of China; 8 SKKU Institute of Energy Science and Technology (SIEST), Sungkyunkwan University, 2066, Seoburo, Jangan-Gu, Suwon, 440-746 Republic of Korea

**Keywords:** Localized high-concentration electrolytes, Self-assembled, Multifunctional additives, Zwitterions, Zn metals

## Abstract

Localized high-concentration electrolytes (LHCEs) are considered as promising electrolyte candidates to resolve technical issues of metal batteries owing to their unique interfacial properties and solvation structures. Herein, we propose a self-assembly chemical strategy into the LCHEs induced by ordered nanostructure of zwitterionic co-solutes for highly efficient and ultrastable zinc (Zn) metal batteries. Through the systematic screening of six zwitterionic compounds, 3-(decyldimethylammonio)propanesulfonate salt (C_10_) with the decyl chain and zwitterions was determined as an optimum to construct quasi-spherical aggregates with a periodic length of 3.77 nm, as confirmed by comprehensive synchronous small-angle X-ray scattering, Guinier, pair distance distribution function, Porod, and other spectroscopic characterizations and molecular dynamic simulation. In particularly, this self-assembled structure in electrolyte environments was attributed to increasing the proportion of both contact and aggregated ion pairs for the formation of LHCEs as well as to providing fast and selective Zn^2+^ conducting channels and uniform solid electrolyte interfaces for facilitated charge transfer kinetics. Moreover, the preferential adsorption of the self-assembled C_10_ on the Zn(002) surface modulated the electrical double layer to suppress hydrogen evolution and corrosion reactions. Consequently, the Zn||Zn symmetric cells in Zn(OTf)_2_/C_10_ electrolytes showed long-term plating/stripping behaviors over 2800 h at 1 mA cm^−2^ and 1 mAh cm^−2^ as well as over 1200 h even at 5 mA cm^−2^ and 5 mAh cm^−2^ with a very high depth of discharge of 42.7%. Furthermore, the Zn||VO_2_/CNT full cells in Zn(OTf)_2_/C_10_ electrolytes delivered a record-high capacity of 8.10 mAh cm^−2^ at an ultrahigh cathode mass loading of 50 mg cm^−2^ after 150 cycles.

**Supplementary Information:**

The online version contains supplementary material available at 10.1007/s40820-025-02040-4.

## Highlights

- Self-assembled zwitterion (C_10_) induces localized high-concentration electrolytes, regulating Zn^2+^ solvation, guiding selective ion transport, and enabling uniform solid electrolyte interface formation.
- A comprehensive set of advanced analyses (Guinier, PDDF, Porod) combined with spectroscopy and simulations reveals that C_10_ self-assembles into ~3.8 nm quasi-spherical aggregates and bilayer-like interfacial structures.
- C_10_ enables ultrastable cycling (>2800 h) in symmetric cells and record areal capacity (8.1 mAh cm^−2^ at 50 mg cm^−2^) in Zn||VO_2_/CNT full cells, highlighting its practical potential for high-energy Zn batteries.

**Supplementary Information:**

The online version contains supplementary material available at 10.1007/s40820-025-02040-4.

## Graphical abstract

**Figure:**
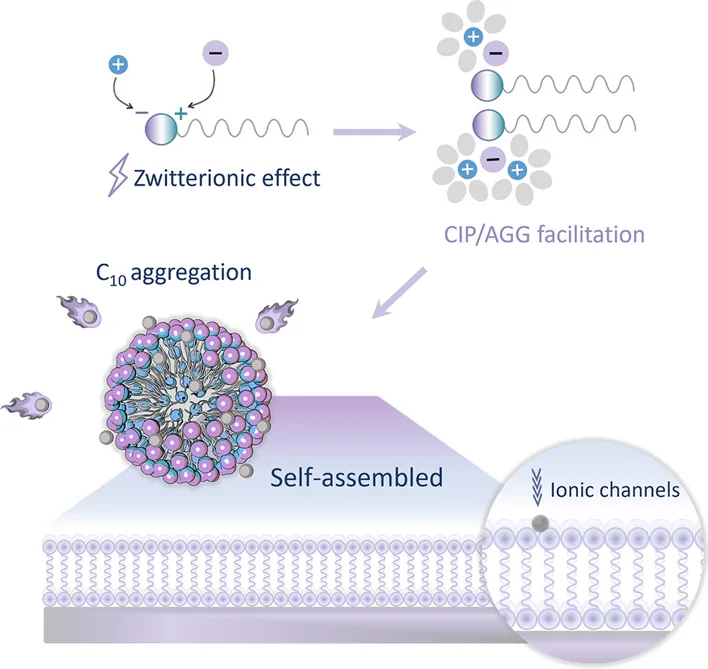

## Introduction

In recent years, the ever-increasing demand for safe and cost-effective energy storage systems has motivated the development of advanced rechargeable batteries [1–3]. Among various battery technologies, aqueous zinc metal batteries (ZMBs) are considered as promising alternatives to traditional lithium-ion batteries (LIBs) owing to their low cost, inherent safety, environmental friendliness, and high theoretical capacity of zinc (Zn) anode (820 mAh g^−1^ or 5855 mAh cm^−3^) [4]. However, the practical application of ZMBs has been hindered by their relatively low energy density and limited cycling stability, primarily resulting from severe side reactions and the uncontrolled growth of Zn dendrites [5, 6]. To address these challenges, electrolyte and interfacial engineering, such as precise electrolyte composition design, artificial solid electrolyte interface (SEI) development, and solid-state electrolyte innovations [7, 8], have been investigated so far. In particular, electrolyte additives are regarded as the practical and effective solution owing to their simplicity and efficiency. Examples vary from inorganic anions or cations to adsorptive organic molecules, supramolecular compounds, and polymers [9, 10]. However, the performance improvements by these additives still remain limited. For example, inorganic anions may participate in the controlled solvated structure or SEI formation, but offer minimal guidance for Zn^2+^ deposition [11]. Inorganic cations mainly address the "tip effect" by limiting dendrite growth through competitive reactions, yet they have little influence on the solvating regulation [12]. Moreover, supramolecular compounds and polymer additives are limited by complex synthesis processes, significant increases in internal resistance, and poor performance under high current densities [13]. Common adsorptive organic molecules also face challenges such as incomplete coverage of the Zn anode, inhibited Zn^2+^ transport kinetics, and increased polarization [14]. When the concentration of additives increased to improve coverage, excessive viscosity or poor solubility often occurred due to the "like dissolves like" principle, which makes it difficult to further regulate the electrolyte environment and interfacial regions [15]. Furthermore, it is very challenging to fully understand the nanoscale structural changes in the electrolyte environment induced by these additives due to the lacking of in-depth analysis on the solution environment [16–18].

The design of new co-solute additives is critical for forming stable solution environments, regulating solvation structures, modifying electric double layer (EDL) for the suppression of side reactions, uniform electric and ionic field distributions, and reversible Zn deposition [19, 20]. The introduction of self-assembled or structured additives is expected to effectively construct ion channels on the EDL and provide favorable pathways for Zn^2+^ ion transfer. This interphacial modification also reduces a direct contact of water with the electrode surface to suppress hydrogen evolution reaction (HER) and corrosion. Examples include the inorganic ZnV_3_O_8_, the polymer methoxy polyethylene glycol-phosphate (mPEG-P), and the biomolecule bovine serum albumin (BSA) [21–23]. Although the concept of self-assembly has been demonstrated in these works, in-depth characterization and detailed analyses on the ordered structure are still lacking. In order to address this limitation, more comprehensive analytical techniques are needed to provide the fundamental foundation for the self-assembly into the ordered structure [24]. In this work, we first applied these analytical techniques, including Guinier, pair distance distribution function (PDDF), Porod analysis, and theoretical calculations, for the deep understanding about the local environment of nanostructures.

It has been known that high-concentration electrolytes (HCEs) have several advantages over dilute electrolytes, in terms of the formation of stable SEI layers, suppression of dendrite growth, and expansion of the electrochemical stability window [25]. The reduced solvent activity of HCEs is beneficial for mitigating the notorious HER in aqueous batteries, as demonstrated by water-in-salt electrolyte with 21 molality of LiTFSI [26]. However, HCEs suffer from inherent drawbacks such as extremely high viscosity and the impractical use of large quantities of salts. To resolve these issues, localized high-concentration electrolytes (LHCEs) were developed as an alternative candidate to HCEs. LHCEs retain the key feature of a locally anion-rich coordination environment while significantly lowering the overall salt concentration, which leads to improve ionic conductivity, facilitate SEI formation, and alleviate issues related to viscosity and processing [27]. Despite their advantages, traditional LHCEs that rely on inert diluents may suffer from limited interfacial tunability and potential stability issues [28].

**Fig. 1:**
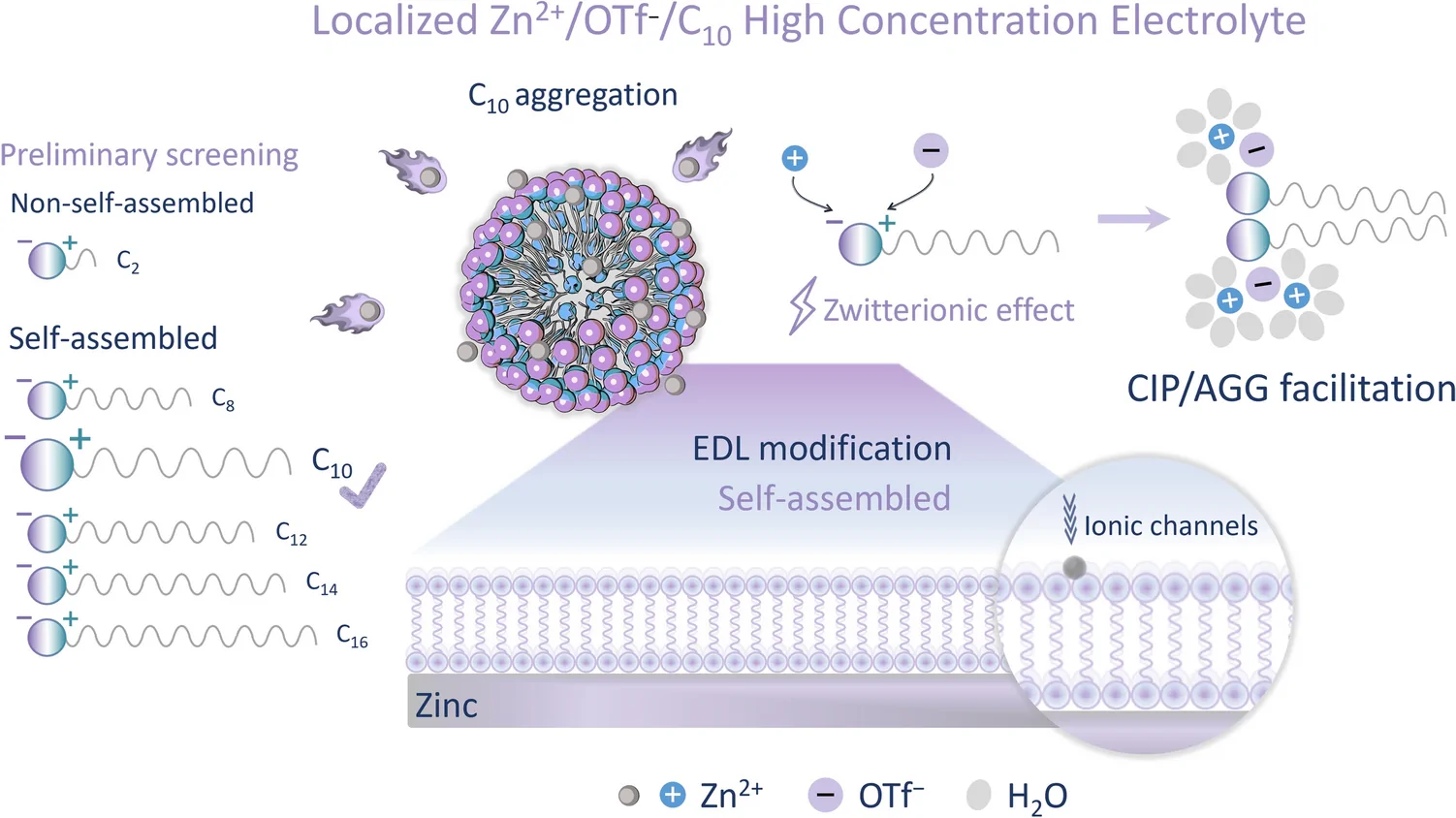
Schematic illustration of the roles of the C_10_ co-solute in ZMBs. C_10_ was carefully selected from six zwitterions to form quasi-spherical aggregates in the electrolyte, which enrich Zn^2+^/OTf^−^ and promote CIP/AGG formation via zwitterionic effects, thereby inducing unconventional localized high-concentration electrolyte (LHCE). Moreover, C_10_ self-assembles on the EDL to form a bilayer structure that acts as ionic channels, enabling smooth Zn^2+^ deposition and suppressing side reactions

Herein, we first demonstrate a self-assembled ordered nanostructure of zwitterionic additives as co-solutes to induce localized high-concentration electrolytes (LHCEs), greatly improving the reversibility of Zn metal anodes. In a sharp contrast to conventional LHCEs that rely on diluents, our approach tunes the electrolyte nanostructure via molecular self-assembly. This approach enriches the local Zn^2+^ concentration as well as creates a bilayered interfacial architecture through surface adsorption, forming ion transporting channels at the interface that can promote uniform Zn^2+^ deposition and suppress parasitic reactions. Specifically, the zwitterionic compounds contain both positively charged quaternary ammonium and negatively charged sulfonate groups, which result in promoting the formation of contact ion pairs (CIP)/aggregated ion pairs (AGG) by zwitterionic effect, thereby guiding the uniform formation of SEIs. After evaluating six kinds of zwitterionic compounds with different alkyl chain lengths, we confirmed that the decyl chain zwitterionic compound of 3-(decyldimethylammonio)propanesulfonate salt (C_10_) provided the optimally self-assembled ordered structure to form selective ion channels within the EDL (Fig. 1). Our in-depth structural information confirmed the nanostructured characteristics of the self-assembled structure, which could improve both ion carrier dynamics and local dielectric environment. Notably, C_10_ also establishes a new hydrogen-bonding network with water molecules, suppressing the occurrence of the HER and stabilizing the interphase. These synergistic effects significantly enhance long-term cycling stability, highlighting the potential of self-assembled additives to advance the practical applicability of aqueous ZMBs toward next-generation energy storage solutions.

## Results and Discussion

### Organization of C_10_ and the Associated LHCEs

In this study, we screened six zwitterionic compounds consisting of quaternary ammonium and sulfonate groups, varying the length of the alkyl chain attached to the quaternary ammonium group: ethyl, octyl, decyl, dodecyl, tetradecyl, and hexadecyl, respectively, as referred to C_2_, C_8_, C_10_, C_12_, C_14_, and C_16_ (Fig. 1). In the initial screening process, five zwitterionic additives of C_8_, C_10_, C_12_, C_14_, and C_16_ with long carbon chains were chosen considering their self-assembly into the ordered nanostructure due to their amphiphilic characteristics (Fig. S1). Given by that the shorter carbon chain was less favorable for the formation of ordered structures, C_2_ was excluded from the initial screening and used for further comparative analyses. We observed that C_16_ exhibits stronger hydrophobic interactions due to its longer carbon chain, making it quasi-solid and poorly soluble at room temperature (Fig. S2). The similar carbon chain lengths of C_8_, C_10_, C_12_, and C_14_ are expected to exhibit comparable properties. Accordingly, we conducted preliminary screening of these compounds by adding 1 molality (mol kg^−1^) of C_x_ co-solutes to 2 molality of Zn(OTf)_2_ electrolyte, referred to as Zn(OTf)_2_/C_x_. Under the identical current density of 5 mA cm^−2^, the symmetric cells in Zn(OTf)_2_/C_10_ demonstrated the most stable cycling performance (Fig. S3), indicating the optimum length of the zwitterionic additive. Considering the significant impact of alkyl chain length differences on the formation of ordered structure, therefore, C_2_ and C_10_ were used to investigate the electrolyte environment and interfacial behavior.

**Fig. 2:**
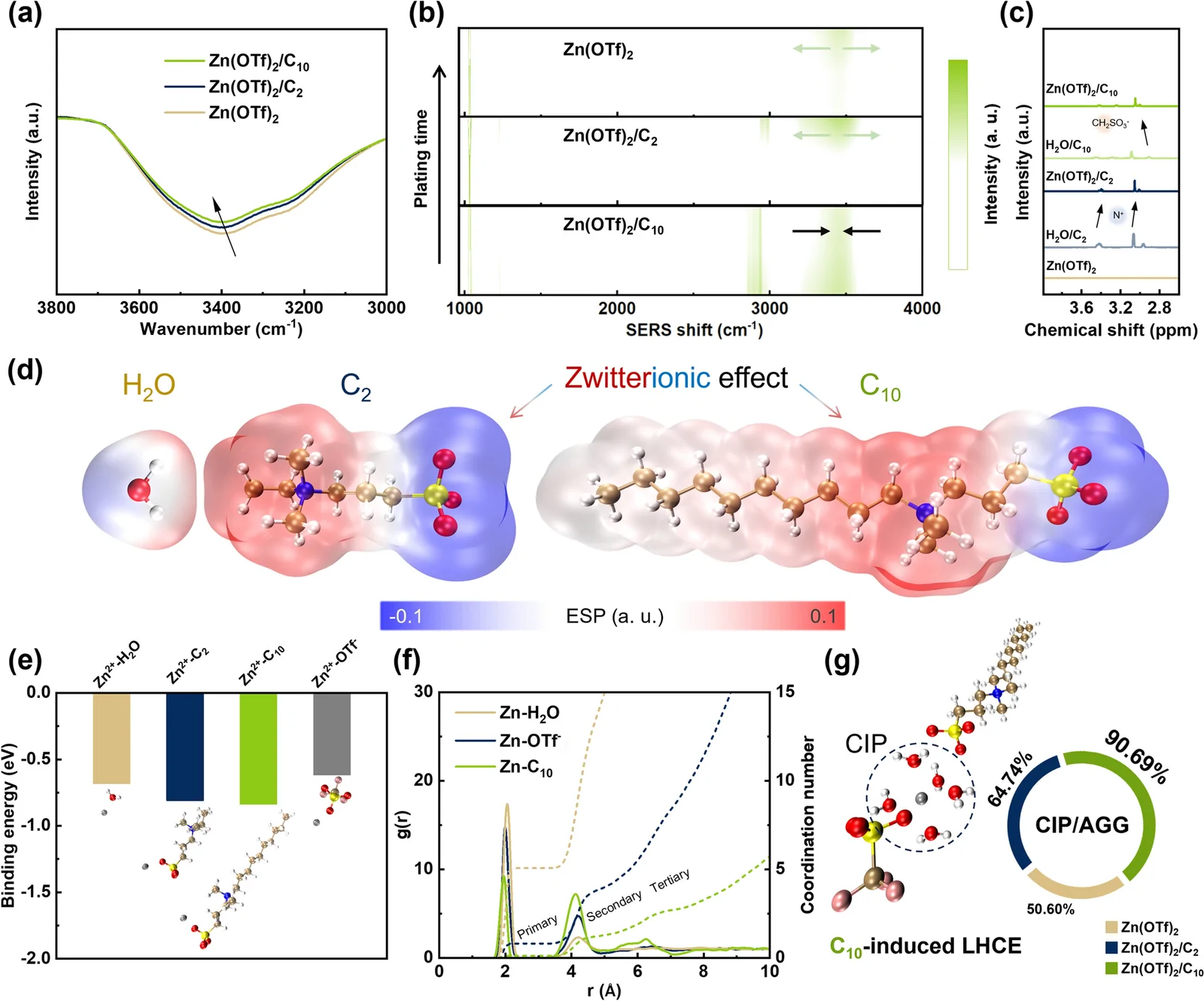
Analysis of the solution environment for different electrolytes. **a** FTIR spectra and **b** SERS spectra of different electrolytes. **c** ^1^H NMR spectra of Zn(OTf)_2_ and C_2_/C_10_ solution with or without Zn(OTf)_2_. **d** ESP mappings of H_2_O, C_2_ and C_10_. **e** Binding energies of Zn^2+^–H_2_O, Zn^2+^–C_2_, Zn^2+^–C_10_, and Zn^2+^-OTf^−^. **f** Radial distribution functions and corresponding coordination numbers of Zn^2+^–O in Zn(OTf)_2_/C_10_. **g** Schematic illustration of CIP, and the ratios of CIP/AGG in all solvation structure in different electrolytes

The combined analyses of Fourier transform infrared spectroscopy (FTIR), surface-enhanced Raman spectroscopy (SERS), nuclear magnetic resonance spectroscopy (NMR), molecular dynamics (MD) simulations, advanced synchronous small-angle X-ray scattering (SAXS), etc. were employed to meticulously investigate the impact of the C_10_ co-solute on Zn^2+^ solvation structure and the solution environment. As illustrated in Fig. 2a, the peak corresponding to the H − O stretching vibration of the electrolyte in the range of 3000 to 3800 cm^−1^ exhibits a significant blue shift upon the introduction of C_10_ into 2 molality of Zn(OTf)_2_. This observation indicates that C_10_ participates in the HB network of water molecules, thereby weakening the HB interactions among free water molecules and mitigating the risk of HER [29]. Although C_2_ also induces a blue shift, it becomes less pronounced, implying a comparatively weaker inhibition of HER. Additionally, a new peak was observed at around 1465 cm^−1^ in both Zn(OTf)_2_/C_2_ and Zn(OTf)_2_/C_10_, which corresponds to the C−H bending vibration of the − CH_2_ group (Fig. S4) [30]. Raman spectra further corroborated the FTIR findings, revealing the C−H bending vibration at 1465 cm^−1^ and a strong C−H stretching vibration peak around 2900 cm^−1^ (Fig. S5) [31].

As verified by the atomic-scale vibrational modes via SERS, the peak intensity of H − O stretching vibration for the C_2_-containing electrolyte at around 3500 cm^−1^ becomes stronger during the plating process in a similar manner to the bare electrolyte (Fig. 2b). In contrast, C_10_-containing electrolyte demonstrates a weakening trend, which suggests that C_10_ may effectively prevent water molecules from migrating into the EDL under the applied electric field. Consequently, the local concentration of water molecules in the EDL is enough low to reduce the O − H peak intensity, which indicates the formation of localized high-concentration electrolytes for the inhibition of HER and Zn dendrite growth. This finding is attributed to the formation of an organized structure of C_10_ on the EDL through self-assembly, further hindering the entry of water molecules into the EDL for the suppression of undesired parasitic reaction [32]. Furthermore, the presence of alkyl chains leads to the emergence of peaks associated with C−H stretching vibrations near 2800 cm^−1^. During a plating process, the intensity of C−H peak for the C_10_-containing electrolyte decreases, while that for the C_2_-containing one increases. This indicates that the long carbon chain in C_10_ exhibits relatively low exposure representing weaker C−H signals, while C_2_ is more active, making its C−H bonds more exposed during detection. This behavior will be further investigated in the synchronous SAXS section as below.

The solvation structure of C_10_-containing electrolyte was analyzed using ^1^H NMR (Fig. S6). The chemical shift for the hydrogen signal of water molecules in Zn(OTf)_2_, originally at 4.8273 ppm, shifts to a higher field at 4.8198 ppm upon the addition of C_10_. This upfield shift is attributed to the proton shielding effect on the reduced activity of free water and the reconfiguration of the HB network [33]. Figure 2c shows the high-field ^1^H NMR spectra of Zn(OTf)_2_, Zn(OTf)_2_/C_2_, and Zn(OTf)_2_/C_10_, as well as aqueous C_2_ and C_10_ solutions without Zn salts. For both C_2_ and C_10_, the chemical shifts of the hydrogen of the CH_2_SO_3_^−^ group between 2.9 and 3.0 ppm shifted downfield upon the addition of Zn salt, while those of the alkyl hydrogen surrounding N^+^ shifted upfield. This unique shift behavior arises from the zwitterionic nature. The downfield shift of the CH_2_SO_3_^−^ peak indicates the strengthened interaction between Zn^2+^ and the SO_3_^−^ group, resulting in altering the chemical shift arising from a more electron-deficient environment for the H of CH_2_. By contrast, the upfield shift of the alkyl hydrogen surrounding N^+^ indicates more electron-rich environment due to the interaction between N^+^ and OTf^−^. Accordingly, the electron density around N^+^ increases, making it more strongly attracted to the electron cloud of the surrounding H atoms. These interactions between the zwitterionic compounds and Zn^2+^/OTf^−^ effectively bring Zn^2+^ and OTf^−^ into closer spatial proximity, promoting the formation of contact ion pairs (CIP, Zn^2+^ primary solvation structures involving direct anion coordination) and even aggregations (AGG, larger solvation structures where a single anion coordinates with two or more Zn^2+^ ions). The increase in both CIP and AGG and the corresponding reduction in solvated single ion pairs (SSIP, Zn^2+^ primary solvation structures dominated by solvent coordination) implies a locally high Zn^2+^ concentration in the electrolyte, which can improve the reversibility of Zn deposition [34].

Electrostatic potential (ESP) mapping for H_2_O, C_2_, and C_10_ was performed to visualize the distribution of surface charge (Fig. 2d). Both C_2_ and C_10_ exhibit distinct positive and negative electrostatic regions centered around the N^+^ and SO_3_^−^ groups, respectively, further demonstrating their zwitterionic effect in the electrolyte environment. For C_10_, the distal region of the long alkyl chain—away from the N^+^ group—looks nearly white in the ESP mapping, indicating a near-zero electrostatic potential. This suggests that the alkyl chain primarily serves as a spatial modulator or nanoenvironmental regulator, rather than directly participating in solvation.

DFT calculations were carried out to evaluate the binding energies in a solvated environment (Fig. 2e). The binding energy of Zn^2+^–H_2_O (− 0.682 eV) is notably weaker than that of Zn^2+^–C_2_ (− 0.811 eV) and Zn^2+^–C_10_ (− 0.837 eV). These results indicate that Zn^2^⁺ preferentially coordinates with the zwitterionic additives rather than with water molecules. Additionally, the binding energy of Zn^2+^–OTf^−^ was calculated to be − 0.617 eV. This further explains why, in electrolytes with lower zinc salt concentrations (≤ 2 molality), the six-water-solvation shell is more prevalent than the CIP, which corresponds to the case of OTf^−^ participating in solvation as discussed in this paper.

To further elucidate the solvation environment, MD simulations were performed (Fig. 2f). As verified by coordination number (CN), C_10_ is barely involved in the primary solvation structure, primarily participating in the secondary solvation structure. Simultaneously, it promotes the incorporation of OTf^−^ into the primary solvation structure, thereby increasing the proportion of CIP/AGG. Specifically, the proportion of such environments relative to the total coordination of Zn^2+^ increased from 11.8% in the blank electrolyte to 13.9% in C_10_-containing one (Fig. S7). This effect was attributed to the steric hindrance and spatial modulation of the nanostructured C_10_ with the long alkyl chain, as well as its ability to locally alter the electron density of the solvation environment. These factors likely promote electrostatic interactions between OTf^−^ and Zn^2+^, facilitating the formation of stronger coordination through improved orbital overlap and polarization of the OTf^−^ electron cloud. To confirm this underestimation, we performed the deconvolution of the symmetric stretching mode of SO_3_^2–^ peak in Raman spectra (Figs. 2g and S8) [35, 36]. For Zn(OTf)_2_/C_10_, the proportion of CIP/AGG reached 90.69%, significantly higher than 64.74% in Zn(OTf)_2_/C_2_ and 50.60% in Zn(OTf)_2_.

It is well known that locally high-concentration electrolytes (LHCEs) combine the advantages of both dilute electrolytes and highly concentrated electrolytes (HCEs), with representative coordination structures gradually transitioning from SSIP to CIP/AGG [37]. Therefore, the increased CIP/AGG proportion in the presence of C_10_ suggests that it induces a locally concentrated environment by restructuring the primary solvation structure, which could enhance ionic transport and suppress parasitic reaction. Interestingly, C_2_ also predominantly resides in the secondary solvation structure, while C_10_ exhibits an additional distinct tertiary solvation structure. This observation suggests that C_10_ may induce a high-density, spatially periodic nanoenvironment around Zn^2+^ for the unique LHCEs. This intriguing finding will be further discussed in detail below.

**Fig. 3:**
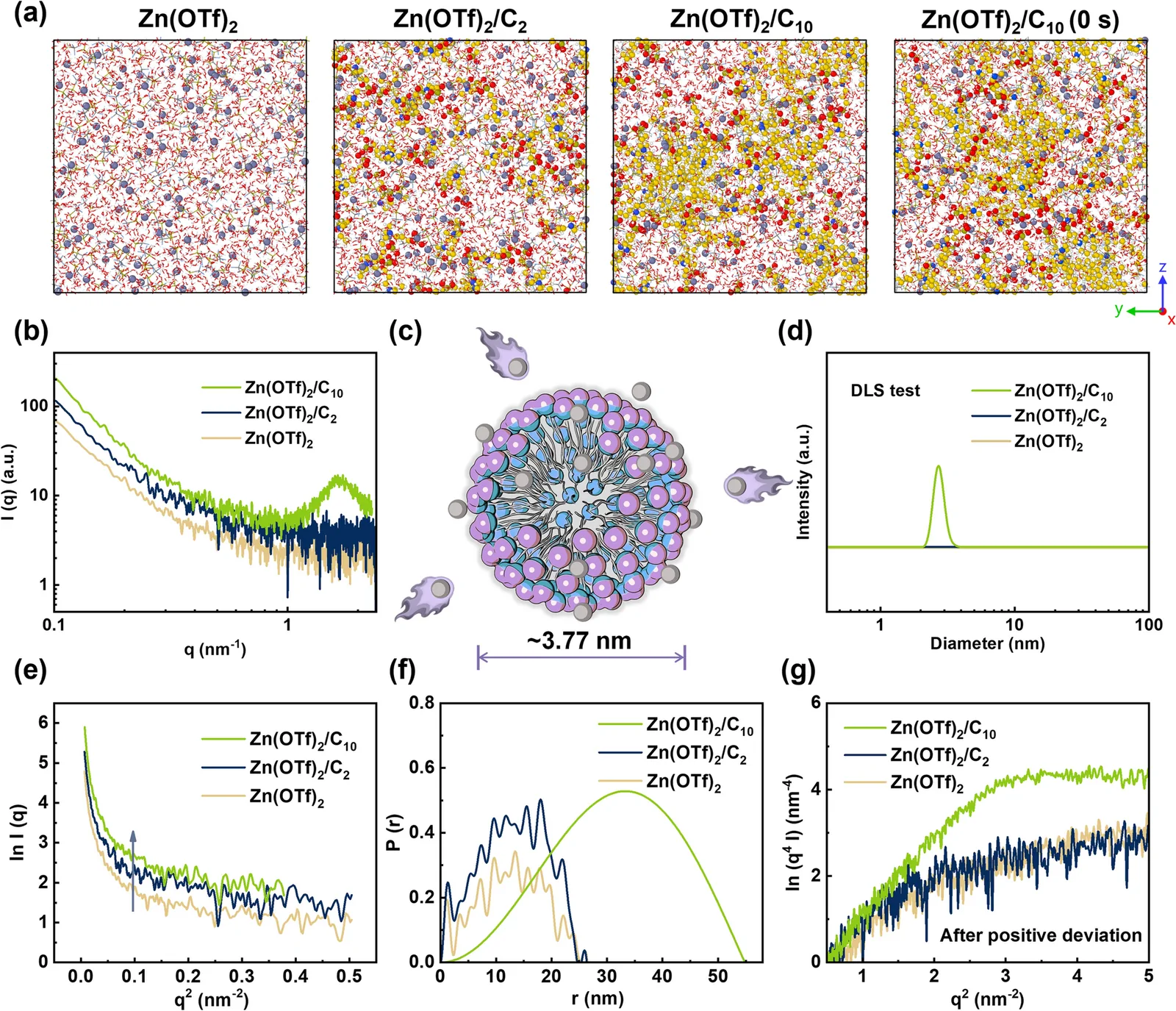
Aggregation behavior of C_10_ in the electrolyte environment. **a** Snapshots of different electrolytes after 15 ns, and Zn(OTf)_2_/C_10_ at 0 s. **b** Synchronous SAXS spectra (one-dimensional scattering plots) for different electrolytes. **c** Schematic illustration of the C_10_ aggregation. **d** DLS spectra, **e** Guinier plots, **f** PDDF plots and **g** Porod plots for different electrolytes

A temporal and spatial analysis of the MD snapshots was performed to comprehensively examine whether C_10_ forms large-scale aggregates or not. As shown in Fig. 3a and Fig. S9, C_10_ exhibits local aggregation after 15 ns of MD simulation, whereas C_2_ remains uniformly distributed throughout the solvent box. This aggregation is absent in the initial state of C_10_, indicating that it emerges during the course of the simulation. Additionally, Zn^2+^ ions are randomly distributed in both the Zn(OTf)_2_ and Zn(OTf)_2_/C_2_ systems. Nevertheless, Zn^2+^ ions in the Zn(OTf)_2_/C_10_ system are predominantly localized around the C_10_ aggregates, suggesting that the aggregation of C_10_ promotes the formation of locally high Zn^2+^ concentrations.

Building upon the MD simulation results, we have further conducted a statistical analysis based on probability distribution to quantitatively evaluate the relative proportion of AGG within the CIP/AGG configurations in the primary solvation structure of Zn^2+^ involving OTf^–^ (Fig. S10). In the pristine Zn(OTf)_2_ electrolyte, AGG accounts for only 1.5%. On the other hand, the introduction of zwitterionic compounds significantly reconstructs the ion association landscape: The AGG ratio increases to 5.5% in the Zn(OTf)_2_/C_10_ system, and it further surges to 11.1% in the Zn(OTf)_2_/C_2_. These results clearly demonstrate that the formation of localized high-concentration regions depends on the aggregation of zwitterions. Although the AGG proportion in the C_2_-containing system is higher than that in C_10_, C_2_ exhibits superior dispersibility, whereas C_10_ tends to spontaneously form aggregates. As a result, the actual AGG-inducing capability of C_10_ in localized domains may be substantially underestimated in global statistical analysis. To confirm this underestimation, we have further obtained evidence from the deconvolution of the symmetric stretching mode of SO_3_^2–^ peak in Raman spectra (Fig. S8). For Zn(OTf)_2_/C_10_, the proportion of AGG reached 32.11%, much higher than 13.37% in Zn(OTf)_2_/C_2_. Meanwhile, Zn(OTf)_2_/C_10_ also exhibited the lowest proportion of SSIP and the highest proportion of CIP, further supporting that self-ordered C_10_ induces LHCE formation.

As verified by the aforementioned ^1^H NMR and MD simulation results, the introduction of C_10_ facilitates the formation of CIP between Zn^2+^ and OTf^−^. Although the binding energy between C_10_ and Zn^2+^ is stronger than that between Zn^2+^ and OTf^−^, the formation of CIP is not simply governed by static binding energy. Instead, it is governed by multiple factors, including local ion ratio and spatial structural stability. Therefore, the enhancement of CIP proportion by C_10_ has profound practical implications. To further reveal the effect of C_10_ on the intrinsic coordination tendency of Zn^2+^ under locally enriched conditions, we have constructed MD simulation about a water-free, high-C_10_-concentration system (Fig. S11). It is found that the coordination number of Zn^2+^ with OTf^−^ approaches 2, matching the stoichiometry of Zn(OTf)_2_, thereby confirming that C_10_ promotes OTf^−^ coordination through spatial regulation rather than direct competition (Fig. S12). Therefore, these findings confirm a new ion-structuring mechanism, which is totally different from that of conventional LHCEs. Specifically, the zwitterionic C_10_ molecules tend to be self-assembled into forming localized domains. These domains further promote the local enrichment of Zn^2^⁺ and OTf⁻ through strong electrostatic and dipole interactions. Such cooperative assembly not only leads to the formation of CIP/AGG, but also enables to manipulate interfacial nanostructures for favorable electrochemical environments (Fig. 1).

The organization of ordered nanostructure, which is key to the construction of new type LHCE, was further confirmed using synchronous SAXS analyses. As shown in the two-dimensional SAXS images (Figs. S13-S15), the scattering patterns of the C_10_-containing electrolyte exhibit a broader intensity distribution, particularly in the high-*q* region, representing an increase in small-angle scattering. This finding indicates that the aggregation of C_10_ is attributed to the formation of larger repeating structures on a nanoscale, thereby altering the distribution or assembly of colloids [38]. On the other hand, the scattering pattern of the C_2_-containing electrolyte was similar to that of the blank electrolyte, as characterized by relatively concentrated scattering rings, demonstrating no significant repeating structures. Ultraviolet–visible (UV–vis) spectroscopy further confirms the presence of self-assembly behavior. The addition of C_2_ leads to the appearance of a new peak at ~ 250 nm (Fig. S16), which is attributed to the interaction between the C_2_ and Zn(OTf)_2_, solely altering the overall energy levels and electronic structure [39]. On the other hand, C_10_ with the longer carbon chain increases the polarizability of its electron cloud. The formation of self-assembled conformations facilitates intramolecular transitions of n → σ^*^, leading to a new peak at ~ 210 nm and exhibiting more complex spectral features [40].

As shown in the one-dimensional scattering plots (Fig. 3b), only the Zn(OTf)_2_/C_10_ electrolyte exhibits a prominent peak at a scattering vector *q* of 1.67 nm^−1^, whereas the Zn(OTf)_2_/C_2_ electrolyte cannot do it. Given by Bragg’s law, C_10_ forms a periodic length of 3.77 nm in an electrolyte solution, which is nearly twice greater than the length of the C_10_ molecule itself (Fig. 3c). This assembled structure was also consistent with the hydrodynamic diameter obtained from dynamic light scattering (DLS) (Fig. 3d). Peaks corresponding to the dynamically diffusing core region of the aggregation were observed in a range from 2 to 4 nm for Zn(OTf)_2_/C_10_, whereas no such peaks were detected for Zn(OTf)_2_ and Zn(OTf)_2_/C_2_. Therefore, it is confirmed that the long chain length and zwitterionic characteristics of C_10_ contribute to showing self-assembly behaviors in an aqueous solution, resulting in regularly ordered nanostructures [41]. This self-assembly of C_10_ also provides a rationale for the weakening of C−H signals during plating, as indicated by SERS results.

We have further studied the low-q region based on the Guinier theorem (Fig. 3e). All three electrolytes display concave curves in the Guinier plots, the characteristic of polydisperse systems. The scattering intensities (ln *I*) of Zn(OTf)_2_, Zn(OTf)_2_/C_2_, and Zn(OTf)_2_/C_10_ increase sequentially, corresponding to an increasing degree of aggregation. This further confirms that the long carbon chain of C_10_ facilitates aggregation behavior in water, resulting in a higher overall scattering intensity. For an ideal polydisperse system, the ideal aggregate radius of gyration *R*_*g*_ can be derived from the Guinier theorem. A larger *R*_*g*_ corresponds to a more uniform particle distribution and higher ordering in the solution [42]. The fitted *R*_*g*_ values from small-angle region (0 to 0.1 nm^−2^) for Zn(OTf)_2_, Zn(OTf)_2_/C_2_, and Zn(OTf)_2_/C_10_ are 9.902, 10.051, and 23.541 nm, respectively (Table S1). The shorter chain length of C_2_ has a relatively minor influence on the formation of aggregates, resulting in very minor change in *R*_*g*_. On the other hand, the self-assembled structure induced by the hydrophobicity of the long carbon chain C_10_ is associated with stronger intermolecular interactions, forming the larger ordered structures that modify the electrolyte environment in a favorable manner [43].

The PDDF was analyzed for the relative positions and distributions of colloidal particles in the three electrolytes (Fig. 3f). Unlike the oscillating curves of the other two electrolytes, the curve for Zn(OTf)_2_/C_10_ is notably smooth, indicating a more uniform size distribution of aggregates, which implies stronger and more stable interactions [44]. The maximum value of the vertical axis *P(r)* signifies that C_10_ has the highest probability of forming an ordered structure. The nearly symmetric single peak shape of Zn(OTf)_2_/C_10_ elucidates that its aggregates correspond to a quasi-spherical shape as referred to previous works [45, 46]. Thus, we gain an in-depth understanding about the morphology of aggregates generated by 1 molality of C_10_ in the electrolyte. It is postulated that this structure can direct a more uniform electric field distribution, facilitating the uniform distribution of Zn^2+^ ions for the improved reversibility of Zn deposition. The as-observed *r*_*max*_ for Zn(OTf)_2_/C_10_ is 54.813 nm, significantly larger than the nearly same values of around 25 nm for both Zn(OTf)_2_ and Zn(OTf)_2_/C_2_ (Table S1). Furthermore, the value of *r*_*max*_ is nearly twice larger than that of *R*_*g*_, which confirms that C_10_ is assembled into large-scale aggregates and then anchors Zn^2+^ to achieve stability. The decay portion of the curve decreases more rapidly than the growth portion, which also implies a higher probability of generating larger stable aggregates.

Considering positive deviations in the Porod analysis for all three electrolytes due to the dynamic electronic interactions, the correction was needed, as shown in Fig. 3g. Since the aggregates in the solution feature with a near ideal two-phase system, the vertical axis was chosen for the *n* = 4 state [47]. Obviously, only Zn(OTf)_2_/C_10_ exhibits a plateau, further confirming that C_10_ forms aggregates with certain rigidity. On the other hand, Zn(OTf)_2_ and Zn(OTf)_2_/C_2_ show continuous growth, indicating the intrinsic disorder [48]. Additionally, Zn(OTf)_2_/C_10_ shows a higher value on the vertical axis at the plateau compared to the other two electrolytes. This is attributed to the strong interactions among C_10_, Zn^2+^, and OTf^−^, which lead to a rearrangement of the electron cloud. As a result, Zn^2+^ is shed out from its traditional six-hydrated Zn^2+^ solvation shell, leading to a higher local concentration of Zn^2+^ within the self-assembled larger aggregates. These aggregates exhibit structural rigidity and provide new ion transporting pathways [49]. Obviously, these results are the first report about the self-assembled ordered nanostructure of zwitterionic compounds associated with the formation of LHCEs as verified by comprehensive MD, Raman spectra, UV–Vis spectra, DLS, SAXS images, one-dimensional scattering, Guinier, PDDF, and Porod techniques.

### Investigation of C_10_ on Parasitic Reactions and Interphasial Structure

**Fig. 4:**
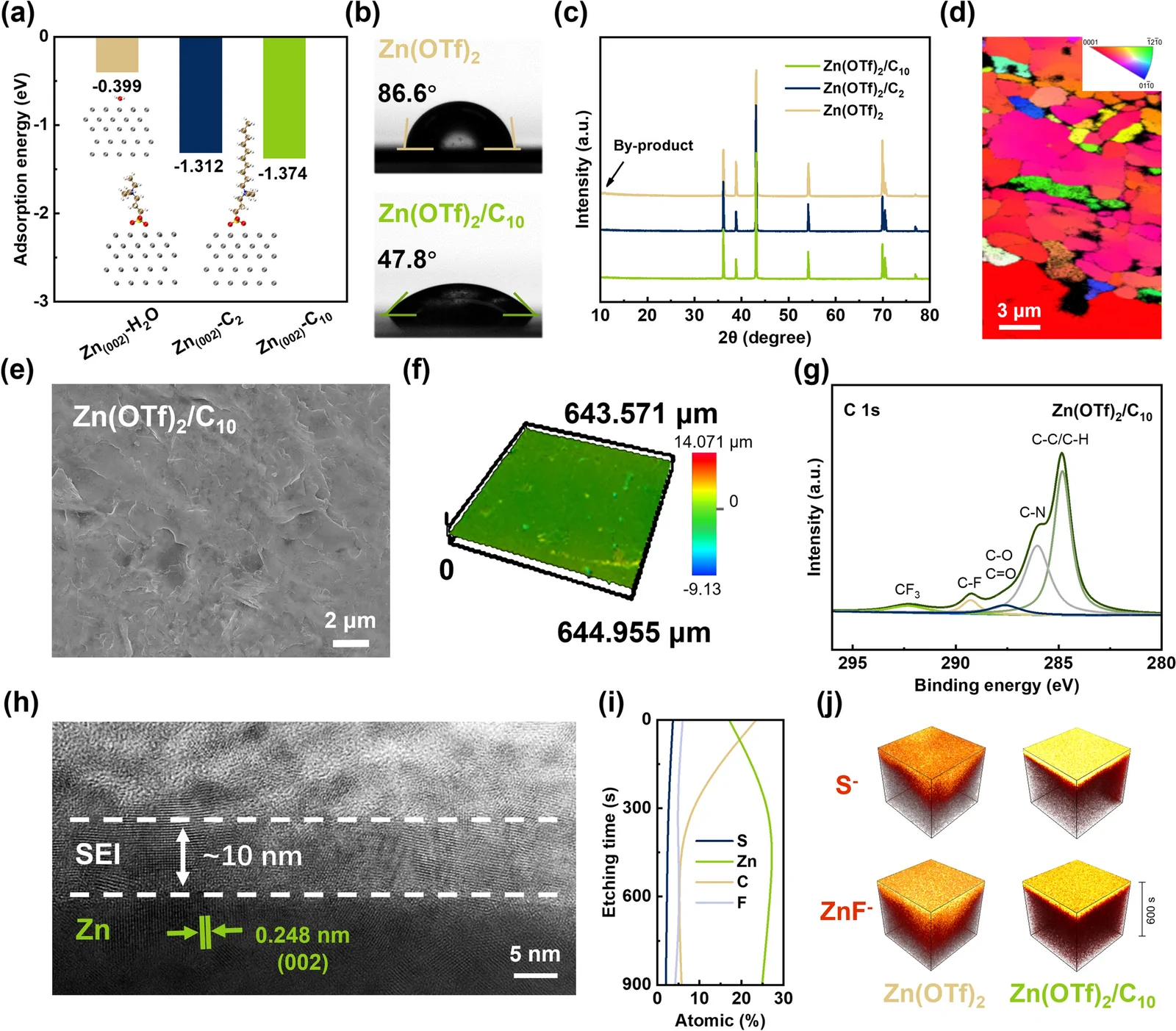
Characteristics of the Zn anodes after cycling in different electrolytes. **a** Adsorption energies of H_2_O, C_2_ and C_10_ with the Zn (002) plane. **b** Contact angles of Zn(OTf)_2_ and Zn(OTf)_2_/C_10_ on bare Zn foil. **c** XRD images of Zn after plating/stripping for 20 times in different electrolytes. **d** EBSD orientation map, **e** SEM image, **f** LSCM image, **g** XPS C 1*s* spectra and **h** cross-section HR-TEM image of Zn after plating/stripping for 20 times in Zn(OTf)_2_/C_10_. **i** Variation of atomic with etching time. **j** ToF–SIMS spectra of Zn after plating/stripping for 20 times in Zn(OTf)_2_ and Zn(OTf)_2_/C_10_

Along with the solution environment, the interphasial structure was investigated performing DFT calculations on the adsorption energies of different molecules on the Zn (002) plane (Fig. 4a). The adsorption energy of H_2_O was calculated to be −0.399 eV, while those of C_2_ and C_10_ were −1.312 and −1.374 eV, respectively. These results indicate that the zwitterions exhibit a much stronger affinity with the Zn (002) surface compared to H_2_O molecules, suggesting that they can be preferentially adsorbed onto the electrode surface. This preferential adsorption enables effective modulation of the electrical double layer (EDL), thereby suppressing side reactions such as hydrogen evolution and corrosion.

The improved Zn affinity of the electrolyte by C_10_ was confirmed measuring the instantaneous contact angle. As shown in Fig. 4b, the addition of C_10_ reduced the contact angle between the blank electrolyte and Zn foil from 86.6° to 47.8°, demonstrating a significant improvement in zincophilicity and electrolyte wettability [50]. To further validate the contact angle measurements in Fig. 4b, we have provided videos of an additional set of in situ tests (Videos S1 and S2), with the contact angle evolution recorded over 0–20 s (Fig. S17). As clearly shown, our C_10_ exhibits excellent wettability toward the Zn surface, and its strong zincophilicity is beyond question. In order to verify the interfacial structure adsorbed by the organized C_10_, the contact angle between de-ionized water and the Zn foil was measured after immersing the latter in the electrolyte. The Zn foil treated with the C_10_-containing electrolyte exhibited a contact angle of 22.9°, much lower than 40.7° with the blank electrolyte (Fig. S18). Notably, the improved wettability, in a conjunction with DFT calculations, suggests that the Zn surface adopts an ordered arrangement of C_10_ at the interface in a similar manner to the "lipid bilayer" structure found in biological membranes, where hydrophilic groups are oriented outward [51]. This unique interfacial structure creates ionic channels that facilitate uniform and facile Zn ion transfer for high reversibility of Zn deposition.

To verify the adsorption of C_10_, capacitance-potential measurements were carried out for electrolytes with and without C_10_. As shown in Fig. S19, the presence of C_10_ leads to a significant decrease in differential capacitance across all potentials, indicating the formation of a stable adsorbed layer. This behavior is consistent with our proposed mechanism in which C_10_ forms ordered ion channels at the interface.

The capability of C_10_ in suppressing side reactions was analyzed through XRD spectra (Fig. S20). After immersing in the Zn(OTf)_2_ electrolyte for 7 days, two by-products of ZnO and Zn_x_(OTf)_y_(OH)_2x−y_·nH₂O were observed in the blank electrolyte. While reducing the corrosion risk upon the addition of C_2_, a small amount of Zn_x_(OTf)_y_(OH)_2x−y_·nH_2_O still remains intact. In contrast, the addition of C_10_ effectively protected the Zn foil from corrosion, without any by-product signals. As illustrated in Fig. 4c, the Zn(OTf)_2_/C_10_ electrolyte after 20 cycles inhibited the formation of by-products, demonstrating an increased intensity ratio of (002) to (101) planes from 0.34 to 0.40. This suggests that Zn tends to be grown laterally parallel to the (002) plane for compact deposition [52]. Electron backscatter diffraction (EBSD) in Fig. 4d further supports this observation, where the (002) plane is clearly dominant. In contrast, the Zn anode cycled in Zn(OTf)_2_ exhibits a distinct (101) plane deposition pattern. Accordingly, the deposition of (002) plane is indeed induced by the interfacial structure modified by the ordered C_10_. Furthermore, the uneven Zn deposition and strong side reactions in Zn(OTf)_2_ lead to significant formation of zero solutions (no valid results) (Fig. S21).

The electron microscopy images provide clear evidence of this observation. First, SEM images of the Zn foils were observed after immersing them in the electrolyte for 7 days. The existence of hexagonal by-products and corrosion on the Zn foil were detected in the blank electrolyte (Fig. S22). In contrast, the interfacial regulation of C_10_ allowed the Zn foil to maintain its flat and smooth surface (Fig. S23). Through EDX mapping (Fig. S24), S, C, and N elements were identified and their distributions were observed on the Zn surface, which indicates that the uniform adsorption of C_10_ onto the electrode surface effectively suppresses corrosion and HER. As shown in SEM images of the Zn foils after 20 plating/stripping cycles, the addition of C_10_ was attributed to a tightly packed, uniform, and dense surface of Zn, ensuring a dendrite-free deposition (Figs. 4e and S25). On the other hand, the Zn deposited in the blank electrolyte exhibited an uneven, randomly stacked vertical structure due to uncontrolled dendrite growth. After immersion in the Zn(OTf)_2_/C_2_ electrolyte, by-products were observed on the surface of the Zn foil (Figs. S26 and S27). After plating/stripping, the Zn anode displayed random stacking in the Zn(OTf)_2_/C_2_ electrolyte and failed in achieving the horizontally aligned deposition behavior. The smooth and uniform Zn deposition induced by C_10_ was further confirmed using laser scanning confocal microscopy (LSCM) images (Figs. 4f and S28). The smoothness of the Zn anode surface was significantly enhanced by the addition of C_10_, while the numerous dendritic protrusions were observed in the blank electrolyte. This smooth deposition surface is associated with a uniform ionic flux and electric field to avoid “tip effects” and concentration polarization, which highlight the importance of ordered structure by C_10_ for the reversible Zn deposition. This conclusion is further supported by the surface roughness Sa, with Zn foils cycled in Zn(OTf)_2_ showing a value of 1.331 μm, which dramatically decreased to 0.586 μm upon the addition of C_10_.

XPS was applied to investigate the surface chemistry of the cycled Zn foil as shown in Fig. 4g. The surface adsorption of C_10_ drastically increased the ratio of C−C to C−H at ~ 285 eV compared to the blank electrolyte (Fig. S29) [53]. This surface adsorption contributes to the modification of inner Helmholtz layer, providing a unique Zn^2+^ transporting pathway and suppressing a direct contact of active water with the electrode surface. Although the adsorption of C_2_ is associated with an increase in the ratio of C−C to C−H, this enhancement became less pronounced than that with C_10_ (Fig. S30).

It has been well known that Zn(OTf)_2_ can form a solid electrolyte interphase (SEI) on the Zn anode surface containing F and S elements [54]. As shown in the in-depth F 1*s* XPS spectra (Figs. S31-S33), CF_3_ and ZnF_2_ signals at 688 and 684 eV were still detectable in the blank electrolyte at an etching depth of 300 s. However, these signals were faded with further etching, representing a non-uniform SEI [55]. By contrast, these CF_3_ and ZnF_2_ signals are strong in the electrolytes containing C_2_ and C_10_, even after etching to a depth of 900 s, indicating more uniform F-rich SEIs. Furthermore, in-depth S 2*p* XPS spectra demonstrate that only the C_10_-containing electrolyte shows obvious ZnS signals at around 164 eV throughout all etching depths (Figs. S34-S36) [56]. Thus, these findings indicate the synergistic effect of CIP/AGG formation by the C_10_ zwitterionic compounds on the uniformity of the robust SEI containing both F and S elements and the fast ion channels by interfacially adsorbed C_10_.

The cross section of the cycled Zn electrode was examined using focused ion beam (FIB) cutting and HR-TEM (Fig. 4h). The flatness of the Zn surface was significantly improved by C_10_, along with a smooth SEI of ~ 10 nm thickness. Through the elemental radial distribution analysis (Fig. 4i), the contents of S and F show minimal variations in the depth profile, indicating the uniformity of the SEI. The 3D ToF–SIMS spectra further confirm the homogeneous existence of the SEI (Fig. 4j), which implies that C_10_ promoted the formation of uniform SEI composed of ZnS and ZnF_2_. On the other hand, the non-uniform SEI formed in the blank electrolyte led to cycling fluctuations and heterogeneous distribution of Zn, ultimately causing rapid battery deactivation [57]. The 2D ToF–SIMS spectra further reveal that in the presence of C_10_, both the S⁻ and ZnF⁻ signals level off at around 100 s of etching, whereas in the blank electrolyte these signals continue to decrease throughout 600 s (Fig. S37). This further indicates that C_10_ enables the formation of a more stable and homogeneous SEI. Therefore, the zwitterionic C_10_ at the interface not only modulates the inner Helmholtz layer to mitigate parastitic reactions but also guides the stable horizontal deposition of Zn^2+^, further highlighting the self-assembling strategy for LCHEs.

### Electrochemical Performance of Zn Metal Batteries in C_10_ Containing LHCEs

**Fig. 5:**
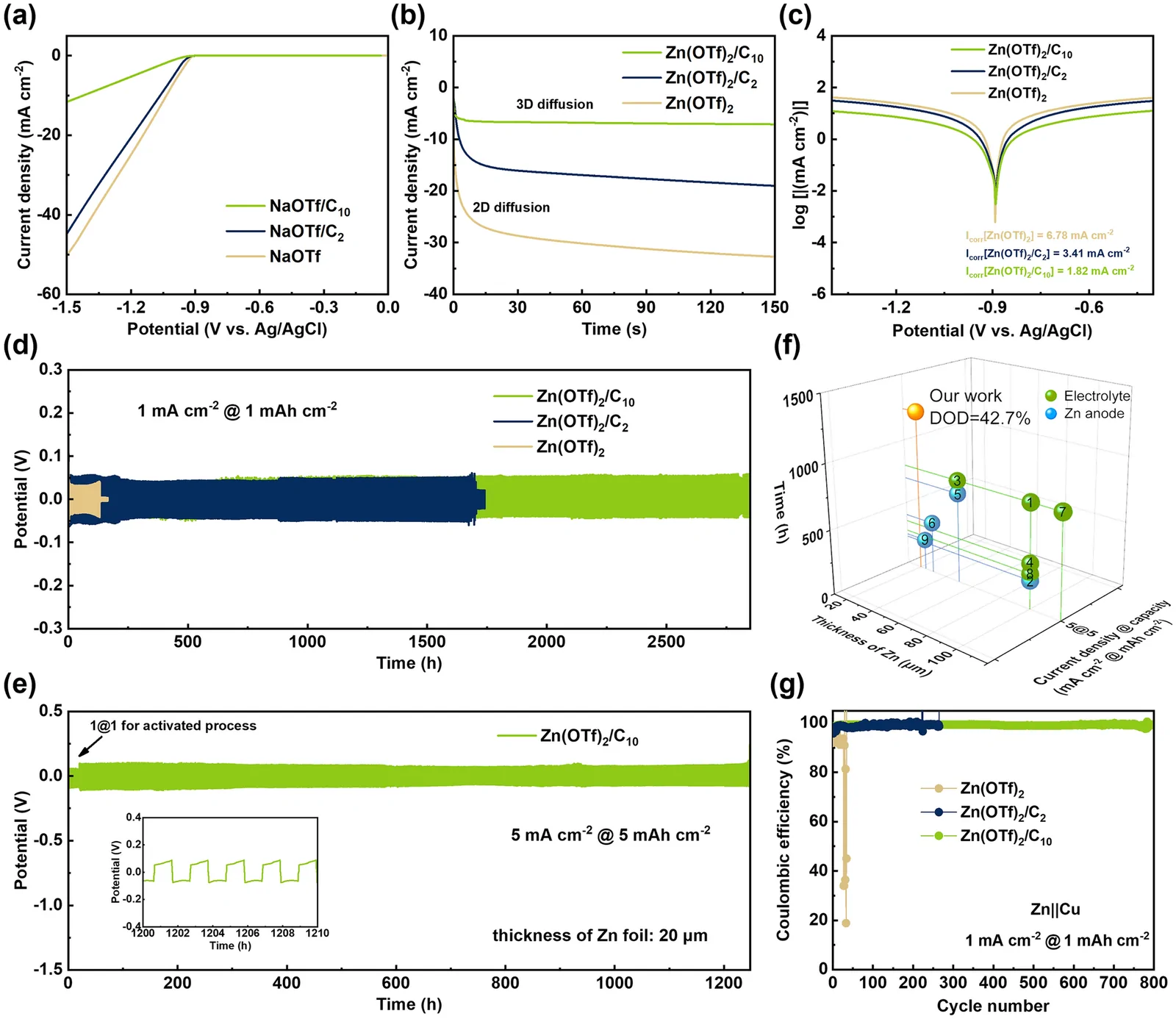
Electrochemical tests and cycling performances of different electrolytes. **a** LSV curves, **b** CA curves and **c** Tafel curves of different electrolytes. **d** Cycling performances of Zn||Zn symmetric cells in different electrolytes at 1 mA cm^−2^ for 1 mAh cm^−2^. **e** Cycling performances of Zn||Zn in Zn(OTf)_2_/C_10_ at 5 mA cm^−2^ for 5 mAh cm^−2^. **f** Comparison of the thickness and cycling time with recently reported Zn anodes using optimization strategies at 5 mA cm^−2^ for 5 mAh cm^−2^. **g** CE of Zn||Cu asymmetric cells in different electrolytes

The superiority of C_10_ was verified through electrochemical characterizations. The LSV curves were employed replacing Zn(OTf)_2_ by NaOTf to exclude the influence of Zn deposition (Fig. 5a). When the response current density reached − 10 mA cm^−2^, the potential of NaOTf/C_10_ was dramatically dropped by about − 1.4 V, much higher than those of NaOTf and NaOTf/C_2_. This observation is attributed to a signficant disruption of the hydrogen-bonding network consisting of free water and thus a more effective inhibition of HER. CA curves reveal C_10_'s capability to guide Zn deposition (Fig. 5b). Zn(OTf)_2_/C_10_ exhibits a stable 3D diffusion pattern of CA curve, mitigating the risk of dendritic growth associated with 2D diffusion [58]. Although Zn(OTf)_2_/C_2_ achieved a smoother curve compared to the blank electrolyte, it still displayed a significant portion of 2D diffusion, leading to less stable Zn deposition. The Tafel curves show that, with the addition of C_2_ and C_10_, the corrosion current (I_corr_) of Zn(OTf)_2_ decreased from 6.78 to 3.41 and 1.82 mA cm^−2^, respectively (Fig. 5c), indicating that both co-solutes have the capability to inhibit corrosion. Consistently, measurements of the mass change of Zn immersed in different electrolytes further demonstrate that C_10_ provides the most effective corrosion suppression (Fig. S38). Nonetheless, C_10_ is more effective for suppressing the corrosion of the Zn electrode, which is in a good agreement with the anti-corrosion results observed in the SEM and XRD analyses.

We systematically investigated the ionic conductivity, viscosity, and Zn^2+^ transference number (t_Zn_) of the electrolytes (Fig. S39) to ensure optimal Zn^2+^ transport properties. Since Zn(OTf)_2_/C_16_ is solid at room temperature, it was heated to 40 °C for comparison with other samples. The ionic conductivities of Zn(OTf)_2_, Zn(OTf)_2_/C_2_, Zn(OTf)_2_/C_10_, and Zn(OTf)_2_/C_16_ gradually decreased with increasing alkyl chain length, from 67 to 53, 48, and 39 mS cm^−1^, respectively. Simultaneously, the viscosity increased from 0.8 to 10.1 mPa s. This trend is consistent with the typical behavior of electrolytes when an amount of organic components increases [59]. On the other hand, t_Zn_ values increased significantly, from 0.24 in the blank system to 0.46 in the C_2_ system, and further to 0.51 in the C_10_ system (Fig. S40). This phenomenon once again confirms that the ordered structure of C_10_ effectively enhances the local concentration of Zn^2+^, improving the contribution of Zn^2+^ to the overall conductivity. Moreover, the self-assembled nanostructures formed by C_10_ provide more orderly migration pathways for Zn^2+^, suppressing the free diffusion of other ions and creating selective ion channels.

The symmetric Zn||Zn cells were used to investigate the improved reversibility of Zn plating/stripping in the C_10_-containing electrolyte. In order to determine the optimal concentration, the concentrations of C_10_ were controlled into 0.5, 1.0, and 1.5 molality, respectively. The Zn||Zn cells exhibited cycling stabilities of 690, 2800, and 760 h, respectively, at a current density of 1 mA cm^−2^ and a capacity of 1 mAh cm^−2^ (Fig. S41). The best stability of C_10_ at 1 molality might be attributed to a well-balanced system with a proper ionic strength. At lower concentrations, it failed in forming the well-defined aggregated structures for LHCEs, while at higher concentrations, viscosity signficantly increased for sluggish kinetics, both of which hinder the facile and reversible kinetics of Zn deposition.

The electrochemical performances of the symmetric cells were further compared in the pristine and modified electrolytes at the identical concentration. The symmetric cells in Zn(OTf)_2_ failed after 138 h at 1 mA cm^−2^ and 1 mAh cm^−2^ (Fig. 5d). On the other hand, the symmetric cells in Zn(OTf)_2_/C_2_ and Zn(OTf)_2_/C_10_ demonstrated long-term plating/striping stabilities of approximately 1700 and 2800 h, respectively. The existence of C_10_ slightly increased the overpotential in a marginally acceptable range owing to the improved CIP/AGG formation and t_Zn_, which compromise the adverse effects of higher viscosity and lower ionic conductivity. Additionally, the self-assembly behavior of C_10_ contributed to an elevated local concentration of Zn^2+^, resulting in a higher t_Zn_. To further validate the advantages of C_10_, Zn plating/stripping was evaluated under severe conditions of 5 mA cm⁻^2^ and 5 mAh cm^−2^ with a 20 μm thick Zn anode (DOD of 42.7%). After initial activation for 10 cycles, the Zn||Zn cells in Zn(OTf)_2_/C_10_ lasted over 1200 h (Fig. 5e), greatly outperforming those in Zn(OTf)_2_ and Zn(OTf)_2_/C_2_. Figure 5f compares the DOD and cycling life of the symmetric Zn||Zn cells at high current density and deposition capacity (5 mA cm^−2^ and 5 mAh cm^−2^) with recently published works about electrolyte modifications and Zn anode protection for ZMBs, as summarized in Table S3. Our C_10_-containing cells exhibited the highest DOD (42.7%) and longest cycling life (over 1200 h), demonstrating the strongest competitive performance.

In situ EIS measurements were conducted in the symmetric cells to investigate the electrode kinetics (Fig. S42). As shown in the open-circuit voltage (OCV) plot, the surface-adsorbed zwitterionic species significantly reduced the interfacial charge transfer resistance (R_ct_). After cycling, the R_ct_ of C_10_ decreased significantly, whereas the decrease in C_2_ was relatively small. Additionally, C_10_ gradually exhibited the superposition of two semicircles, indicating that the formation of a new interface is due to the influence of large aggregates on the EDL, which was not observed in Zn(OTf)_2_ or in Zn(OTf)_2_/C_2_ without self-assembly. The pause in the plating/stripping process during the in situ testing, which is caused by the EIS measurement step, is attributed to a short circuit in the Zn(OTf)_2_ after 50 cycles, highlighting its poor intermittent recovery operation capability. By contrast, the introduction of zwitterionic species effectively resolved this issue. For further analysis, we also measured the in situ EIS of the symmetric cells at the beginning and end of plating/stripping during the first five cycles, as well as performed in situ DRT analysis of the symmetric cells. Detailed results are provided in Figs. S43 and S44. To further demonstrate the stability of aggregates in the Zn(OTf)_2_/C_10_ electrolyte, we measured the DLS spectra of the electrolyte after cycling at 5 mA cm^−2^ and found that the peak corresponding to C_10_ self-assembly still persists (Fig. S45). This indicates that the aggregates in the bulk electrolyte remain stable under electrochemical cycling.

The plating/stripping of Zn^2+^ on heterogeneous substrates was further assessed by evaluating the Coulombic efficiency (CE) in the asymmetric Zn||Cu cells (Fig. 5g). At 1 mA cm^−2^ and 1 mAh cm^−2^, Zn(OTf)_2_/C_10_ maintained stable cycling for 800 cycles (or 1600 h), achieving a CE of around 99.9%. In contrast, Zn(OTf)_2_ failed after 35 cycles due to dendrite formation and side reactions. Although Zn(OTf)_2_/C_2_ improved cycling to approximately 300 cycles, this value was much lower than that of Zn(OTf)_2_/C_10_. We further supplemented our study with the rate performance of Zn||Cu asymmetric cells (Fig. S46). It can be seen that, at a current density of 3 mA cm⁻^2^, the blank electrolyte already fails. In contrast, the addition of C_10_ enables the cells to exhibit higher and more stable Coulombic efficiency (CE) across all tested current densities. Moreover, we employed Ti as an inert electrode to test the rate performance of asymmetric cells (Fig. S47). Due to the poorer electrical conductivity of Ti and its unfavorable lattice matching with Zn, the blank electrolyte fails within just three cycles. In contrast, the addition of C_10_ maintains higher and more stable CE at all current densities, whereas C_2_ shows noticeable fluctuations in CE. These results collectively demonstrate that C_10_ can effectively enhance the performance of asymmetric cells and exhibits broad applicability. As shown in the cyclic voltammetry (CV) curves of asymmetric Zn||Cu cells (Fig. S48), the overpotential during the first reduction process slightly increased upon the addition of C_10_, which is consistent with the observations in symmetric cells. This phenomenon led to smaller Zn nuclei formation, thereby enhancing the stability of the deposition [60]. At the same time, the areas of both the reduction and oxidation peaks for Zn(OTf)_2_/C_10_ were larger than those for Zn(OTf)_2_ and Zn(OTf)_2_/C_2_, which means that a greater amount of Zn was deposited and stripped. These results collectively demonstrate that C_10_ in the electrolyte greatly improves the fast, selective, and uniform stable transport kinetics of Zn^2+^, suppresses HER and corrosion, and eliminates dendrite formation, leading to more reversible Zn plating/stripping.

**Fig. 6:**
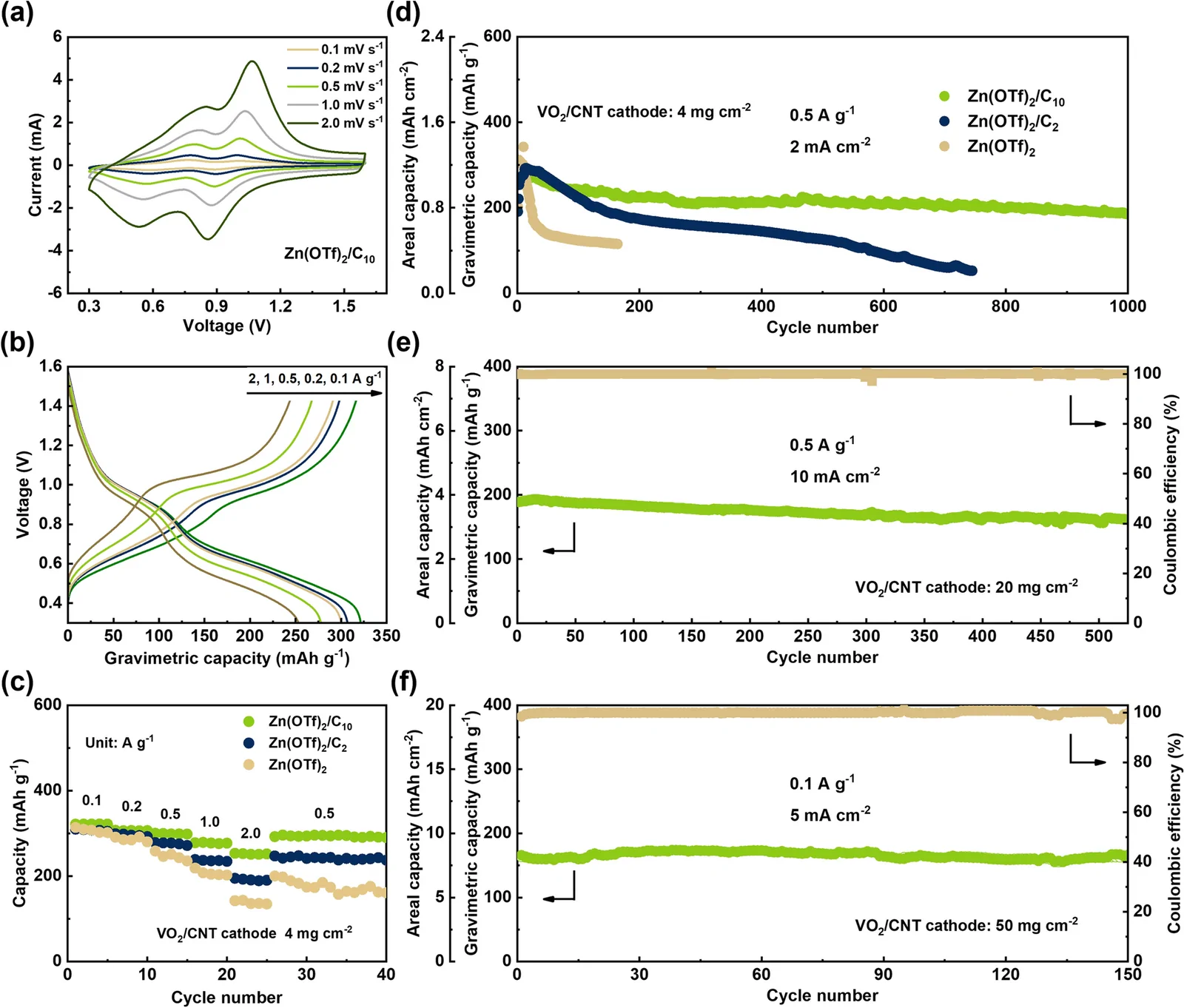
Electrochemical properties of the full cells. **a** CV curves of Zn||VO_2_/CNT in Zn(OTf)_2_/C_10_ using different scan rates. **b** Galvanostatic charge–discharge profiles of Zn||VO_2_/CNT in Zn(OTf)_2_/C_10_ at various current densities. **c** Rate performances of Zn||VO_2_/CNT in different electrolytes at 0.1, 0.2, 0.5, 1, and 2 A g^–1^. **d** Cycling performances of Zn||VO_2_/CNT in different electrolytes at 0.5 A g^–1^. **e** Cycling performances of Zn||VO_2_/CNT in Zn(OTf)_2_/C_10_ at 0.5 A g^–1^ (10 mA cm^−2^) with high mass loading of 20 mg cm^−2^. **f** Cycling performances of Zn||VO_2_/CNT in Zn(OTf)_2_/C_10_ at 0.1 A g^–1^ (5 mA cm^−2^) with ultrahigh mass loading of 50 mg cm.^−2^

For the practical application of the self-assembled C_10_-containing electrolytes, Zn||VO_2_/CNT full cells were assembled. The successful synthesis of the VO_2_/CNT was confirmed through XRD analysis (JCPDS No. 00–031-1438) and SEM characterization (Figs. S49 and S50). During the first charging cycle, an asymmetric oxidation peak of CV curve appears at ~ 1.6 V, which is characteristic of the phase transformation from VO_2_ to the hydrated V_2_O_5_ (Fig. S51) [61]. As shown in Fig. 6a, the Zn||VO_2_/CNT cells in Zn(OTf)_2_/C_10_ clearly showed two reduction peaks at 0.9 and 0.6 V, as well as two oxidation peaks at 0.75 and 1.0 V, within cutoff voltage from 0.3 to 1.6 V. These peaks correspond to the reduction of V^5+^/V^4+^ and the oxidation of V^3+^/V^4+^, respectively [62]. The GCD curves further supported these findings in Fig. 6b, showing two sloped plateaus for the cells in Zn(OTf)_2_/C_10_ within the cutoff voltage range from 0.3 to 1.6 V, in a good agreement with CV curves. These plateau features were preserved increasing the current densities from 0.1 to 2.0 A g^−1^. The specific capacities of full cells with the cathode mass loading of 4 mg cm^−2^ were measured in three electrolytes at the current densities from 0.1 to 2.0 A g^−1^ (Fig. 6c). The full cell in Zn(OTf)_2_/C_10_ achieved the highest discharge capacity of 322 mAh g^−1^ at 0.1 A g^−1^, surpassing the 307 and 308 mAh g^−1^ in Zn(OTf)_2_ and Zn(OTf)_2_/C_2_, respectively. These capacity gaps between Zn(OTf)_2_/C_10_ and others were enlarged as the current densities increased due to the fast charge transfer kinetics by C_10_. Even at the high current density of 2 A g^−1^, the specific capacity of the cell in Zn(OTf)_2_/C_10_ was preserved up to 249 mAh g^−1^, or 77% retention of capacity rated at 0.1 A g^−1^, much greater than 133 mAh g^−1^ (43%) and 190 mAh g^−1^ (62%) in Zn(OTf)_2_ and Zn(OTf)_2_/C_2_, respectively, indicating the high rate capability. Upon reverting to a current density of 0.5 A g^−1^, the full cells in Zn(OTf)_2_/C_10_ exhibited smooth cycling returning to the original discharge capacity, while the Zn(OTf)_2_ cells showed noticeable fluctuations, which further supports the superiority of the self-assembled C_10_. The stable and reversible kinetics in Zn(OTf)_2_/C_10_ was further investigated demonstrating the increase in the capacitive contribution of the cathode from 67 to 93% as the scan rates increased from 0.1 to 2.0 mV s^−1^, respectively (Fig. S52). This indicates that C_10_ facilitates the charge storage kinetics, which in turn contributes to the enhanced rate performances [63, 64].

A long-term cyclic test was carried out at a current density of 0.5 A g^−1^ (or 2 mA cm^−2^) with the cathode mass loading of 4 mg cm^−2^ (Fig. 6d). The full cells in Zn(OTf)_2_/C_10_ delivered the high specific capacity of 185 mAh g⁻^1^ over 1000 cycles, much better than 51 mAh g^−1^ in Zn(OTf)_2_/C_2_ over 750 cycles. Furthermore, the full cells in Zn(OTf)_2_ exhibited rapid capacity decay, losing nearly half of its initial capacity within 35 cycles and short-circuiting after 166 cycles. For more practical applications, the full cells with a high mass loading of 20 mg cm^−2^ were tested at a high current density of 10 mA cm^−2^ (Fig. 6e). The Zn(OTf)_2_/C_10_ system allowed the Zn||VO_2_/CNT cells to achieve a very high capacity of 164 mAh g^−1^ (or 3.28 mAh cm^−2^) after 520 cycles, with a capacity retention rate of 86% compared to its initial capacity of 190 mAh g^−1^. Additionally, nearly 100% CE was observed. Even with the ultrahigh mass loading of 50 mg cm^−2^, the Zn||VO_2_/CNT full cells were tested in Zn(OTf)_2_/C_10_ (Fig. 6f). At a high areal current density of 5 mA cm^−2^, the full cells maintained a reversible capacity of 162 mAh g^−1^ (or 8.10 mAh cm^−2^) after 150 stable cycles, with no significant capacity degradation. Furthermore, our pouch cells successfully powered an electronic alarm clock, highlighting practical applications (Fig. S53). Importantly, our self-assembled C_10_ co-solutes not only offer exceptional value and potential for the commercialization of full cells, particularly in the context of recently published high-loading V-based ZMBs and other cathode systems, but also establish two unprecedented advances over previously reported zwitterionic additives. Specifically, we provide the first rigorous evidence of quasi-spherical aggregate formation by zwitterions and further demonstrate their unique ability to induce LHCEs, which were not realized in earlier studies. These distinctive solvation chemistries, together with robust SEI regulation, enable our full cells to deliver a record-high capacity of 8.10 mAh cm^−2^ at an ultrahigh active material loading of 50 mg cm^−2^, underscoring both their scientific significance and commercialization potential (Tables S4–S7).

Although this study mainly focuses on the Zn anode and electrolyte chemistry, we have nevertheless supplemented characterization on the cycled cathode to dispel any concerns about possible side reactions on the cathode side. To verify the stability of C_10_ on the cathode side, we selected the electrode charged to 1.6 V (after 20 cycles) for characterization. This is because if C_10_ were to undergo side reactions, they would most likely occur under high-potential (oxidizing) rather than low-potential (reducing) conditions. Moreover, 1.6 V is the cutoff charging voltage used in this work, at which both the electrolyte and additive are more susceptible to oxidation or decomposition. Thus, testing under this condition can better reflect the stability of C_10_ [65, 66]. We first examined the electrode surface morphology. The SEM images show highly consistent structures between the two samples, indicating that the introduction of C_10_ did not cause any noticeable structural damage or side reactions (Figs. S54 and S55). The cross-sectional SEM images further confirmed that the presence of C_10_ did not alter the morphology or structural continuity of the electrode layer (Figs. S56 and S57). Meanwhile, the corresponding EDX spectra (Fig. S58) showed that all elements were uniformly distributed in the Zn(OTf)_2_/C_10_ sample, without any signs of local enrichment or formation of new phases. According to the quantitative results, the content of N and S slightly increased, which can be attributed to the adsorption or residual presence of C_10_ molecules (Table S8). However, SEM and EDX alone cannot provide information on the chemical states; therefore, we further performed XPS analysis to examine possible changes in chemical bonding and interfacial composition (Fig. S59). From the C 1*s* spectra (Fig. S59a, b), no new peaks were observed after introducing C_10_, and the overall pattern remained identical to that of the pristine electrode, confirming that C_10_ does not undergo chemical decomposition or form new carbon-containing species at high potentials. The S 2*p* spectra (Fig. S59c) of both samples exhibited nearly identical profiles, in which the –SO_3_ signal originated from the retained Zn(OTf)_2_ salt and C_10_, suggesting that the electrolyte composition was well preserved. In the N 1*s* spectra (Fig. S59d), a distinct signal at ~ 402 eV appeared only in the C_10_-containing electrode, corresponding to the quaternary ammonium group of C_10_. The absence of additional peaks indicates that the nitrogen environment remained intact, ruling out possible cleavage or oxidation of C_10_ during charging. Furthermore, the V 2*p* spectra (Fig. S59e) of both electrodes displayed identical features, evidencing that C_10_ didn’t alter the valence state of vanadium [67]. Collectively, these results verify that C_10_ remains chemically stable even under the oxidative environment of 1.6 V. Its presence not only avoids parasitic reactions but also contributes to maintaining the structural integrity and compositional uniformity of the cathode interface.

## Conclusions

In this work, we have demonstrated that a self-assembled ordered nanostructure of zwitterionic C_10_ co-solutes induced LHCEs through the formation of both CIP and AGG for the increased local Zn^2+^ concentration. This ordered nanostructure of C_10_ was characterized as the quasi-spherical aggregates with a periodic length of 3.77 nm, which contributed to the formation of LHCEs, as verified by comprehensive analyses of synchronous SAXS, one-dimensional scattering, Guinier, PDDF, Porod techniques, and MD simulations. This unique solvated structure by the modified hydrogen-bonding networks was further characterized using FTIR, SERS, NMR, and computational analyses. Along withe the solvated structure, the EDL and SEI structrues were also modified facilitating interfacial charge transfer and suppressing parastitic reactions. Screening six types of zwitterionic compounds and varying concentrations, 1 molality of C_10_ was chosen as the optimum owing to the well-defined aggregated structures and high ionic conductivity and t_Zn_ values. The resulting Zn||Zn symmetric cells in Zn(OTf)_2_/C_10_ electrolytes achieved dendrite-free plating/stripping for over 2800 h at 1 mA cm^−2^ and 1 mAh cm^−2^ preserving long-term stability over 1200 h even at 5 mA cm^−2^ and 5 mAh cm^−2^ with a high DOD of 42.7%. Furthermore, the Zn||VO_2_/CNT full cells in Zn(OTf)_2_/C_10_ electrolytes delivered the remarkable reversible capacity of up to 185 mAh g^−1^ after 1000 cycles, delivering a high areal capacity of 3.28 mAh cm^−2^ after 520 cycles at a high mass loading of 20 mg cm^−2^. In particular, Zn(OTf)_2_/C_10_ electrolytes allowed full cells to achieve a record-high capacity of 8.10 mAh cm^−2^ at an ultrahigh mass loading of 50 mg cm^−2^ after 150 cycles. Therefore, this work underscores the importance of colloidal self-assembly on the control in the solvation environment and local interfacial structures by the nanoscale ordered structure as well as provides the rational design of advanced LHCEs for next-generation metal batteries.

## Supplementary Information

Below is the link to the electronic supplementary material.

Supplementary file1 (MP4 312 KB)

Supplementary file2 (MP4 344 KB)

Supplementary file3 (DOCX 9828 KB)
