# Supplementary material for: Self-Assembled Ordered Nanostructure of Zwitterionic Co-Solutes Induces Localized High-Concentration Electrolytes for Ultrastable and Efficient Zinc Metal Anodes
Source: Nanomicro Lett. 2026 Jan 4;18:194. doi: 10.1007/s40820-025-02040-4 (PMC12765768; doi:10.1007/s40820-025-02040-4)
Supplement: Supplementary file 3 — Supplementary file3 (DOCX 9828 KB) [file 40820_2025_2040_MOESM3_ESM.docx]

Supporting Information for

**Self-Assembled Ordered Nanostructure of Zwitterionic Co-Solutes Induces Localized High-Concentration Electrolytes for Ultrastable and Efficient Zinc Metal Anodes**

Shengyang Huang^1^, Zuyang Hu^1, 2^, Xin Wang Mo^1^, Yeonju Park^3, 4^, Jun Su Kim^1^, Gun Jang^1^, Dong Hyun Min^1^, Hao Fu^1^, Peixun Xiong^1^, Zhipeng Wen^2^, Young Mee Jung^3, 4^, Jaeyun Kim^1^, Hyunjoo Lee^5^, Chihyun Hwang^6^, Youngkwon Kim^6,^ *, Cheng Chao Li^2,^ *, Qingyun Dou^1, 7,^ * and Ho Seok Park^1, 8,^ *

^1^ School of Chemical Engineering, Sungkyunkwan University (SKKU), 2066 Seobu-ro, Jangan-gu, Suwon 16419, Republic of Korea

^2^ Guangdong Provincial Key Laboratory of Plant Resources Biorefinery, School of Chemical Engineering and Light Industry, Guangdong University of Technology, Guangzhou 510006, P. R. China

^3^ Department of Chemistry, Institute for Molecular Science and Fusion Technology, Kangwon National University, Chuncheon 24341, Republic of Korea

^4^ Kangwon Radiation Convergence Research Support Center, Kangwon National University, Chuncheon 24341, Republic of Korea

^5^ Clean Energy Center, Institute of Science and Technology (KIST), 5, Hwarang-ro 14-gil, Seongbuk-gu, Seoul 02792, Republic of Korea

^6^ Advanced Batteries Research Center, Korea Electronics Technology Institute (KETI), 25, Saenari-ro, Seongnam 13509, Republic of Korea

^7^ Department of Materials Science and Engineering, Sun Yat-Sen University, Guangzhou 510275, P. R. China

^8^ SKKU Institute of Energy Science and Technology (SIEST), Sungkyunkwan University, 2066, Seoburo, Jangan-gu, Suwon 440-746, Republic of Korea

*Corresponding authors. E-mail: douqy3@mail.sysu.edu.cn (Qingyun Dou), ykkim96@keti.re.kr (Youngkwon Kim), licc@gdut.edu.cn (Cheng Chao Li), phs0727@skku.edu (Ho Seok Park)

**S1 Experimental Section**

**S1.1 Electrolyte preparation**

The blank electrolyte of 2 molal (mol kg⁻¹) Zn(OTf)_2_ was formulated by dissolving zinc (II) trifluoromethanesulfonate (TCI, >98%) in deionized water. For the cell electrolyte preparation, varying concentrations of 3-(decyldimethylammonio)propanesulfonate inner salt (C_10_, Sigma-Aldrich, ≥98%), dimethylethylammoniumpropane sulfonate (C_2_, Sigma-Aldrich, ≥97%), 3-(N,N-dimethyloctylammonio)propanesulfonate inner salt (C_8_, Sigma-Aldrich, ≥98%), N-dodecyl-N,N-dimethyl-3-ammonio-1-propanesulfonate (C_12_, Sigma-Aldrich, >99%), 3-(N,N-dimethylmyristylammonio)propanesulfonate (C_14_, Sigma-Aldrich, ≥99%) and 3-(N,N-dimethylpalmitylammonio)propanesulfonate (C_16_, Sigma-Aldrich, ≥98%) were separately introduced into the blank electrolyte. The optimized concentration of co-solute is 1 molal.

**S1.2 Cathodes preparation**

The cathodes were prepared by blending 70 wt% of active materials, 20 wt% Ketjen Black (KB, Lion Specialty Chemicals Co., Ltd.), and 10 wt% polyvinylidene fluoride (PVDF, Sigma-Aldrich, average Mw ~180000) in 1-methyl-2-pyrrolidone (NMP, DAEJUNG). The resulting slurry was cast onto a titanium foil (MA-EN-CU-001001, Canrd Technology Co. Ltd.) and dried at 80 °C overnight, yielding a VO_2_/CNT loading of 4 mg cm^−2^ on each cathode. The high mass loading (20 mg cm^−2^) and ultrahigh mass loading (50 mg cm^−2^) free-standing cathodes were obtained by casting the slurry onto glass plates and then drying them.

**S1.3 Material characterizations**

For the electrolyte characterization, Fourier-transform infrared (FT-IR) spectra were recorded using two FT-IR 4700s (JASCO) employing attenuated total reflection (ATR) techniques. Raman spectra were collected with a Confocal Raman Spectrometer (NT-MDT) operating at a wavelength of 532 nm. SERS spectra were obtained with a LabRAM HR Evolution confocal Raman microscope (Horiba-Jobin-Yvon) equipped with an air-cooled, frequency-doubled Nd:YAG laser at an excitation wavelength of 532 nm. Samples for nuclear magnetic resonance (NMR) analysis were prepared by mixing electrolytes in deuterium oxide (D_2_O). The NMR spectra were recorded on a 700 MHz spectrometer (AVANCE Ⅲ 700, Bruker). For SAXS, the scattered intensity was recorded using a Mar165 CCD detector with 2048 × 2048 pixels and a pixel size of 79 μm. The distance between the sample and the detector was 1600 mm. The active area is Φ165 mm. UV-vis absorption spectra were obtained by a MULTISKAN GO (Thermo SCIENTIFIC). DLS spectra were collected using a Zetasizer Nano ZS90 (Malvern Panalytical). To assess the ionic conductivities of the electrolytes, a real-time conductivity (SevenMulti, Mettler-Toledo) was utilized. Contact angle measurements were conducted using a contact angle goniometer (SmartDrop_Plus, FEMTOBIOMED). Digital images captured on an Honor 10 (Huawei). The electron backscatter diffractions of the Zn anodes were obtained from a JSM-IT800 (JEOL). The morphologies of the Zn anodes were analyzed using scanning electron microscopy (SEM, JSM-7000F, JEOL). A 3D laser confocal scanning microscope (3D LCSM, OLS5100, Olympus) was employed for three-dimensional imaging and reconstruction of post-cycled Zn samples. X-ray diffraction (XRD) patterns were recorded with an X-ray diffractometer (PANalytical, Almelo) using Cu Kα radiation (λ = 0.154 nm). X-ray photoelectron spectroscopy (XPS, ESCALAB 250, Thermo-Scientific) was used for characterizing the surface components of the electrodes, with in-depth XPS spectra obtained through argon ion sputtering for varying durations (0 s, 300 s, 600 s, and 900 s). The binding energies in the XPS spectra were referenced against the adventitious C 1s peak at 284.6 eV. Time-of-flight secondary ion mass spectrometry (ToF-SIMS) measurements were conducted using a TOF-SIMS-5 (ION-TOF), with sputtering performed using a 1 keV O_2_ beam over a 150×150 µm^2^ area, while analysis was restricted to a 40×40 μm^2^ area using a pulsed 25 keV Bi^+^ primary beam.

**S1.4 Electrochemical measurements**

The electrochemical performances of all kinds of cells were evaluated using the WonATech WBCS3100L Battery Testing System, Maccor Series 4000 Battery Testing System and NEWARE Battery Testing System (CT-4008Tn-5V6A-S1). A comprehensive analysis of the cells' electrochemical properties, including cyclic voltammetry (CV), electrochemical impedance spectroscopy (EIS), linear sweep voltammetry (LSV), and chronoamperometry (CA), was carried out using a BioLogic VMP3 Multichannel Potentiostat. EIS measurements were performed over a wide frequency range of 10 Hz to 100 kHz to gain in-depth insights into the cell dynamics. The HER was assessed via LSV in electrolytes using NaOTf salt, using a sweep rate of 5 mV s^−1^. Chronoamperometry was employed to capture diffusion profiles under 150 mV. Corrosion behavior was examined in the three-electrode system with Tafel analysis. Zn foils were utilized as both the working and counter electrodes, while an Ag/AgCl electrode served as the reference.

The transference number of Zn^2+^ (t_Zn_) was obtained from the Zn||Zn symmetric cells. The procedure of CA was employed under 10 mV for 1200 s. The t_Zn_ value was calculated using Eq. 1:

$t_{Zn}= \frac{I_{s}\left( \Delta V-I_{0}R_{0} \right)}{I_{0}\left( \Delta V-I_{s}R_{s} \right)}$ (S1)

where *I_0_* and *Is* are the initial and steady-state currents, respectively. *ΔV* represents the applied potential. *R_0_* and *Rs* donate the initial and steady-state electrode resistances, respectively.

**S1.5 Calculation methods**

Density functional theory (DFT) calculations were conducted using the Gaussian (G09) program [S1]. The Becke’s three-parameter hybrid method with the Lee-Yang-Parr correlation functional (B3LYP) was employed [S2, S3]. Structural optimizations were conducted at the 6-311++G(d,p) level [S4], and single-point energy calculations were carried out using the def2-TZVP basis set [S5, S6]. Grimme’s dispersion correction with Becke-Johnson damping [DFT-D3(BJ)] [S7-S9] was applied throughout the calculations. Solvation effects were considered using the SMD universal solvation model [S10]. Frequency analyses were performed to confirm that all optimized ion-solvent complexes correspond to true minima on the potential energy surface. Electrostatic potential (ESP) analyses were conducted using Multiwfn [S11, S12] and visualized with VMD [S13] software. The binding energy (E_b_) between molecule/ion A and molecule/ion B was calculated according to Eq. 2:

$E_{b}= E_{complex}-E_{A}-E_{B}$ (S2)

Adsorption energy calculations were performed using the projector augmented wave (PAW) method as implemented in the Vienna Ab Initio Simulation Package (VASP) [S13]. The generalized gradient approximation (GGA) with the Perdew-Burke-Ernzerhof (PBE) functional was adopted to describe the exchange-correlation energy [S14, S15]. A plane-wave cutoff energy of 500 eV and a Γ-centered 3×3×1 k-point mesh were used in all calculations. Structural optimizations were carried out until the residual forces on all atoms were less than 0.03 eV Å⁻^1^, and the total energy convergence criterion was set to 1×10⁻^5^ eV. The adsorption energy (E_a_) between the Zn slab and the adsorbate was calculated using Eq. 3:

$E_{a}= E_{system}-E_{slab}-E_{Adsorbate}$ (S3)

Molecular dynamics (MD) were performed using the Large-scale Atomic/Molecular Massively Parallel Simulator (LAMMPS) based on the Optimized Potentials for Liquid Simulations All-Atom (OPLS-AA) force field [S16-S18]. The force field parameters for organic solvents were generated using the LigParGen web server, while the restrained electrostatic potential (RESP) atomic charges were derived from electrostatic potential (ESP) fitting using Multiwfn [S11, S12]. Initial atomic configurations were constructed using the Packmol package [S19, S20], and all final model boxes and solvation structures were visualized with VMD [S13]. Periodic boundary conditions were applied in all three dimensions. A cutoff distance of 10 Å was set for both van der Waals interactions and the particle-particle particle-mesh (PPPM) method for long-range Coulombic interactions. The time step was set to 1 fs. All electrolyte models were first equilibrated in the NVT ensemble using the Nosé-Hoover thermostat at 298 K for 5 ns [S21, S22]. Subsequently, a 20 ns NPT ensemble simulation was performed under a Nosé–Hoover thermostat and barostat to fully equilibrate the system at 298 K and 1 atm. A 15 ns production run was finally conducted under NPT conditions at 298 K and 1 atm, during which data for radial distribution function (RDF) and solvent shell analyses were collected.

S2 Results and Discussion


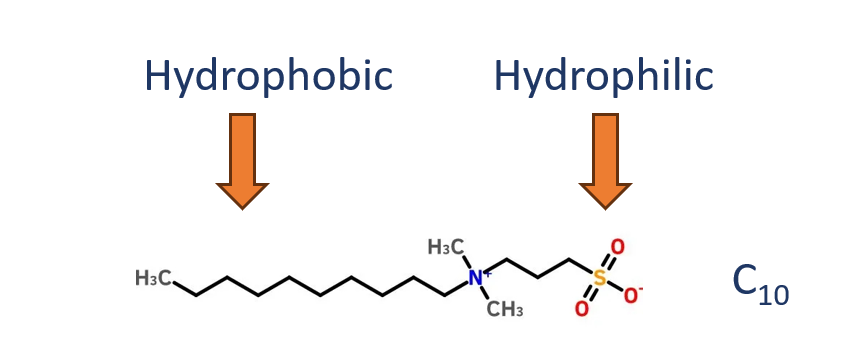


**Fig. S1** The amphiphilic characteristics of C_10_


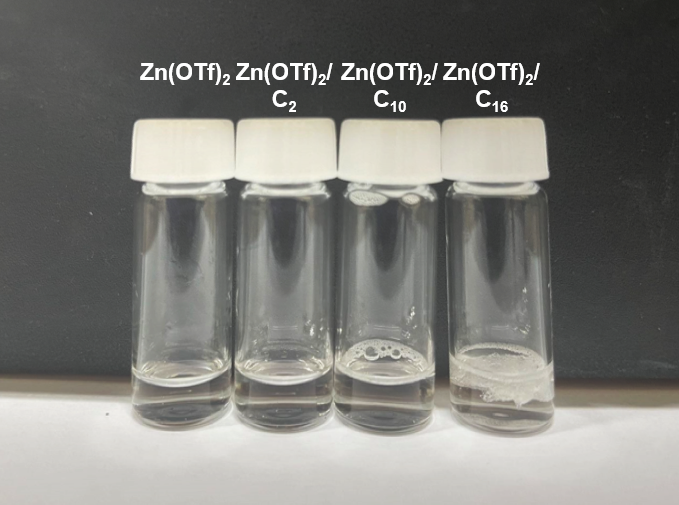


**Fig. S2** Digital images of different electrolytes


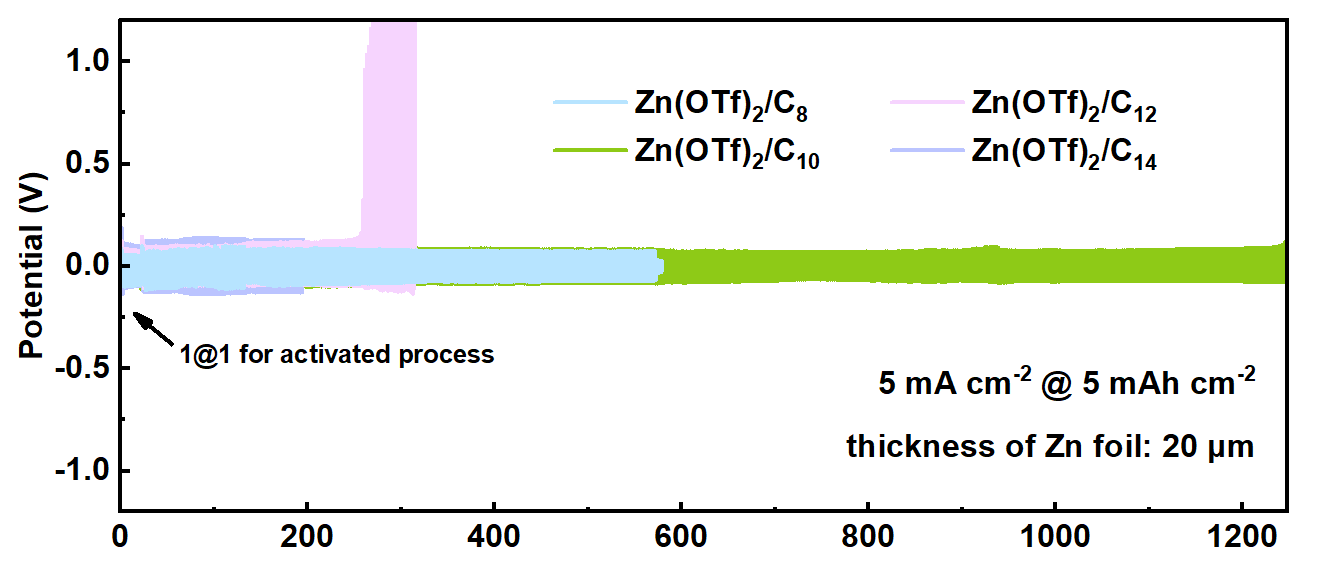


**Fig. S3** Cycling performances of Zn||Zn symmetric cells with C_8_, C_10_, C_12_, and C_14_ at 5 mA cm^−2^ for 5 mAh cm^−2^


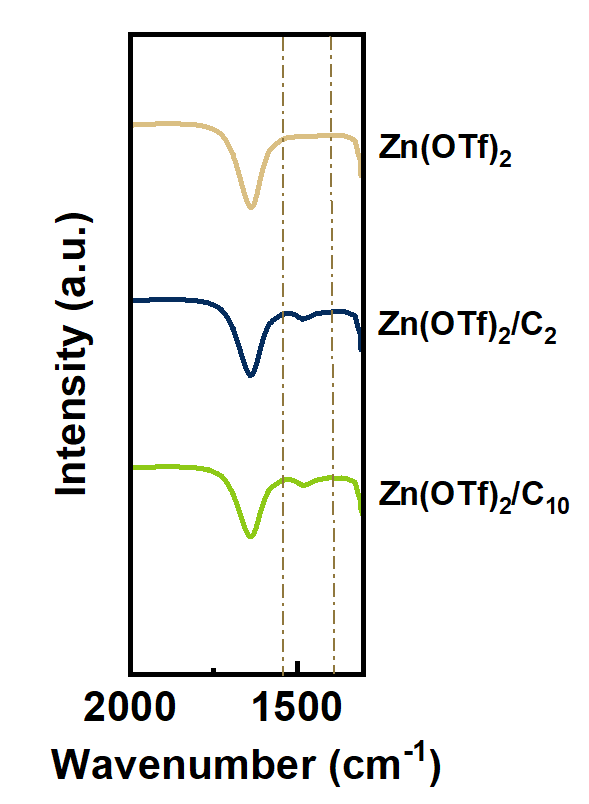


**Fig. S4** FT-IR spectra of different electrolytes at the range of 2000-1300 cm^−1^


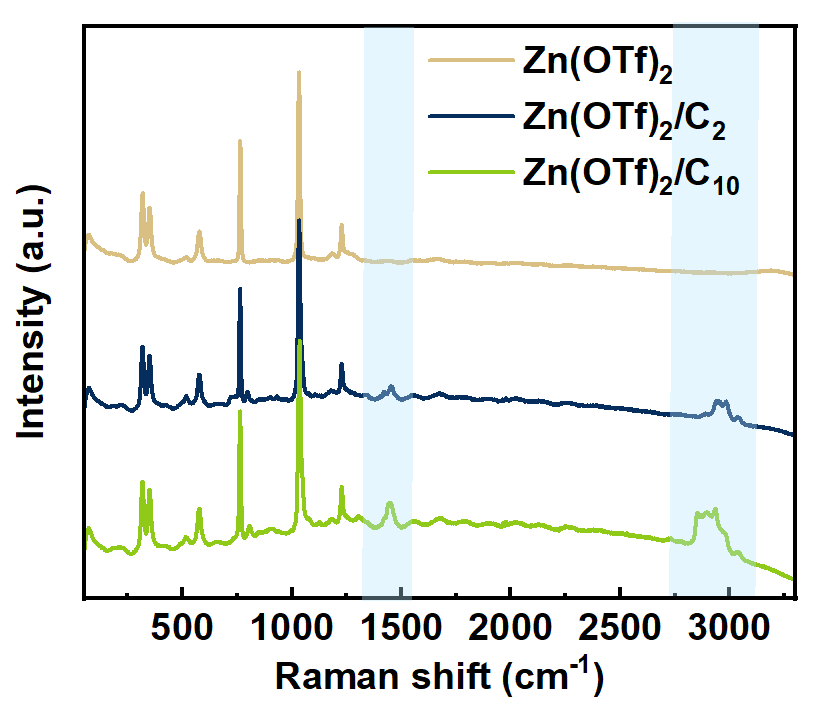


**Fig. S5** Raman spectra of different electrolytes


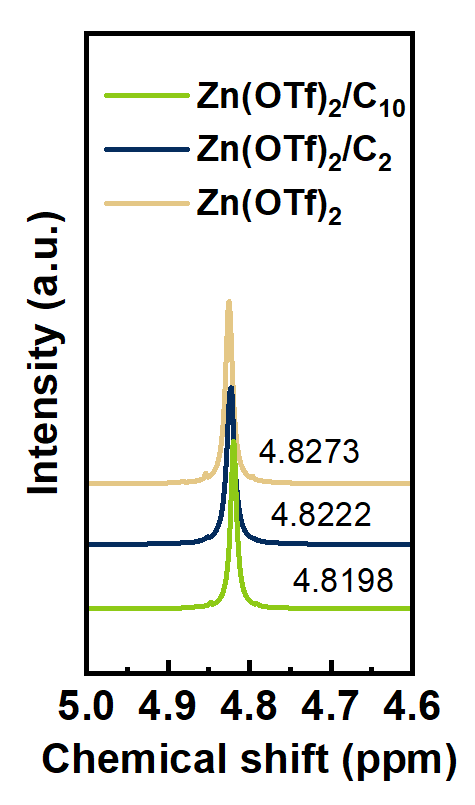


**Fig. S6** ^1^H NMR spectra of different electrolytes at the range of 5.0-4.6 ppm


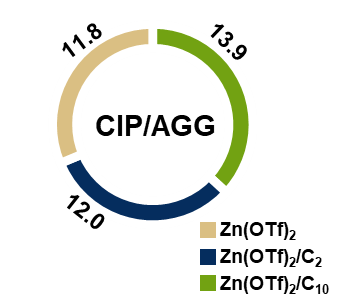


**Fig. S7** The ratios of CIP/AGG in all solvation structures in different electrolytes from MD simulations


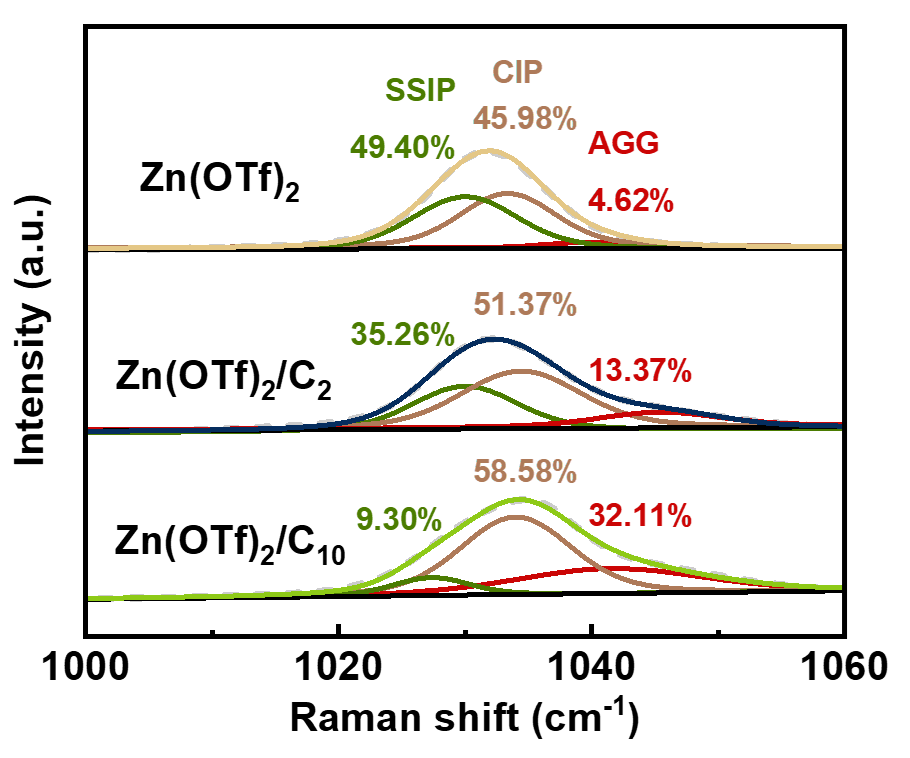


**Fig. S8** Raman spectra for SSIP/CIP


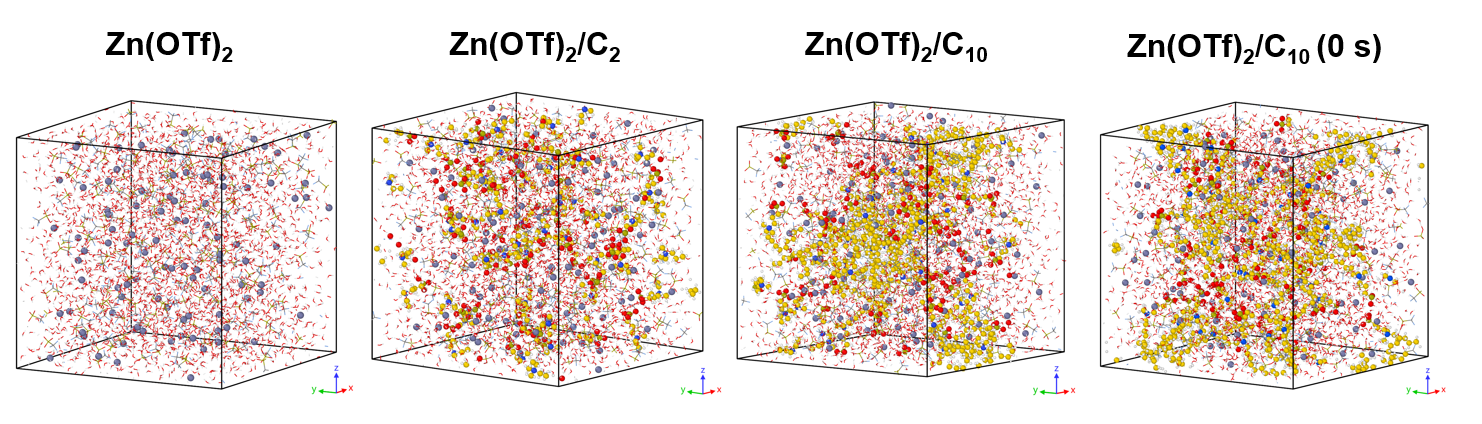


**Fig. S9** Snapshots of different electrolytes after 15 ns, and Zn(OTf)_2_/C_10_ at 0 s (3D images)


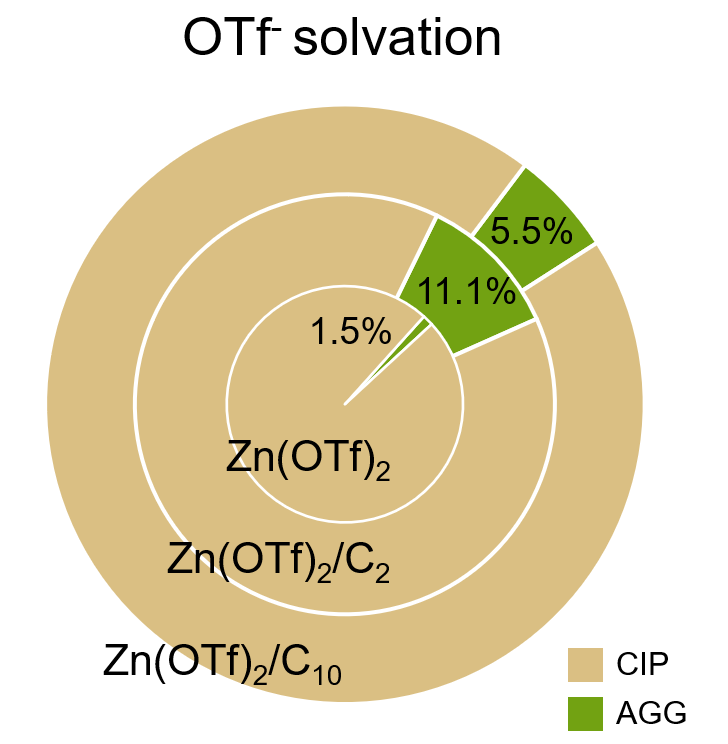


**Fig. S10** Statistical analysis of the proportion of AGG in CIP/AGG configurations


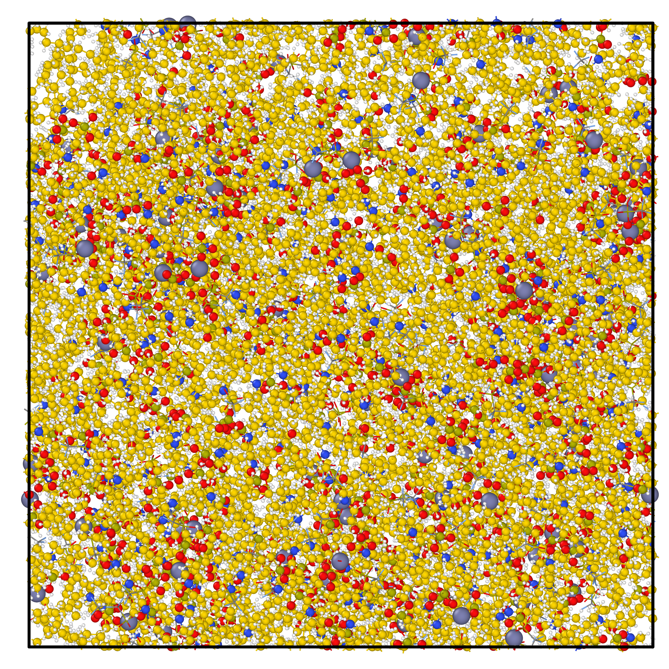


**Fig. S11** Snapshots of MD simulation about a water-free, high-C_10_-concentration system


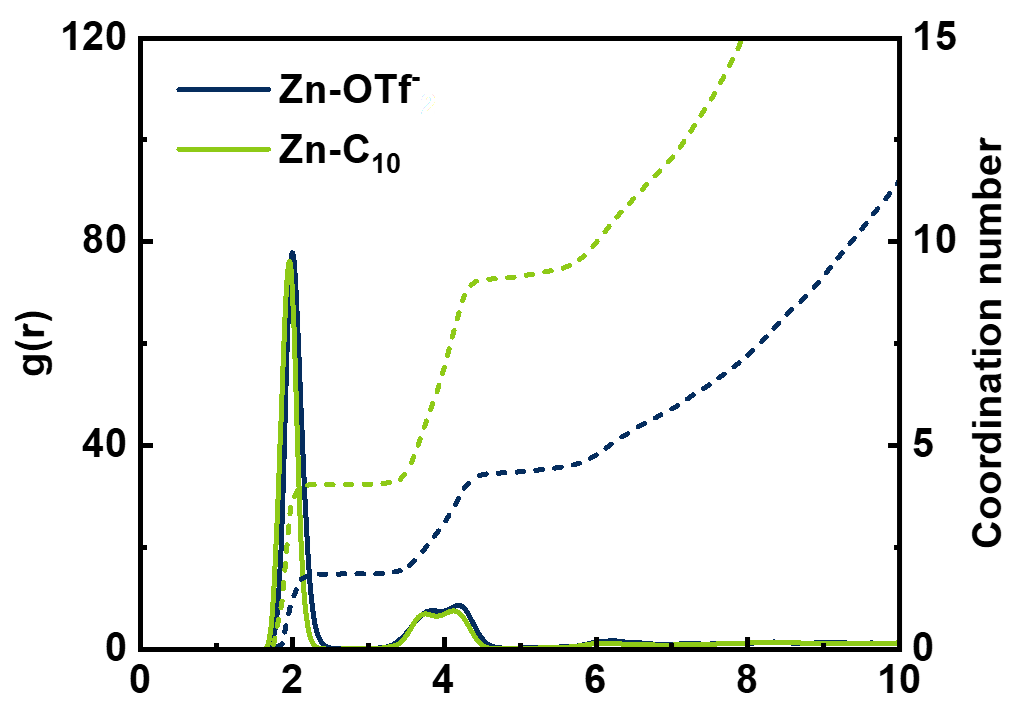


**Fig. S12** Radial distribution functions and corresponding coordination numbers of Zn^2+^–O in the water-free, high-C_10_-concentration system


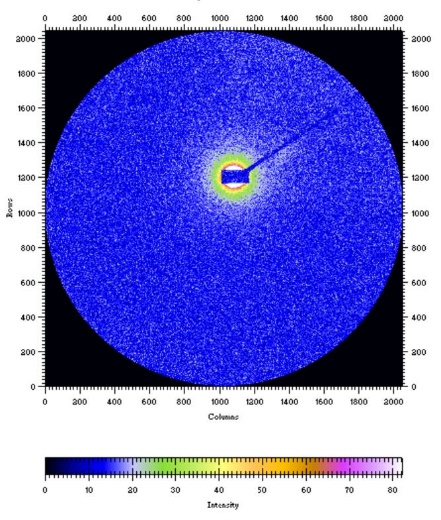


**Fig. S13** Two-dimensional SAXS image of Zn(OTf)_2_


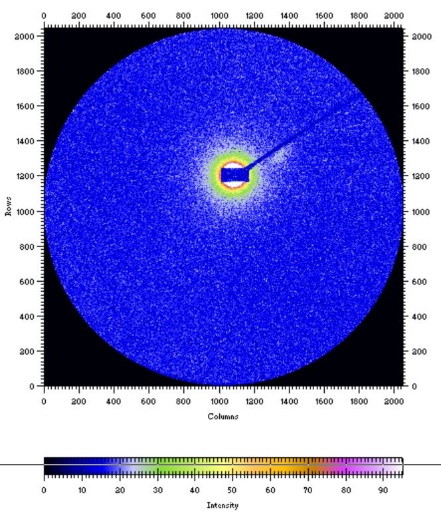


**Fig. S14** Two-dimensional SAXS image of Zn(OTf)_2_/C_2_


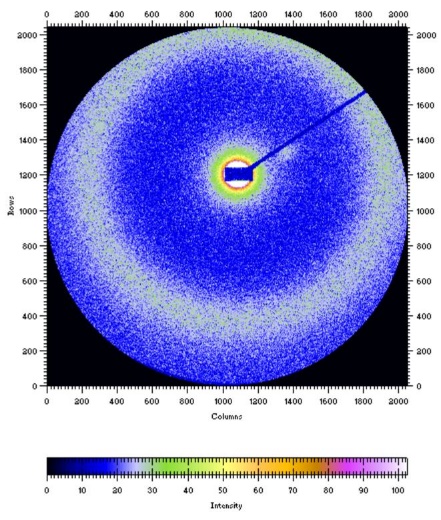


**Fig. S15** Two-dimensional SAXS image of Zn(OTf)_2_/C_10_


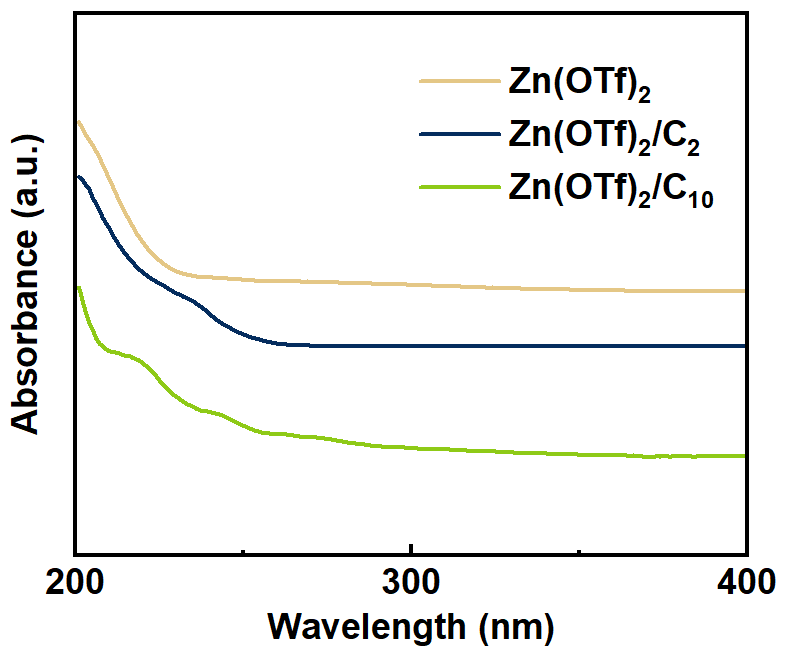


**Fig. S16** UV-vis spectra of different electrolytes


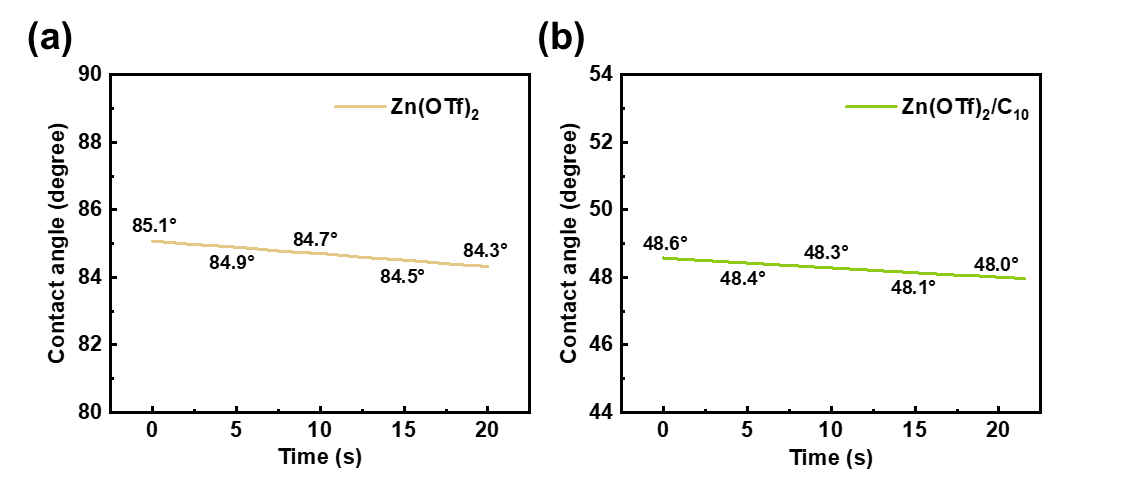


**Fig. S17** *In-situ* contact angle test of (**a**) Zn(OTf)_2_, and (**b**) Zn(OTf)_2_/C_10_


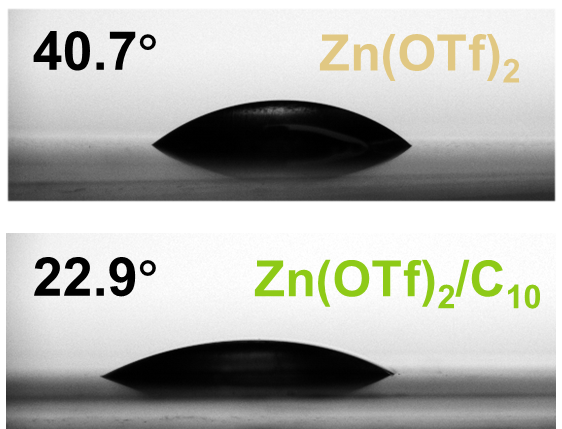


**Fig. S18** Contact angles of de-ionized water on Zn foil after immersion in different electrolytes


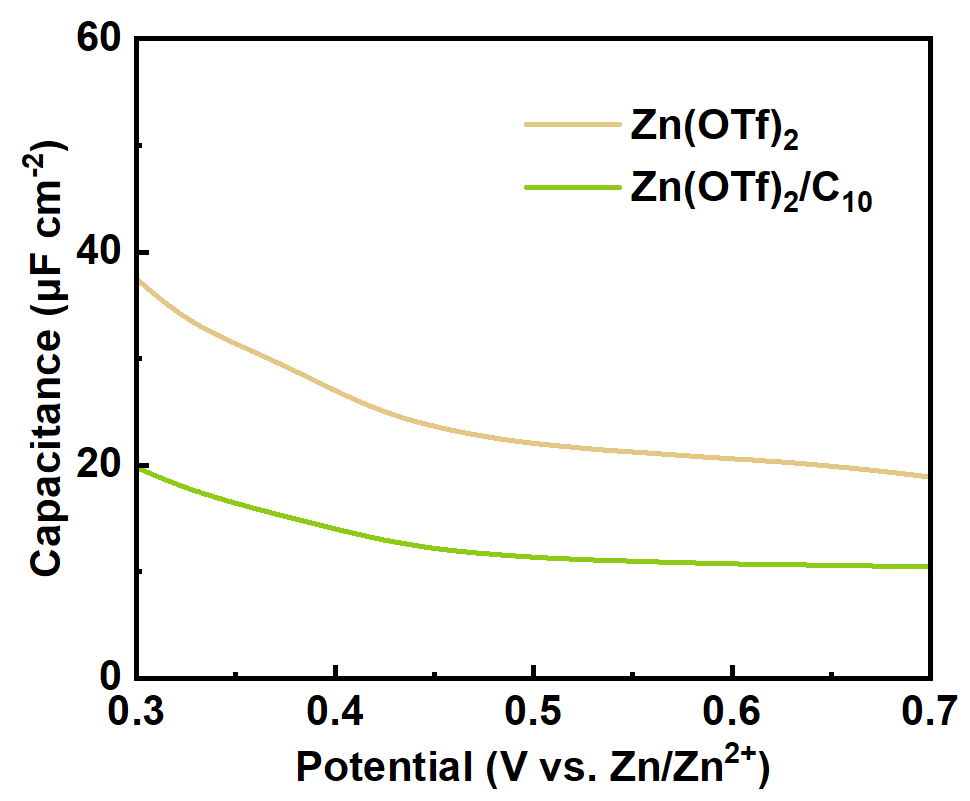


**Fig. S19** Capacitance-potential profiles of different electrolytes


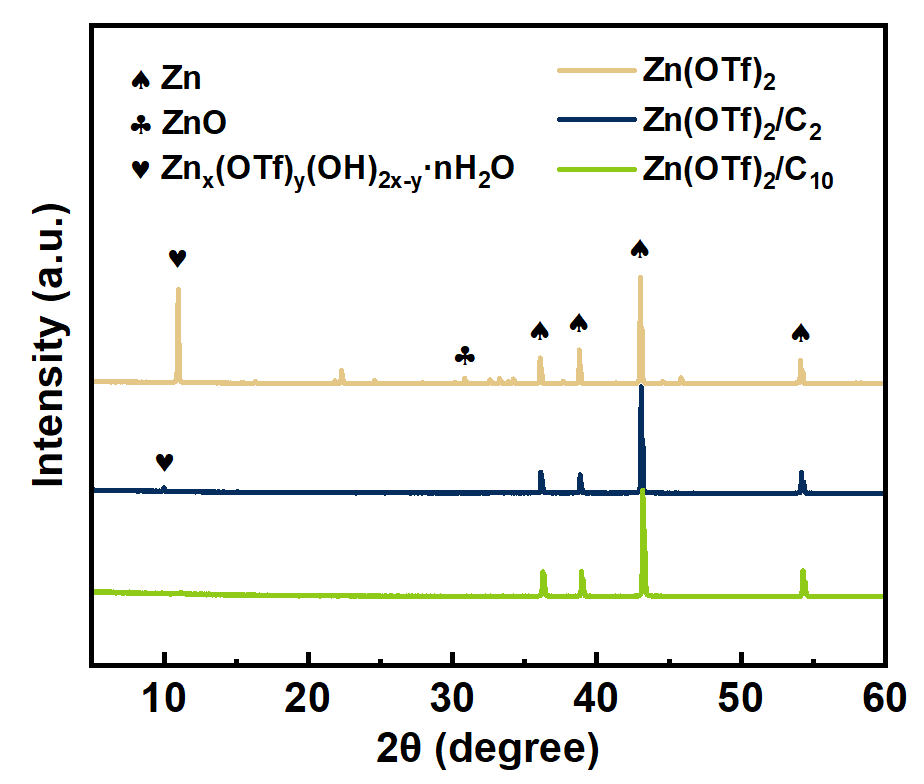


**Fig. S20** XRD images of Zn after immersing for 7 days in different electrolytes


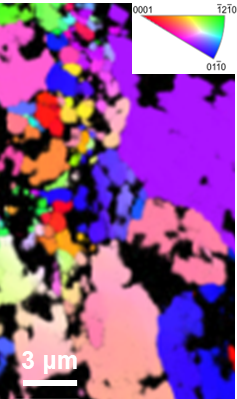


**Fig. S21** EBSD orientation map of Zn after plating/stripping for 20 times in Zn(OTf)_2_


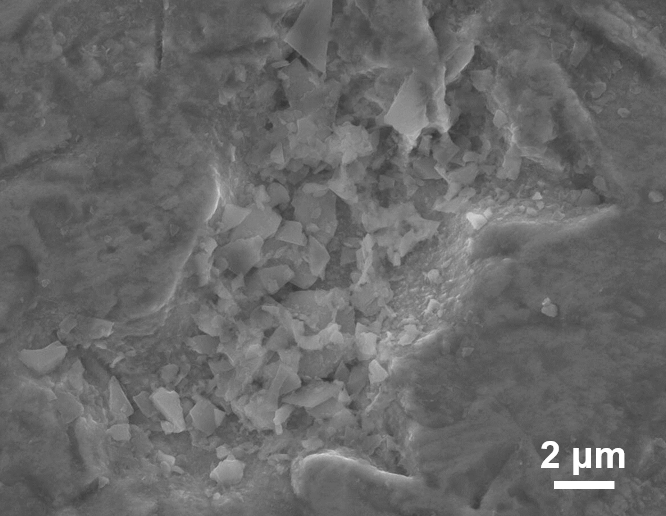


**Fig. S22** SEM image of Zn foil after immersing for 7 days in Zn(OTf)_2_


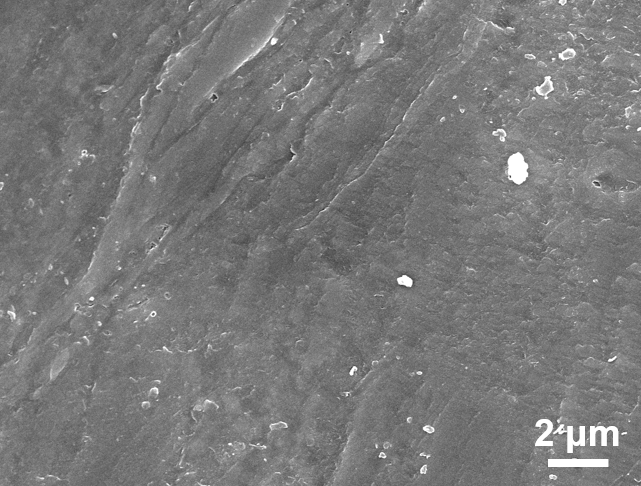


**Fig. S23** SEM image of Zn foil after immersing for 7 days in Zn(OTf)_2_/C_10_


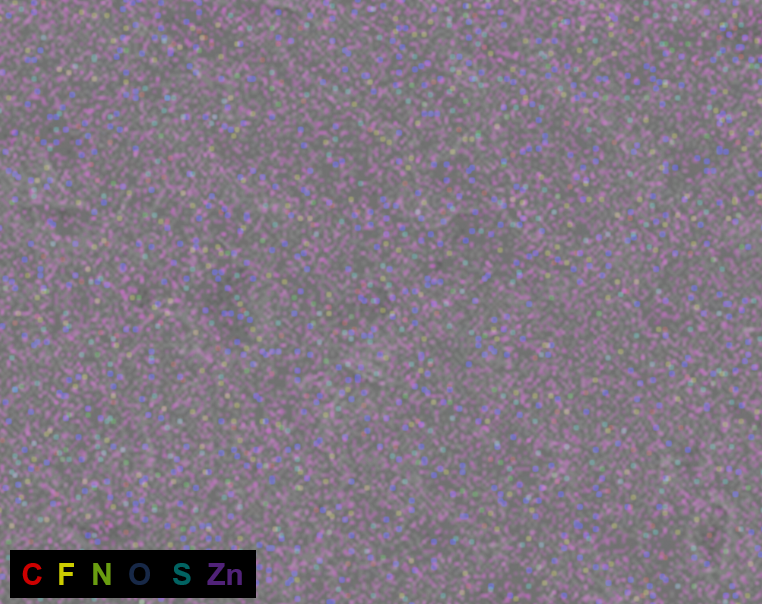


**Fig. S24** EDX mapping of Zn foil after immersing for 7 days in Zn(OTf)_2_/C_10_


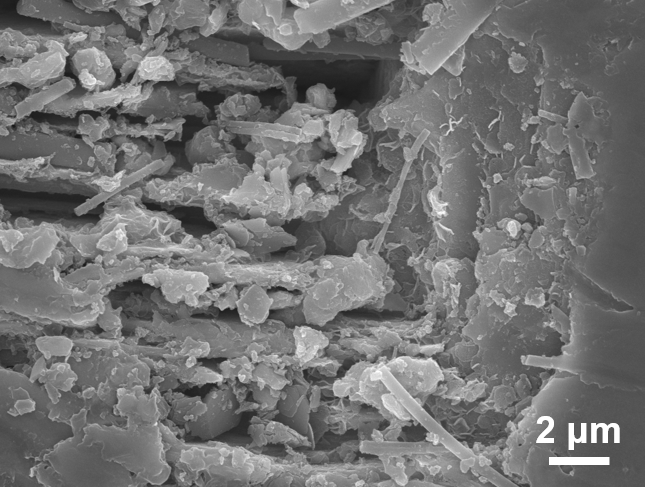


**Fig. S25** SEM image of Zn after plating/stripping for 20 times in Zn(OTf)_2_


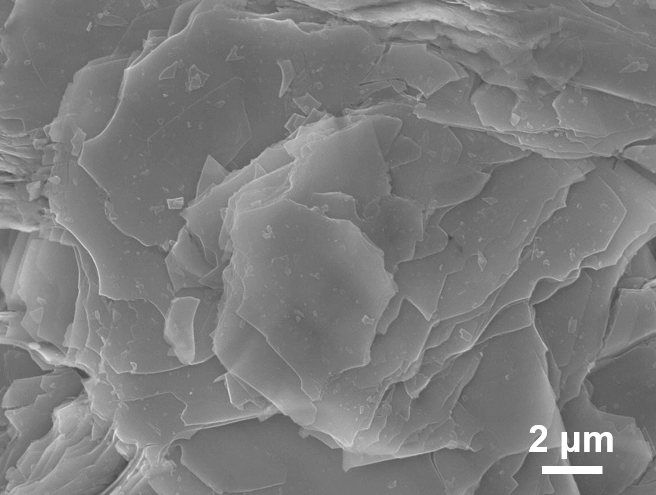


**Fig. S26** SEM image of Zn foil after immersing for 7 days in Zn(OTf)_2_/C_2_


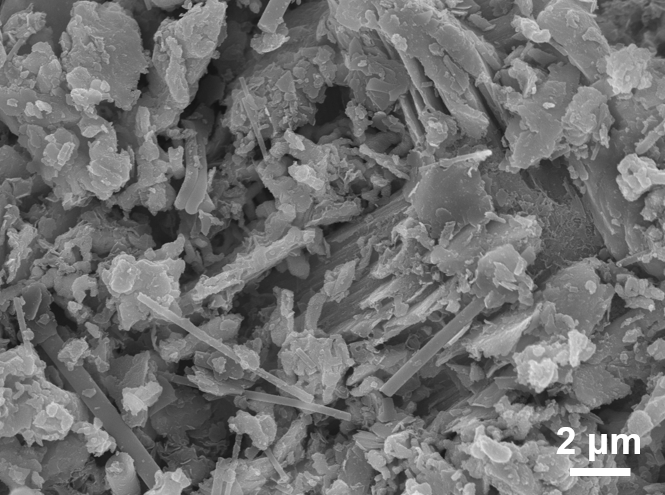


**Fig. S27** SEM image of Zn after plating/stripping for 20 times in Zn(OTf)_2_/C_2_


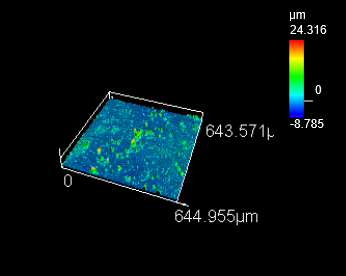


**Fig. S28** LSCM image of Zn after plating/stripping for 20 times in Zn(OTf)_2_


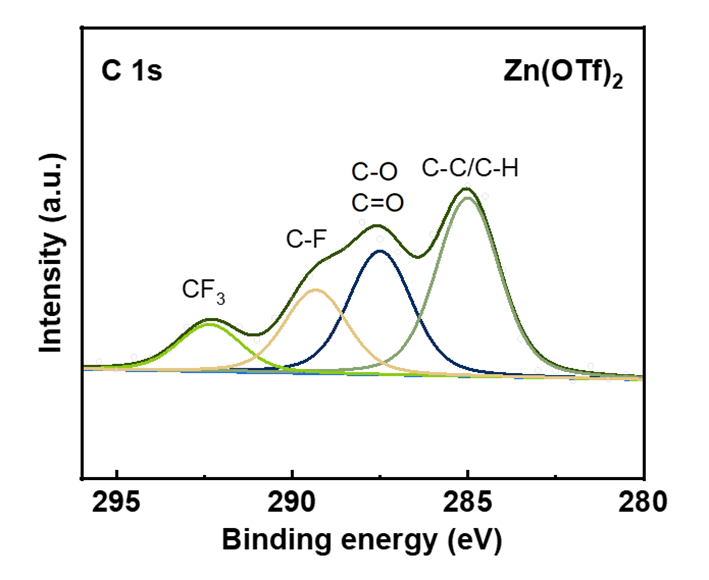


**Fig. S29** XPS C 1s spectra of Zn after plating/stripping for 20 times in Zn(OTf)_2_


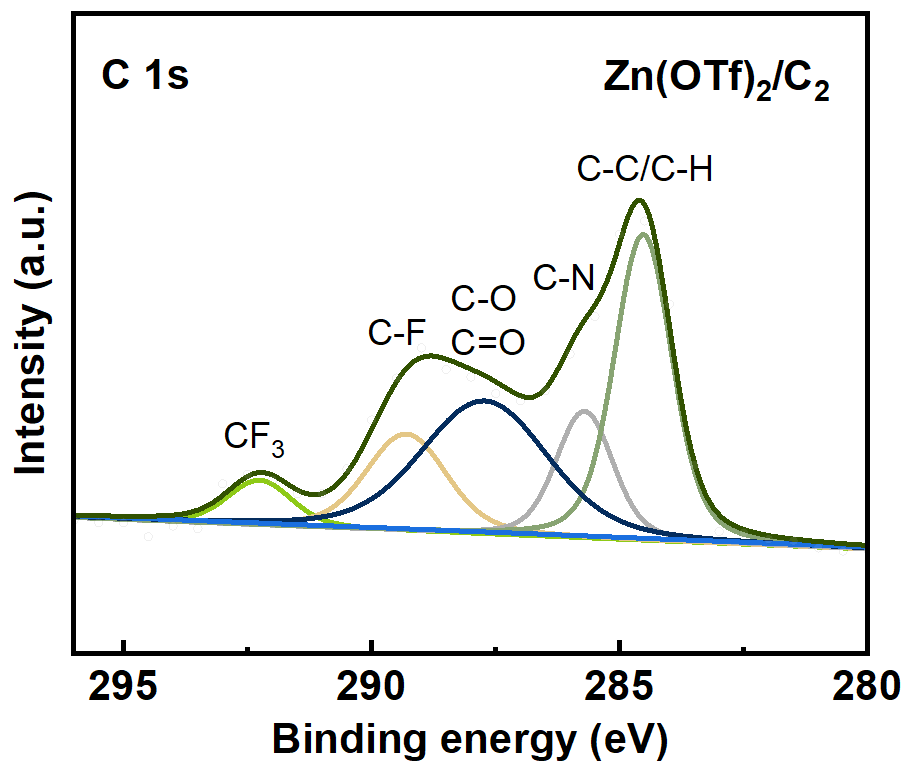


**Fig. S30** XPS C 1s spectra of Zn after plating/stripping for 20 times in Zn(OTf)_2_/C_2_


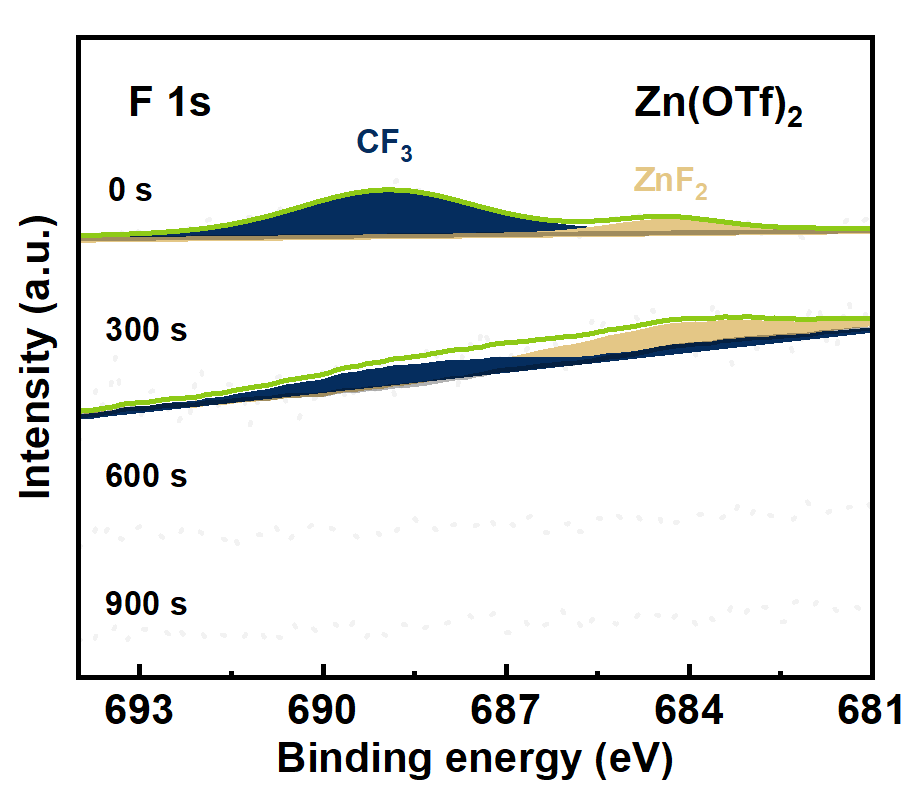


**Fig. S31** In-depth XPS F 1s spectra of Zn after plating/stripping for 20 times in Zn(OTf)_2_


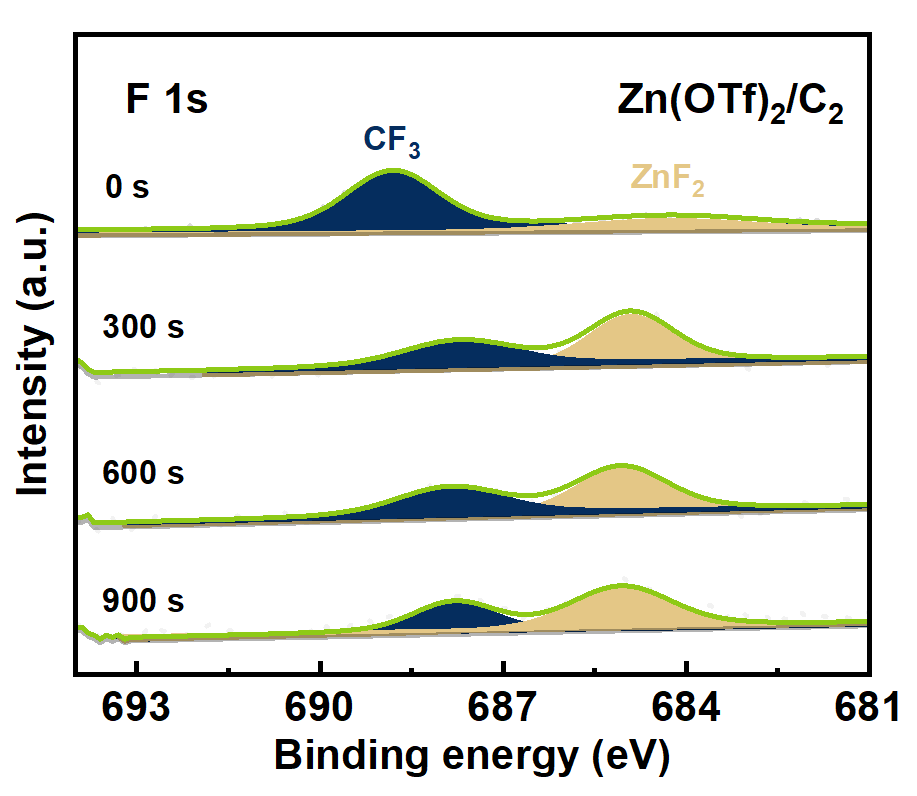


**Fig. S32** In-depth XPS F 1s spectra of Zn after plating/stripping for 20 times in Zn(OTf)_2_/C_2_


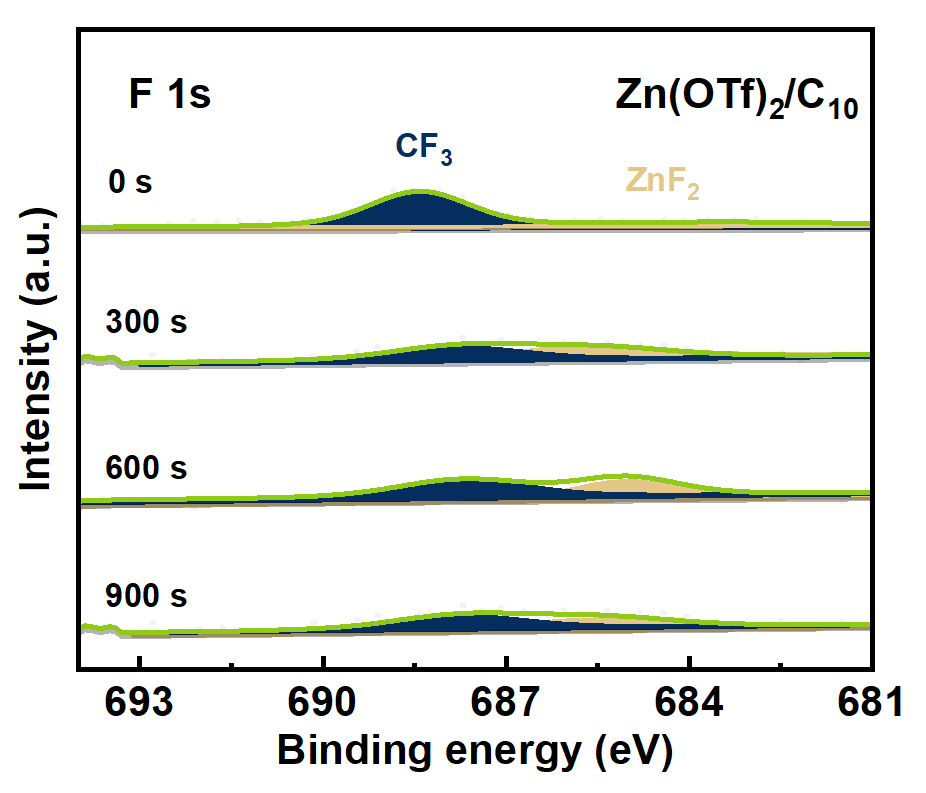


**Fig. S33** In-depth XPS F 1s spectra of Zn after plating/stripping for 20 times in Zn(OTf)_2_/C_10_


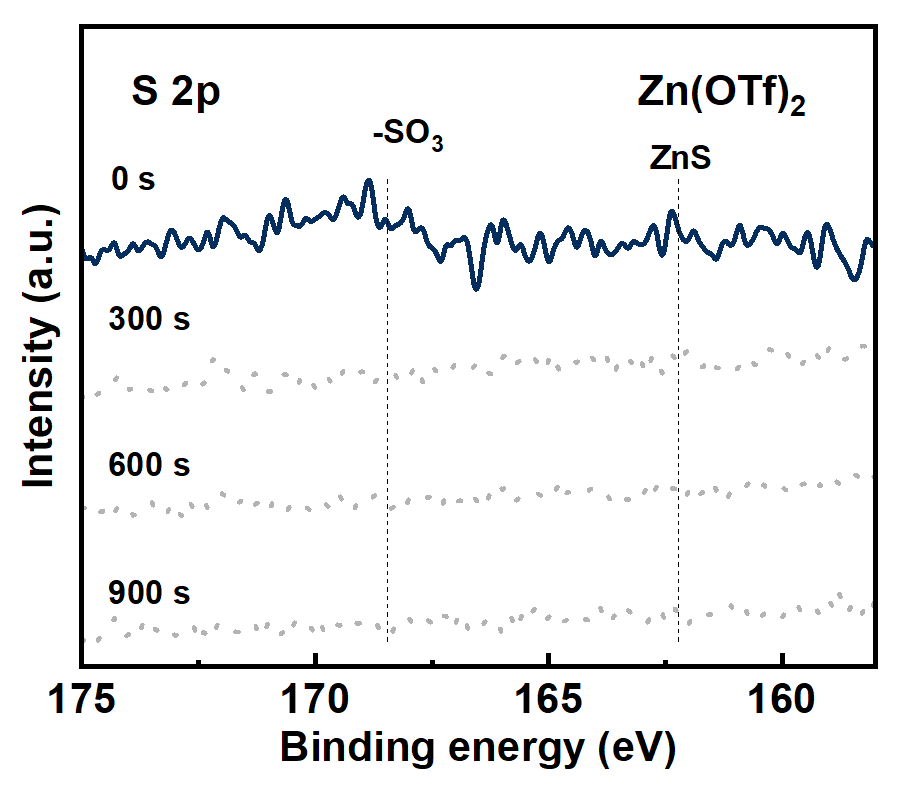


**Fig. S34** In-depth XPS S 2p spectra of Zn after plating/stripping for 20 times in Zn(OTf)_2_


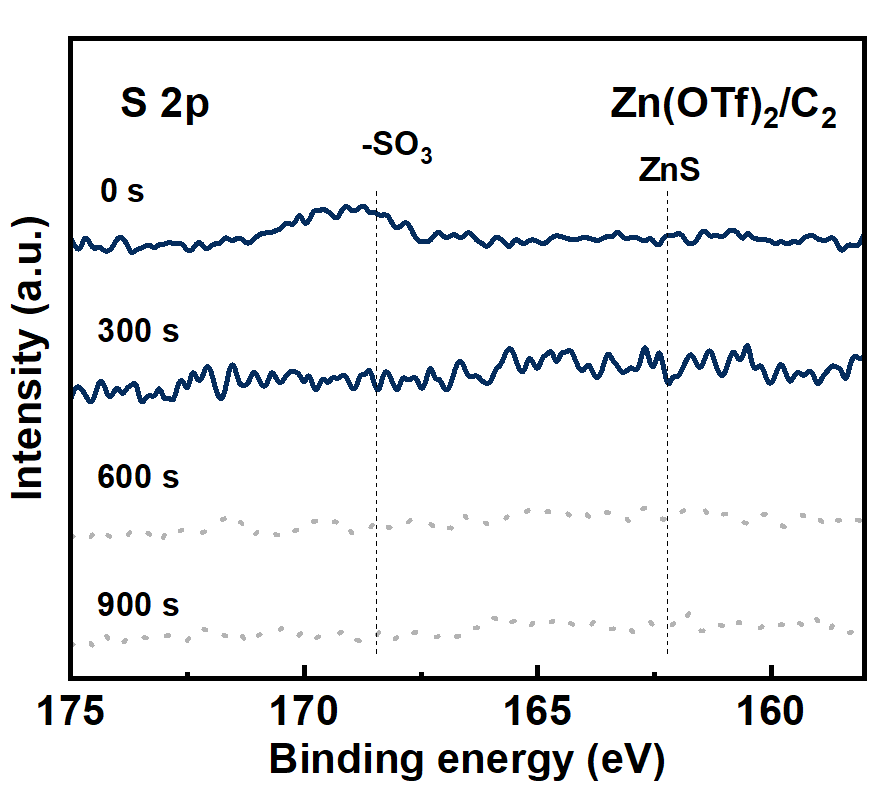


**Fig. S35** In-depth XPS S 2p spectra of Zn after plating/stripping for 20 times in Zn(OTf)_2_/C_2_


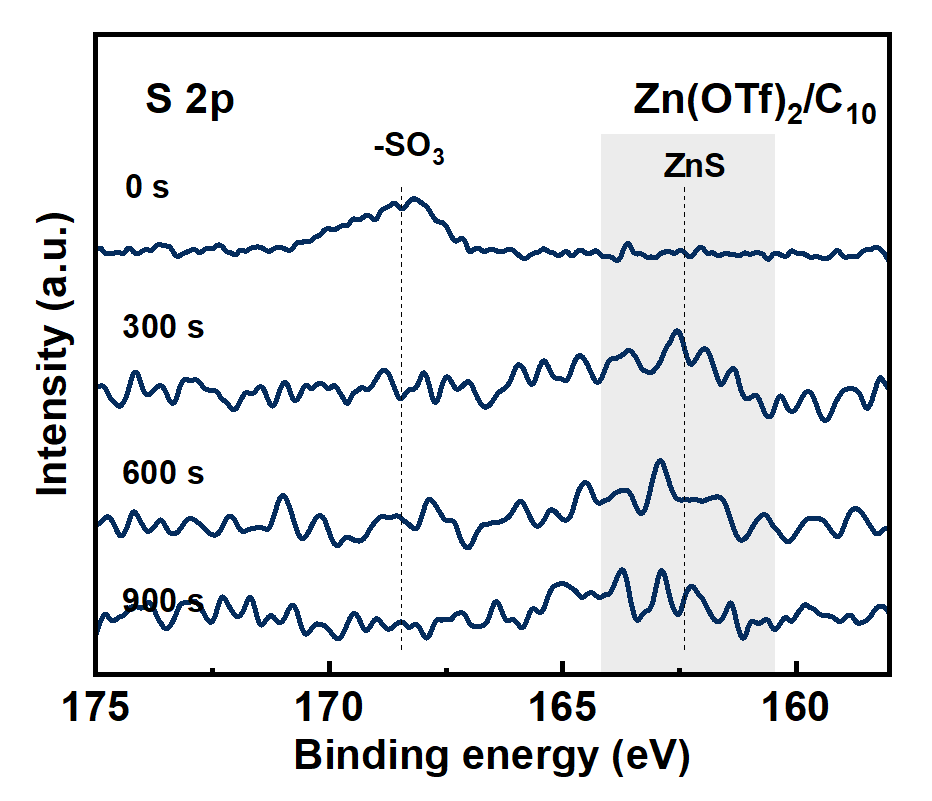


**Fig. S36** In-depth XPS S 2p spectra of Zn after plating/stripping for 20 times in Zn(OTf)_2_/C_10_


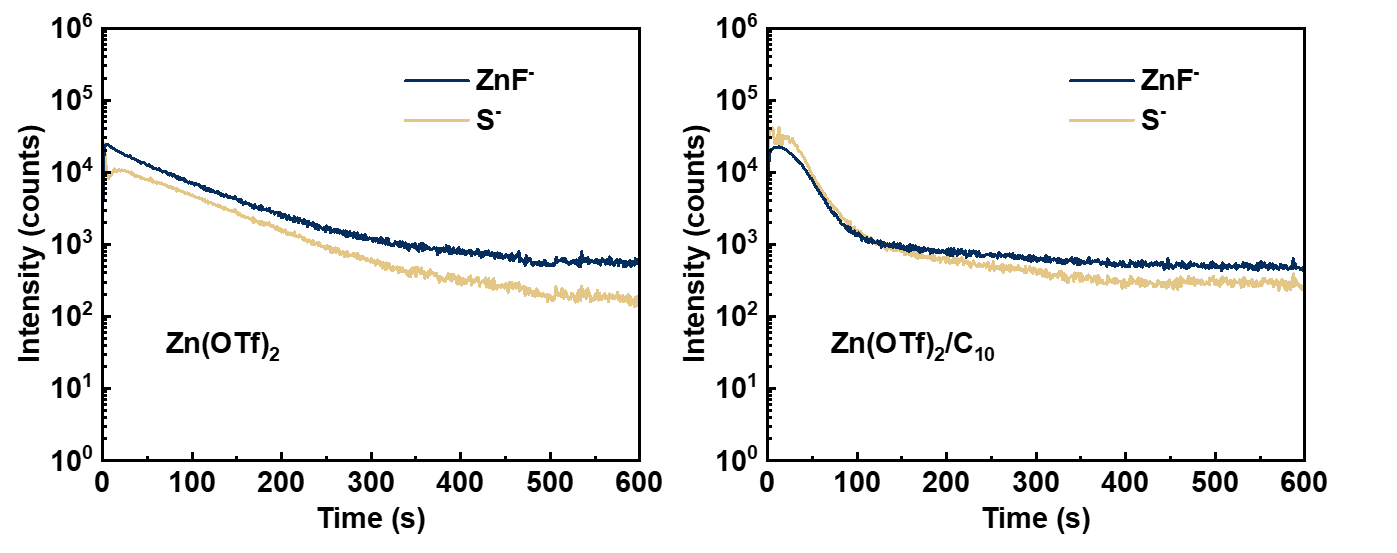


**Fig. S37** 2D ToF-SIMS spectra of Zn after plating/stripping for 20 times in Zn(OTf)_2_ and Zn(OTf)_2_/C_10_


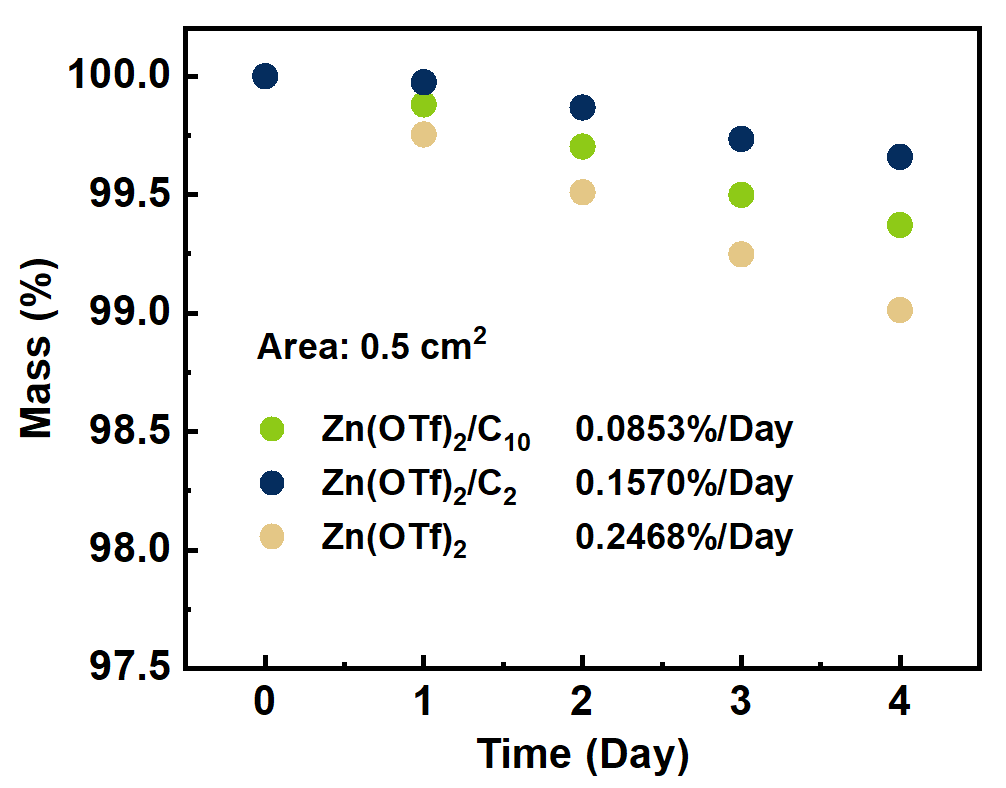


**Fig. S38** Mass change of Zn immersed in different electrolytes

To prevent the influence of basic byproducts, the samples were rinsed with 5% citric acid for 5 min before each mass measurement. The Zn(OTf)_2_/C_10_ shows the lowest mass change (0.0853%/Day) compared to The Zn(OTf)_2_ (0.2468%/Day) and Zn(OTf)_2_/C_2_ (0.1570%/Day), further confirming the corrosion-inhibiting effect of C_10_.


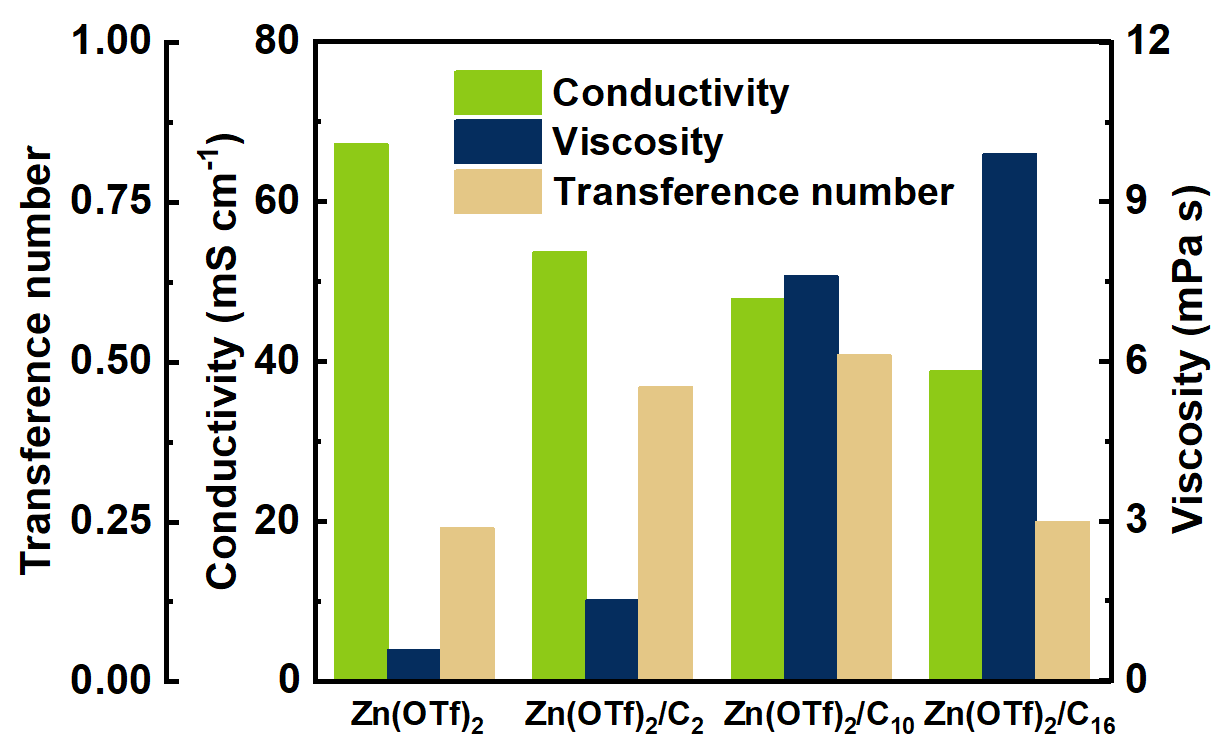


**Fig. S39** Conductivity, viscosity, and transference number of different electrolytes. Zn(OTf)_2_/C_16_ was tested under 40 °C


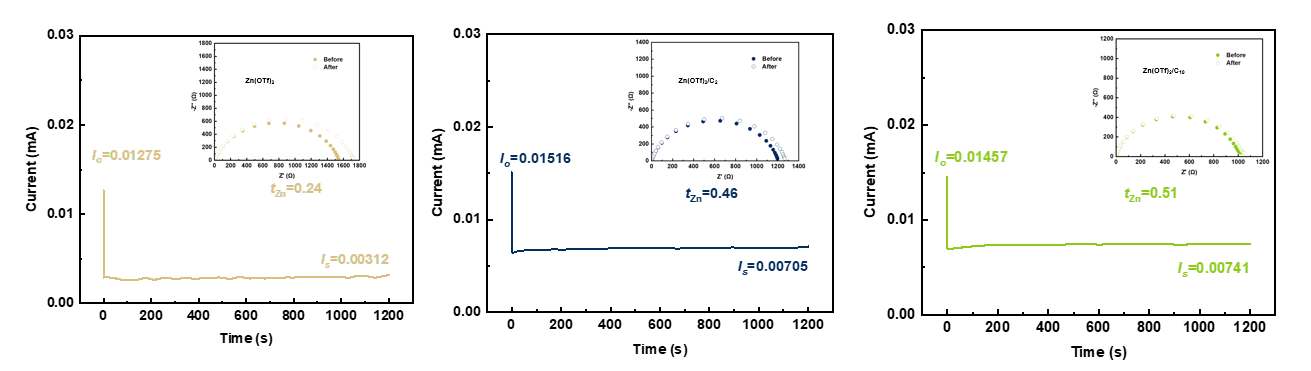


**Fig. S40** Transference number of different electrolytes


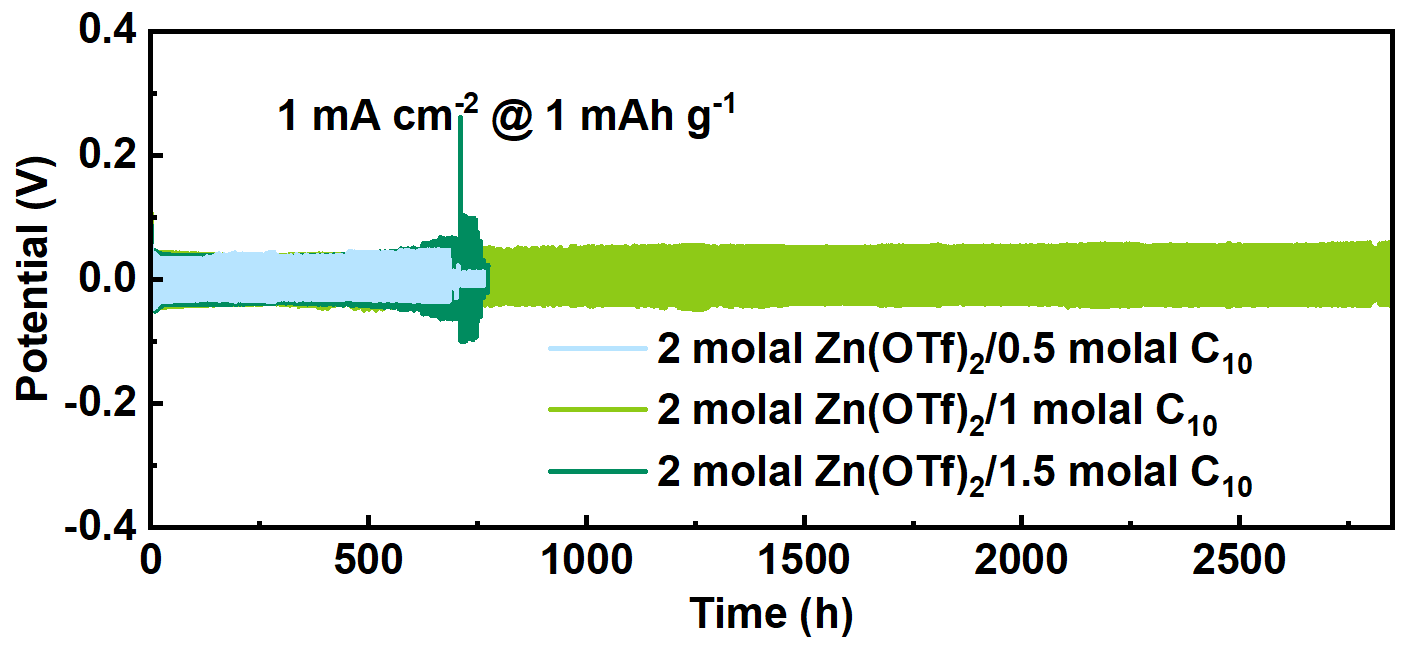


**Fig. S41** Cycling performances of Zn||Zn symmetric cells with different concentrations of C_10_ at 1 mA cm^−2^ for 1 mAh cm^−2^


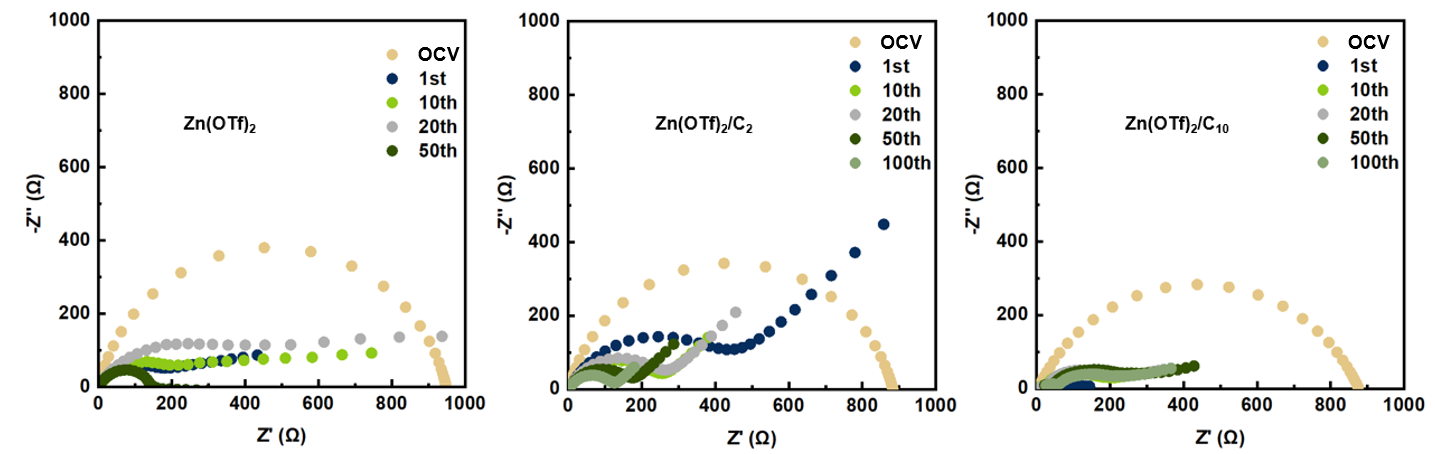


**Fig. S42** *In-situ* EIS spectra of Zn||Zn in different electrolytes


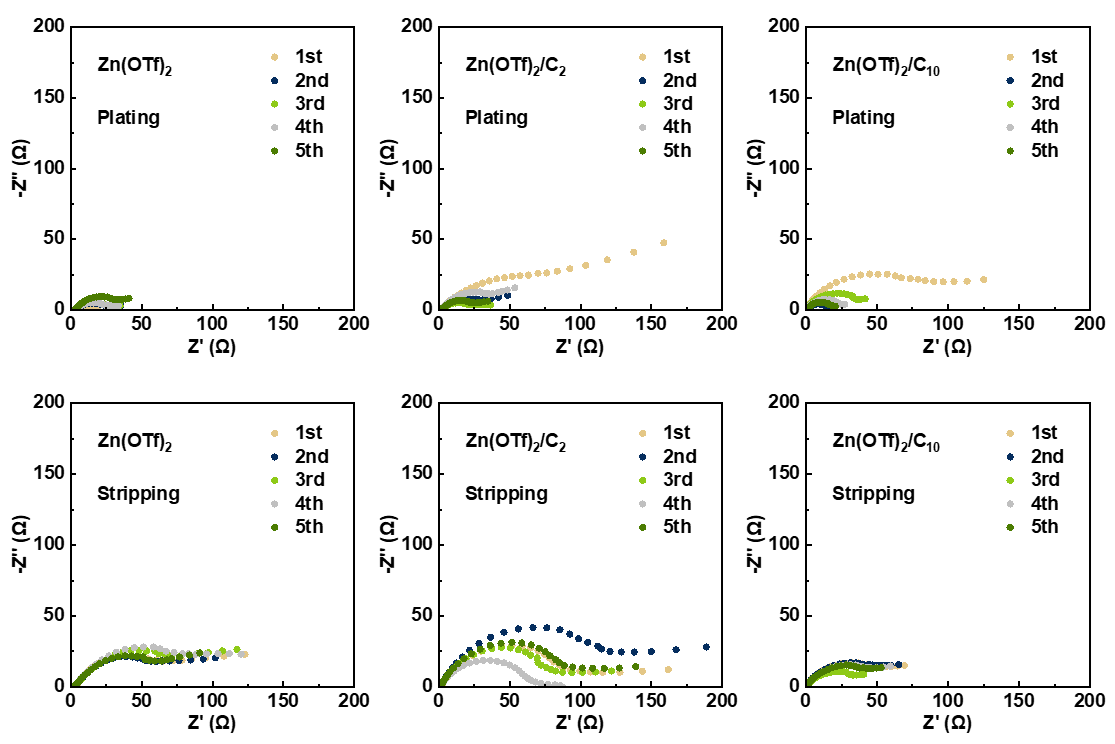


**Fig. S43** *In-situ* EIS results of the symmetric cells at the start/end of plating/stripping for the first five cycles

During the first plating process, both C_2_ and C_10_ exhibited a slight increase in *R_ct_* along with a pronounced low-frequency diffusion feature, indicating that these additives promote the formation of an ordered interfacial structure during the nucleation/growth stage. After interfacial activation, however, a substantial decrease in *R_ct_* was observed. Notably, the *R_ct_* of C_10_ dropped below that of the blank electrolyte, demonstrating its superior capability to establish a more favorable interfacial environment for Zn^2+^ deposition. More importantly, the stripping behavior shows a clear divergence between the additives. After stripping, the *R_ct_* of C_2_ remains comparable to or slightly higher than that of the blank electrolyte, indicating only limited improvements in reversibility and suppression of dead Zn. In contrast, C_10_ yields a markedly lower *R_ct_* after stripping, demonstrating that it effectively preserves interfacial contact during Zn removal. This advantage can be attributed to its self-assembly at the electrode surface, which promotes uniform deposition morphology, minimizes dead Zn formation, and stabilizes a conductive, low-resistance interphase in the LHCE system. Together, these results highlight that C_10_ not only facilitates Zn^2+^ deposition but also greatly enhances the reversibility of Zn^2+^ stripping.


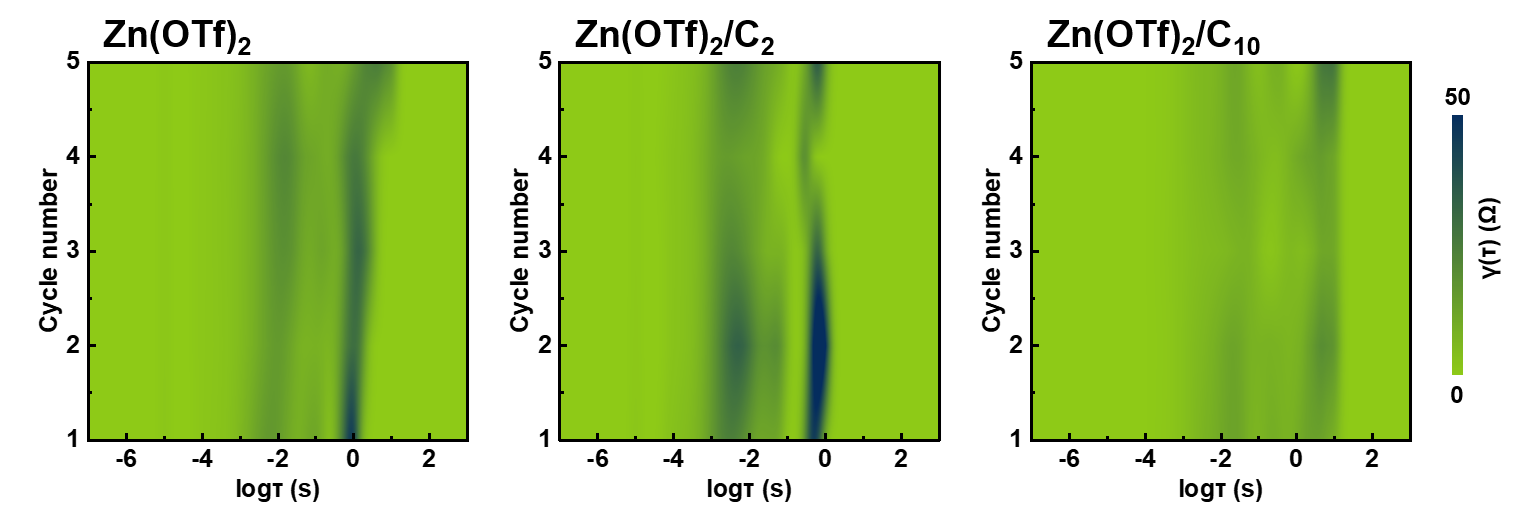


**Fig. S44** *In-situ* DRT results of the symmetric cells for the first five cycles

For C_10_, both the charge-transfer resistance (*R_ct_*) and diffusion-related impedance are significantly reduced, reflecting a highly conductive and uniform interface. The diffusion relaxation peak shifts to longer time scales (log *τ* ≈ 0–1 s), indicating that Zn^2+^ transport occurs through a more ordered and stabilized interfacial layer. This behavior can be attributed to the self-assembled ion channels formed by C_10_ at the electrode surface, as well as the favorable formation of a stable, uniform, and highly ion-conductive SEI in the LHCE environment. Together, these effects facilitate smooth ion migration while maintaining interfacial integrity, promoting uniform Zn deposition and reversible stripping.

In contrast, C_2_ exhibits increased *R_ct_* and enhanced diffusion impedance, with the diffusion peak remaining near log *τ* ≈ 0 s. This suggests that C_2_ lacks self-assembly and the advantageous LHCE properties, resulting in a less organized interface and hindered Zn^2+^ transport. Therefore, these results are fully consistent with the theoretical framework proposed in our manuscript.


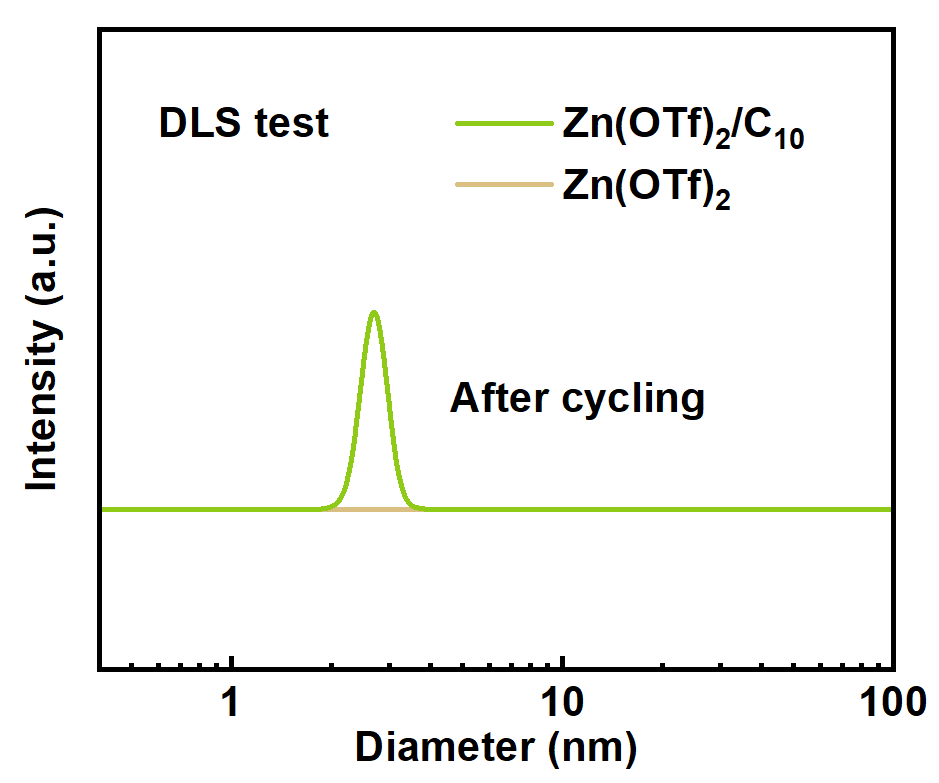


**Fig. S45** DLS spectra of different electrolytes after cycling


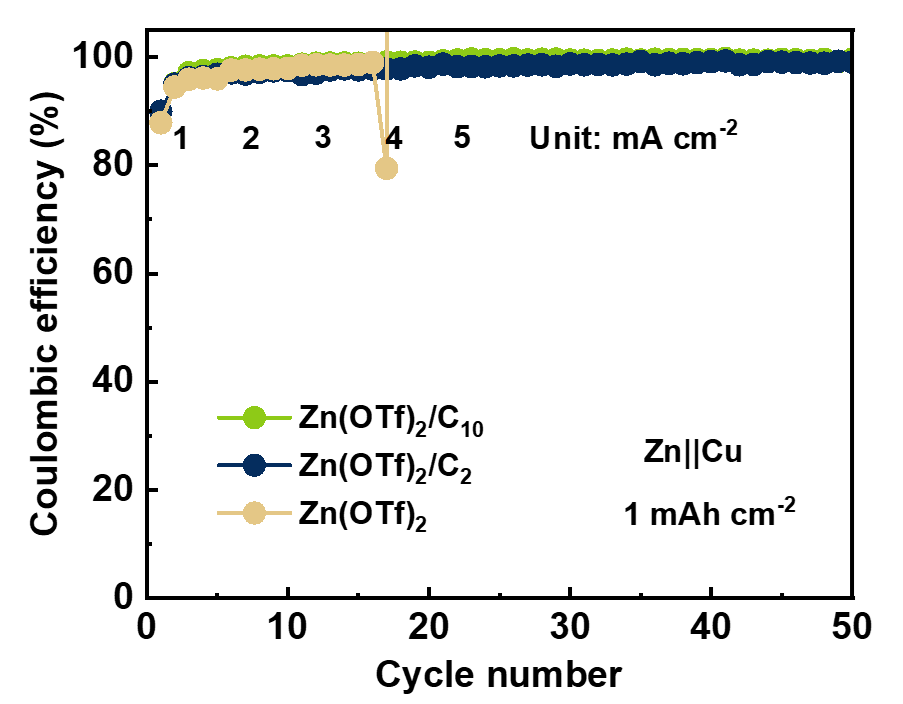


**Fig. S46** CEs of Zn||Cu asymmetric cells in different electrolytes


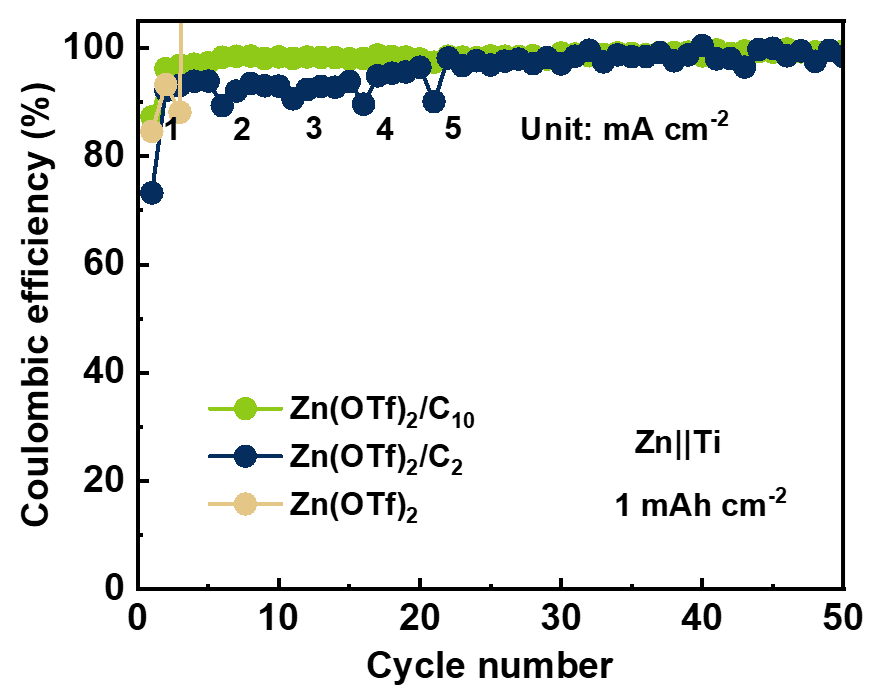


**Fig. S47** CEs of Zn||Ti asymmetric cells in different electrolytes


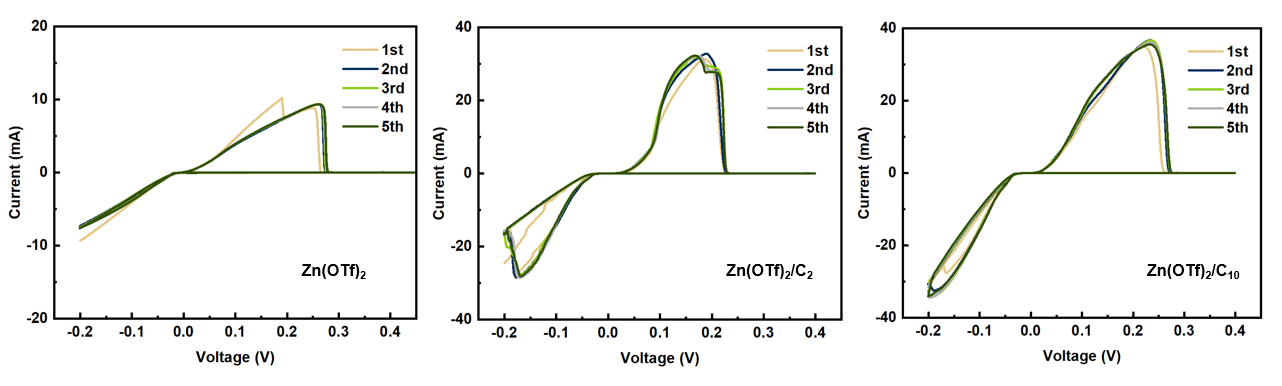


**Fig. S48** CV curves of Zn||Cu in different electrolytes


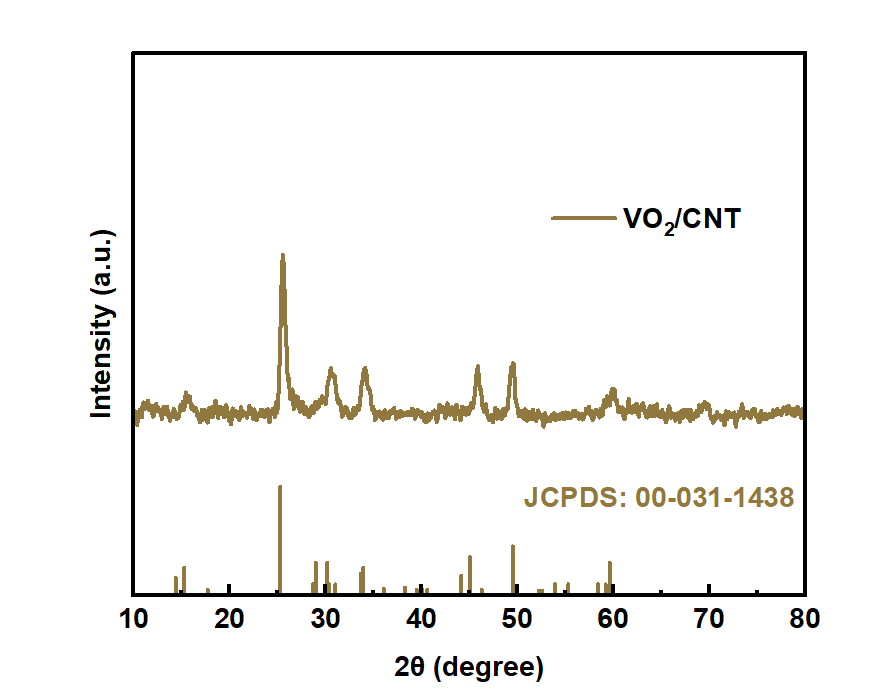


**Fig. S49** XRD spectra of VO_2_/CNT


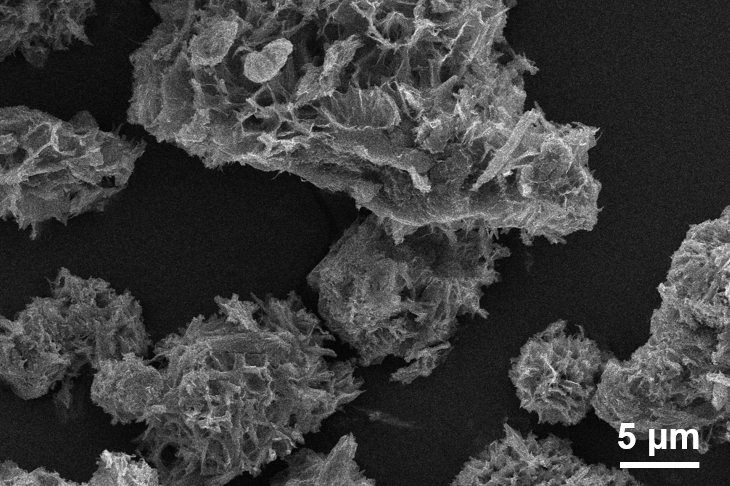


**Fig. S50** SEM image of VO_2_/CNT


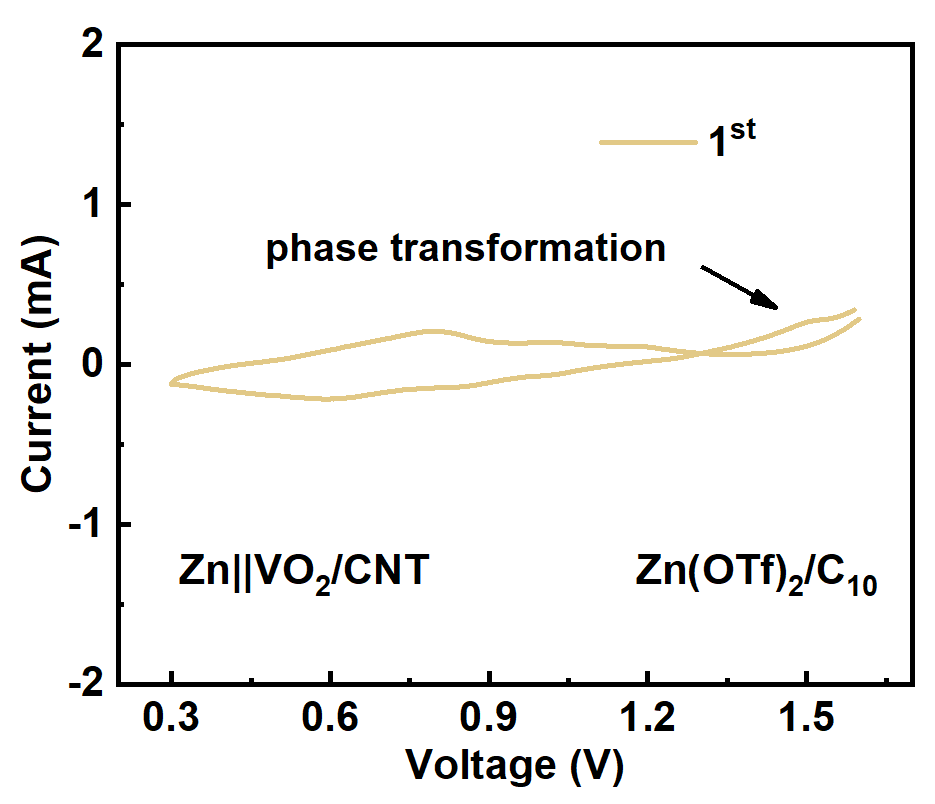


**Fig. S51** The 1^st^ CV curve of Zn||VO_2_/CNT using Zn(OTf)_2_/C_10_


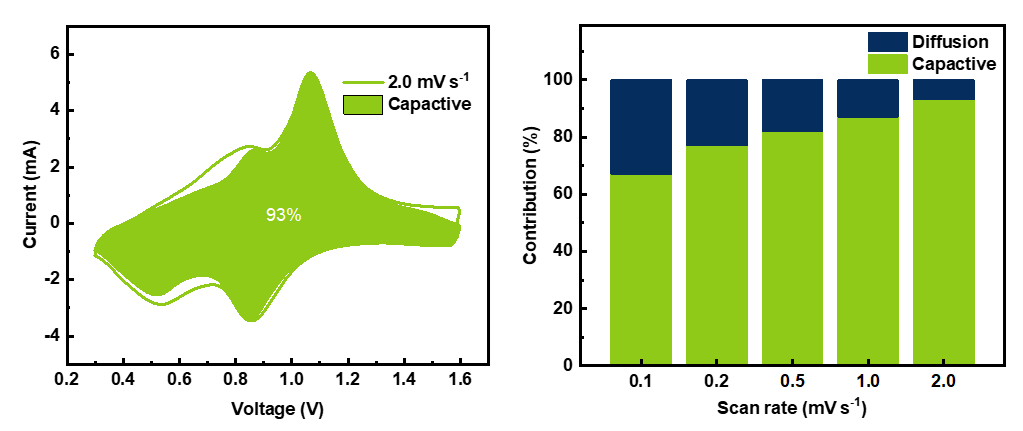


**Fig. S52** Capacitive contribution of Zn||VO_2_/CNT using Zn(OTf)_2_/C_10_


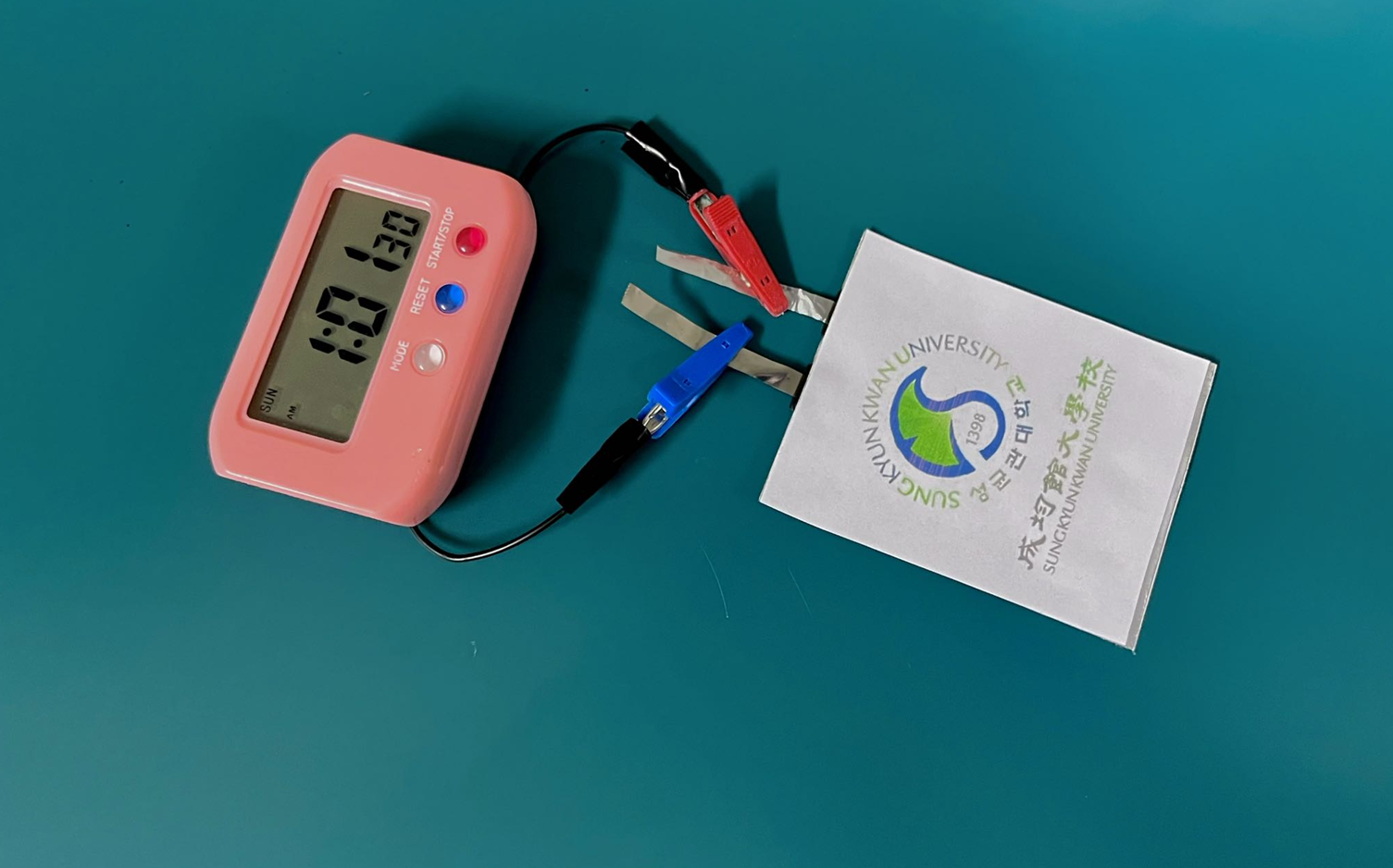


**Fig. S53** Practical application of Zn||VO_2_/CNT pouch cell with Zn(OTf)_2_/C_10_ electrolyte in powering an electronic alarm clock


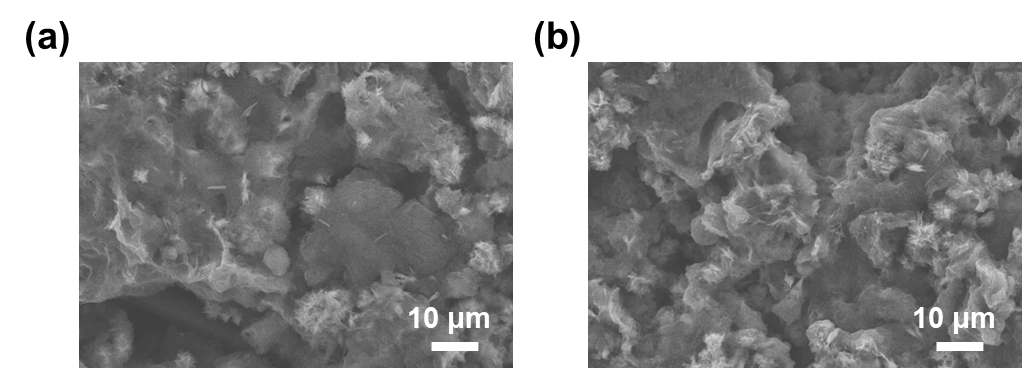


**Fig. S54** SEM image of VO_2_/CNT cathodes with Zn(OTf)_2_ after cycling


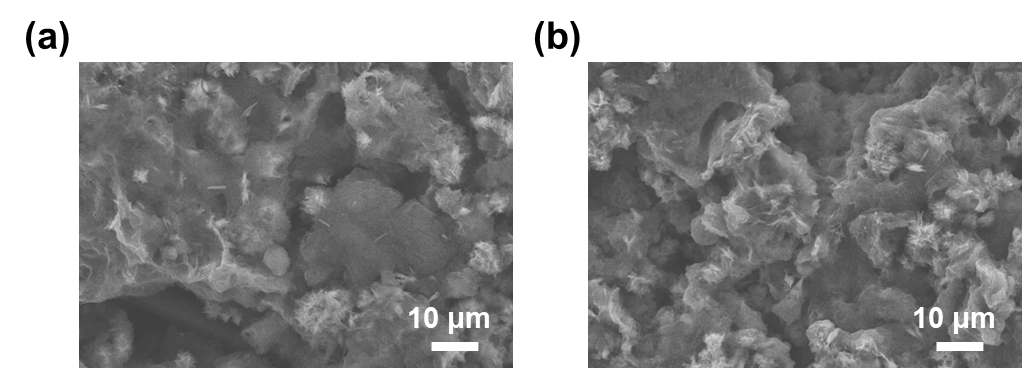


**Fig. S55** SEM image of VO_2_/CNT cathodes with Zn(OTf)_2_/C_10_ after cycling


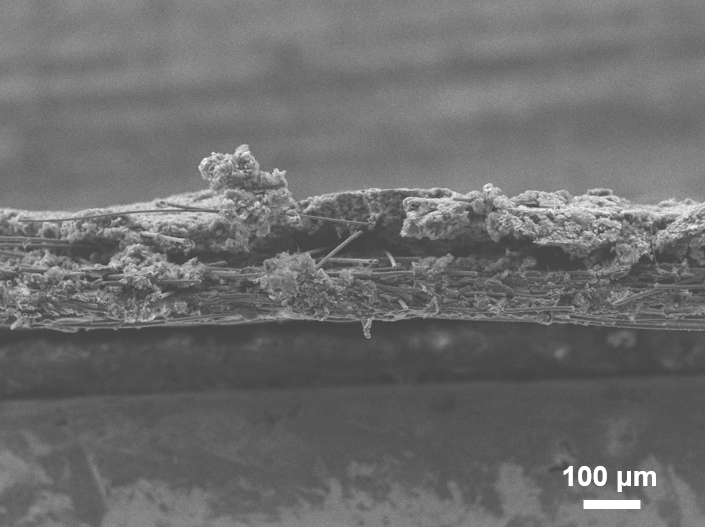


**Fig. S56** Cross-section SEM image of cathodes with Zn(OTf)_2_ after cycling


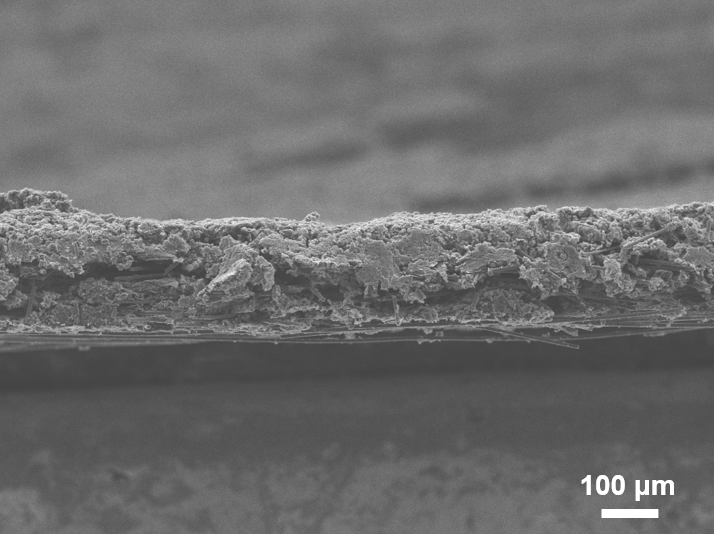


**Fig. S57** Cross-section SEM image of cathodes with Zn(OTf)_2_/C_10_ after cycling


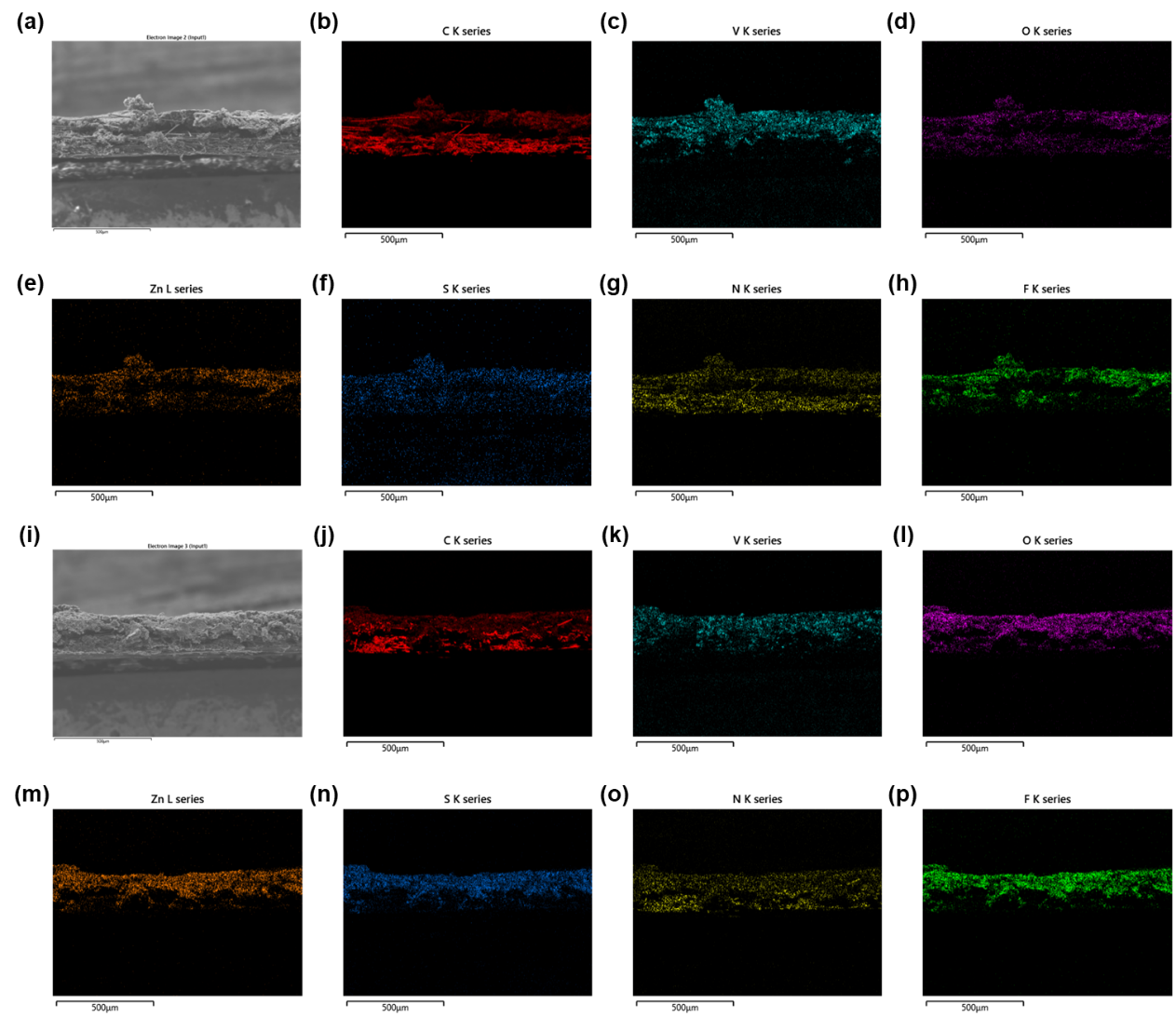


**Fig. S58** Corresponding EDX mapping images of cross-section SEM images of cathodes with (a-h) Zn(OTf)_2_, and (i-p) Zn(OTf)_2_/C_10_ after cycling


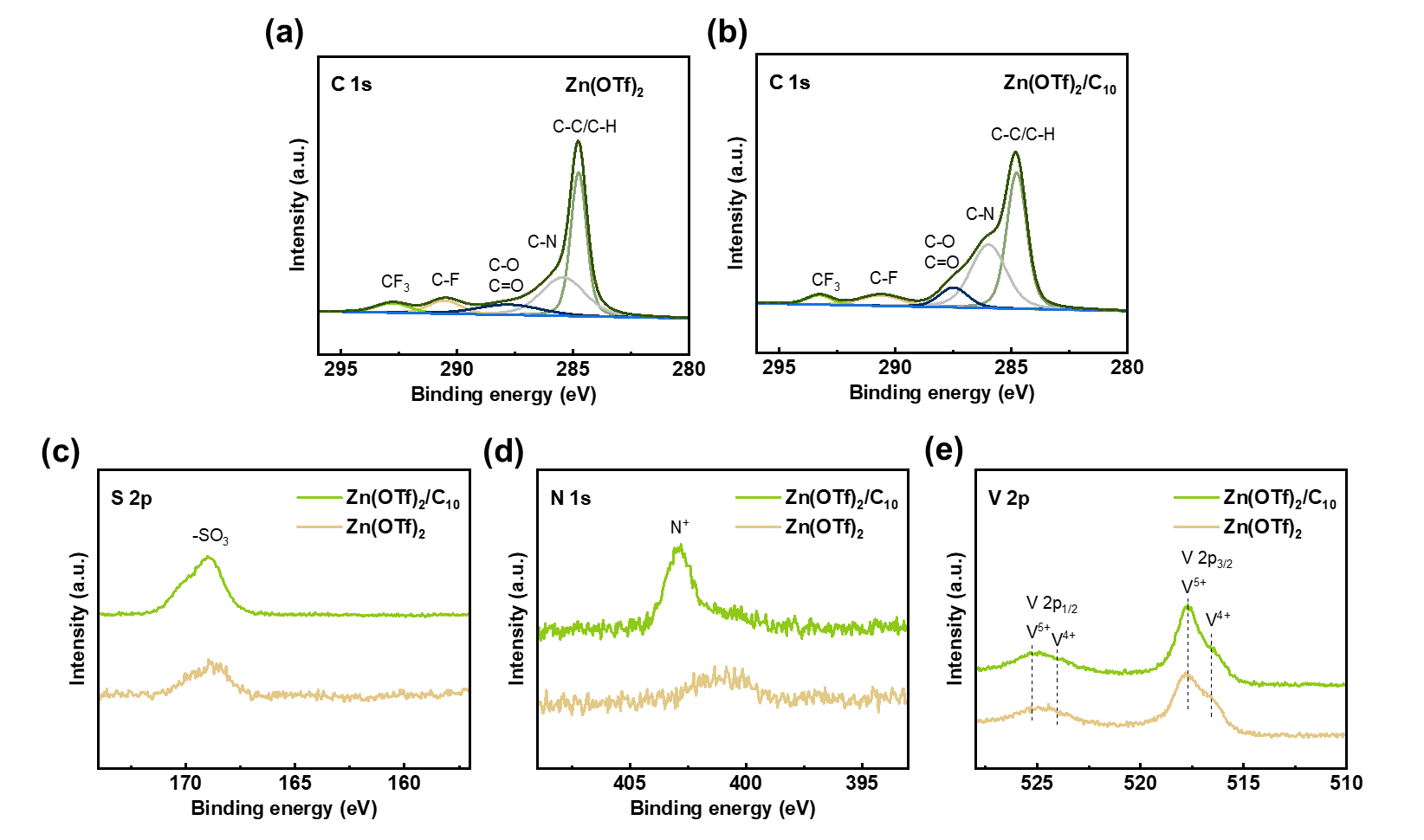


**Fig. S59** XPS C 1s spectra of cathodes with (**a**) Zn(OTf)_2_, and (**b**) Zn(OTf)_2_/C_10_ after cycling. (**c-e**) XPS S 2p, N 1s, and V 2p spectra of cathodes with different electrolytes after cycling

**Table S1** Key parameters related to SAXS of different electrolytes

| **Electrolyte** | ***R_g_* (nm) in Guinier plots** | **r_max_ (nm) in PDDF plots** |
| --- | --- | --- |
| Zn(OTf)_2_ | 9.902 | 26.540 |
| Zn(OTf)_2_/C_2_ | 10.051 | 24.630 |
| Zn(OTf)_2_/C_10_ | 23.541 | 54.813 |

**Table S2** Comparison of the relative contributions of different species

|  | CF_3_ | C-F | C-O | C-N | C-C/C-H |
| --- | --- | --- | --- | --- | --- |
| Zn(OTf)_2_ | 10.88% | 19.37% | 28.63% | 0% | 41.12% |
| Zn(OTf)_2_/C_2_ | 5.44% | 14.78% | 32.04% | 13.76% | 33.98% |
| Zn(OTf)_2_/C_10_ | 4.99% | 4.75% | 5.54% | 34.75% | 49.97% |

From the results in the table, it can be observed that the adsorption of the zwitterion introduces a distinct C–N peak at the Zn interface. In particular, C_10_ exhibits a higher proportion of C–N due to its self-assembly effect and stronger binding affinity with Zn. In contrast, in the blank electrolyte, where no zwitterion adsorption occurs, the interfacial signal is dominated by peaks associated with OTf⁻ species. This indirectly highlights the fundamentally different interfacial chemistries induced by zwitterions compared with those in conventional diluent-based systems.

**Table S3** Comparison of the recently reported cycling performance of Zn||Zn cells under 5 mA cm^-2^ and 5 mAh cm^-2^

| **No.** | **Strategy** | **Thickness of Zn (μm)** | **DOD (%)** | **Time (h)** | **Refs.** |
| --- | --- | --- | --- | --- | --- |
| 1 | HA polymer additive | 100 | 8.5 | 800 | [S23] |
| 2 | Nb_2_O_5_ coating | 100 | 8.5 | 220 | [S24] |
| 3 | Serine additive | 50 | 17.1 | 800 | [S25] |
| 4 | β-CD additive | 100 | 8.5 | 350 | [S26] |
| 5 | UIO-66-SO_3_H coating | 50 | 17.1 | 700 | [S27] |
| 6 | PSN coating | 30 | 28.5 | 400 | [S28] |
| 7 | Veratraldehyde additive | / | / | 800 | [S29] |
| 8 | Glucose additive | 100 | 8.5 | 270 | [S30] |
| 9 | Zn-Sn alloy anode | 24 | 35.6 | 240 | [S31] |
| **★** | **C_10_ co-solute** | **20** | **42.7** | **1240** | **This work** |

**Table S4** Comparison of cycling performances with recently reported high-loading V-based ZMBs

| **Strategy** | **Areal current density**  **(mA cm^-2^)** | **Mass loading**  **(mg cm^-2^)** | **Areal capacity**  **(mAh cm^-2^)** | **Refs.** |
| --- | --- | --- | --- | --- |
| DMAC+TMP  co-solvent | 3.3 | 6.6 | 0.8 | [S32] |
| BE+SIA | 0.46 | 9.2 | 1.8 | [S33] |
| 3D printed graphene | 4 | 11.4 | 2 | [S34] |
| PACMO/PEO coating | 17 | 17 | 2.33 | [S35] |
| N, S co-doped CDs | 11.52 | 11.52 | 2 | [S36] |
| Mn_2.5_V_10_O_24_∙5.9H_2_O | 6.15 | 12.3 | 3 | [S37] |
| H-SU additive | 12 | 60 | 4.2 | [S38] |
| **C_10_ co-solute** | **10** | **20** | **3.28** | **This work** |
|  | **5** | **50** | **8.10** |  |

**Table S5** Comparison of cycling performances with recently reported high-loading ZMBs except V-based

| **Cathode** | **Areal current density**  **(mA cm^-2^)** | **Mass loading**  **(mg cm^-2^)** | **Areal capacity**  **(mAh cm^-2^)** | **Refs.** |
| --- | --- | --- | --- | --- |
| 3DP MnO_2_ | 0.1 | 28.4 | 8.04 | [S39] |
| MNSMO@CC | ~0.7 | ~7 | 0.64 | [S40] |
| PANI | 1.578 | 15.78 | 1.25 | [S41] |
| cCNF/AC@I_2_ | 28.2 | 14.1 | 2.6 | [S42] |
| NMO/VTCNTs | 1 | 5 | 1.65 | [S43] |
| **MnVO** | **10** | **20** | **3.28** | **This work** |
|  | **5** | **50** | **8.10** |  |

**Table S6** Comparison of representative zwitterions/micelles works in ZMBs

|  | Materials  Types | Full cells  Performances |
| --- | --- | --- |
| **Our work** | C_10_  Zwitterion | Zn\|\|VO_2_/CNT  **8.10 mAh cm^−2^** after 150 cycles |
| Energy Environ. Sci. **16**, 2684-2695 (2023) | L-carnitine  Zwitterion | Zn\|\|V_2_O_5_  ~0.12 mAh cm^-2^ after 3500 cycles |
| Nano-Micro Lett. **17**, 314 (2025) | MPC  Zwitterion | Zn\|\|V_2_O_5_  1 mAh cm^-2^ after 300 cycles |
| ACS Nano **19**, 32943-32955  (2025) | BES  Zwitterion | Zn\|\|ZnVO  ~0.4 mAh cm^-2^ after 2700 cycles |
| Adv. Funct. Mat. **34**, 2401889 (2024) | ggg  Zwitterion | Zn\|\|MnO_2_  ~0.12 mAh cm^-2^ after 900 cycles |
| Energy Storage Mater. **64**, 103059 (2024) | TMB  Zwitterion | Zn\|\|V_2_O_5_  ~0.5 mAh cm^-2^ after 1000 cycles |
| Nano Energy **142**, 111178 (2025) | NDSB  Zwitterion | Zn\|\|AlVO  ~0.27 mAh cm^-2^ after 1000 cycles |
| Adv. Funct. Mat. **35**, 2417695 (2024) | POPSO-Na  Micelle | Only ZIC |
| Chem. Eng. J. **503**, 158436 (2025) | SDBS  Micelle | Zn\|\|NH_4_VO  ~0.3 mAh cm^-2^ after 500 cycles |
| Adv. Energy Mat. **12**, 2103557 (2022) | TMBAC  Micelle | Zn\|\|VO_2_  ~0.7 mAh cm^-2^ after 100 cycles |

To clarify and highlight the key innovations of our study, we summarize them as follows:

**1. First demonstration of LHCEs formed by zwitterions**

Although several studies have previously reported the use of zwitterions, our work is the first to reveal that zwitterions can induce the formation of localized high-concentration electrolytes (LHCEs), which fundamentally differs from their conventional roles. Importantly, this is the first demonstration of LHCEs induced by zwitterions, which is fundamentally different from the conventional diluent-induced LHCEs.

Importantly, the formation of LHCEs induced by the zwitterionic C_10_ enables the formation of a stable and robust hybrid ZnS/ZnF_2_ SEI, which in turn allows us to demonstrate practical full cell performance (8.10 mAh cm⁻^2^ after 150 cycles with a cathode mass loading of 50 mg cm⁻^2^).

**2. First** **rigorous evidence of aggregate formation by zwitterions**

To the best of our knowledge, there have been virtually no prior reports demonstrating that zwitterions can self-assemble into aggregates. In earlier studies involving micelle-type additives, the authors typically relied on schematic illustrations after observing aggregation, and merely suggested a spherical micelle structure without providing scientific proof. Such an approach is not sufficiently rigorous.

**3. Comprehensive structural verification of spherical aggregates**

For the first time, we introduced Guinier, pair distance distribution function (PDDF), and Porod analyses into the field of Zn metal batteries electrolytes, and, in conjunction with complementary spectroscopic characterizations and DFT/MD, we conclusively validated the presence of spherical aggregates.

**Table S7** Comparison of representative diluent-based works in ZMBs and our work

|  | Materials  Type | Symmetric cells  Performances | Full cells  Performances |
| --- | --- | --- | --- |
| **Our work** | C_10_  Zwitterion | **5 mA cm^−2^, 5 mAh cm^−2^**  **> 1200 h (DOD = 42.7%)** | Zn\|\|VO_2_/CNT (**50 mg cm^-2^**)  **8.10 mAh cm^−2^** after 150 cycles |
| Angew. Chem. Int. Ed. Early View, e202511410 (2025) | TPP  Diluent | 5 mA cm^−2^, 5 mAh cm^−2^  > 1150 h (DOD = 17.1%) | Zn\|\|NaVO (2-5 mg cm^-2^)  >0.5 mAh cm^−2^ after 2000 cycles |
| J. Mater. Chem. A **9**, 22347-22352 (2021) | 1,4-dioxane  Diluent | 0.5 mA cm^−2^, 0.5 mAh cm^−2^  400 h (DOD = 4.3%) | Zn\|\|V_2_O_5_ (2±0.5 mg cm^-2^)  >0.09 mAh cm^−2^ after 100 cycles |
| Angew. Chem. Int. Ed. **64**, e202501183 (2025) | 1,4-dioxane  /emim+  Diluent | 1.17 mA cm^−2^, 1.17 mAh cm^−2^  1200 h (DOD = 20%) | Zn\|\|MnVO (10 mg cm^-2^)  3.3 mAh cm^−2^ after 300 cycles |
| Adv. Funct. Mat. Early View, e15837 (2025) | APT  Diluent | 2 mA cm^−2^, 2 mAh cm^−2^  1300 h (DOD = 3.4%) | Zn\|\|NVO (2 mg cm^-2^)  ~0.6 mAh cm^−2^ after 3300 cycles |
| Angew. Chem. Int. Ed. **62**, e202315834 (2023) | THF  Diluent | 0.25 mA cm^−2^, 0.25 mAh cm^−2^  2000 h (no thickness data) | Zn\|\|PANI (1 mg cm^-2^)  0.1 mAh cm^−2^ after 500 cycles |

In conventional diluent-type LHCEs, the localized solvation structure mainly arises from the dilution effect, where non-coordinating solvents weaken the Zn^2+^–H_2_O interaction and promote the formation of anion-rich solvation shells. However, such systems typically require a large fraction of inert diluents, which inevitably reduces ionic conductivity and limits practical applicability.

In contrast, our C_10_-based system achieves localized high concentration through self-assembled zwitterionic aggregates, which promote the formation of CIP/AGG via zwitterionic effects. This design does not rely on diluents, thereby avoiding the trade-off between solvation modulation and conductivity. Moreover, the amphiphilic nature of C_10_ further induces vertical self-assembly at the interface, forming ordered ionic channels that enable pre-accumulation of Zn^2+^ near the electrode surface and subsequent uniform deposition—an interfacial regulation mechanism absent in conventional LHCEs.

These mechanistic distinctions collectively demonstrate that our approach not only reproduces the solvation advantages of diluent-type LHCEs, achieving more uniform and stable SEI formation, but also adds a unique self-assembly–driven interfacial regulation, broadening the conceptual framework for LHCE design and enhancing practical electrochemical performance.

**Table S8** EDX elemental analysis results of Zn(OTf)_2_, and Zn(OTf)_2_/C_10_

| Zn(OTf)_2_/ Zn(OTf)_2_/C_10_ | Line | wt% | wt% sigma | atomic% |
| --- | --- | --- | --- | --- |
| C | K series | 66.54/58.13 | 0.45/0.31 | 79.55/71.90 |
| N | K series | 2.77/3.38 | 0.59/0.45 | 2.84/3.59 |
| O | K series | 10.41/13.78 | 0.22/0.18 | 9.35/12.80 |
| F | K series | 5.46/8.69 | 0.10/0.10 | 4.13/6.79 |
| S | K series | 0.39/3.05 | 0.02/0.03 | 0.17/1.41 |
| V | K series | 12.83/8.79 | 0.12/0.08 | 3.62/2.56 |
| Zn | L series | 1.60/4.17 | 0.05/0.06 | 0.35/0.95 |

**Supplementary References**

[S1] M. J. Frisch, G. W. Trucks, H. B. Schlegel, et al. Gaussian 09 Revision E.01, Gaussian Inc., Wallingford CT, 2009.

[S2] P.J. Stephens, F.J. Devlin, C.F. Chabalowski, M.J. Frisch, Ab initio calculation of vibrational absorption and circular dichroism spectra using density functional force fields. J. Phys. Chem. **98**(45), 11623–11627 (1994). https://doi.org/10.1021/j100096a001

[S3] A.D. Becke, Density-functional thermochemistry. III. The role of exact exchange. J. Chem. Phys. **98**(7), 5648–5652 (1993). https://doi.org/10.1063/1.464913

[S4] R. Krishnan, J.S. Binkley, R. Seeger, J.A. Pople, Self-consistent molecular orbital methods. XX. A basis set for correlated wave functions. J. Chem. Phys. **72**(1), 650–654 (1980). https://doi.org/10.1063/1.438955

[S5] A. Schäfer, H. Horn, R. Ahlrichs, Fully optimized contracted Gaussian basis sets for atoms Li to Kr. J. Chem. Phys. **97**(4), 2571–2577 (1992). https://doi.org/10.1063/1.463096

[S6] A. Schäfer, C. Huber, R. Ahlrichs, Fully optimized contracted Gaussian basis sets of triple *Zeta* Valence quality for atoms Li to Kr. J. Chem. Phys. **100**(8), 5829–5835 (1994). https://doi.org/10.1063/1.467146

[S7] S. Grimme, J. Antony, S. Ehrlich, H. Krieg, A consistent and accurate *ab initio* parametrization of density functional dispersion correction (DFT-D) for the 94 elements H-Pu. J. Chem. Phys. **132**(15), 154104 (2010). https://doi.org/10.1063/1.3382344

[S8] S. Grimme, S. Ehrlich, L. Goerigk, Effect of the damping function in dispersion corrected density functional theory. J. Comput. Chem. **32**(7), 1456–1465 (2011). https://doi.org/10.1002/jcc.21759

[S9] B.P. Pritchard, D. Altarawy, B. Didier, T.D. Gibson, T.L. Windus, New basis set exchange: an open, up-to-date resource for the molecular sciences community. J. Chem. Inf. Model. **59**(11), 4814–4820 (2019). https://doi.org/10.1021/acs.jcim.9b00725

[S10] A.V. Marenich, C.J. Cramer, D.G. Truhlar, Universal solvation model based on solute electron density and on a continuum model of the solvent defined by the bulk dielectric constant and atomic surface tensions. J. Phys. Chem. B **113**(18), 6378–6396 (2009). https://doi.org/10.1021/jp810292n

[S11] T. Lu, F. Chen, Multiwfn: a multifunctional wavefunction analyzer. J. Comput. Chem. **33**(5), 580–592 (2012). https://doi.org/10.1002/jcc.22885

[S12] T. Lu. A comprehensive electron wavefunction analysis toolbox for chemists, Multiwfn. J. Chem. Phys. **161**, 082503 (2024). https://doi.org/10.1063/5.0216272

[S13] G. Kresse, J. Furthmüller, Efficient iterative schemes for *ab initio* total-energy calculations using a plane-wave basis set. Phys. Rev. B **54**(16), 11169–11186 (1996). https://doi.org/10.1103/physrevb.54.11169

[S14] J.P. Perdew, K. Burke, M. Ernzerhof, Generalized gradient approximation made simple. Phys. Rev. Lett. **77**(18), 3865–3868 (1996). https://doi.org/10.1103/physrevlett.77.3865

[S15] P.E. Blöchl, Projector augmented-wave method. Phys. Rev. B **50**(24), 17953–17979 (1994). https://doi.org/10.1103/physrevb.50.17953

[S16] S. Plimpton, Fast parallel algorithms for short-range molecular dynamics. J. Comput. Phys. **117**(1), 1–19 (1995). https://doi.org/10.1006/jcph.1995.1039

[S17] W.L. Jorgensen, D.S. Maxwell, J. Tirado-Rives, Development and testing of the OPLS all-atom force field on conformational energetics and properties of organic liquids. J. Am. Chem. Soc. **118**(45), 11225–11236 (1996). https://doi.org/10.1021/ja9621760

[S18] G.A. Kaminski, R.A. Friesner, J.T.-Rives, W.L. Jorgensen, Evaluation and Parameterization of a Generalized Amber Force Field. J. Phys. Chem. B **105**, 6474–6487 (2001). https://doi.org/10.1021/jp003919d

[S19] L. Martínez, R. Andrade, E.G. Birgin, J.M. Martínez, PACKMOL: a package for building initial configurations for molecular dynamics simulations. J. Comput. Chem. **30**(13), 2157–2164 (2009). https://doi.org/10.1002/jcc.21224

[S20] J.M. Martínez, L. Martínez, Packing optimization for automated generation of complex system's initial configurations for molecular dynamics and docking. J. Comput. Chem. **24**(7), 819–825 (2003). https://doi.org/10.1002/jcc.10216

[S21] W. Humphrey, A. Dalke, K. Schulten, VMD: Visual molecular dynamics. J. Mol. Graph. **14**(1), 33–38 (1996). https://doi.org/10.1016/0263-7855(96)00018-5

[S22] S. Nosé, A unified formulation of the constant temperature molecular dynamics methods. J. Chem. Phys. **81**(1), 511–519 (1984). https://doi.org/10.1063/1.447334

# [S23] M.J. Qiu, P. Sun, W.J. Mai, G.F. Cui, Chaotropic polymer additive with ion transport tunnel enable dendrite-free zinc battery. ACS Appl. Mater. Interfaces 14, 40951–40958 (2022). https://doi.org/10.1021/acsami.2c10517

[S24] S.J. So, Y.N. Ahn, J.W. Ko, I. Kim, J. Hur, Uniform and oriented zinc deposition induced by artificial Nb_2_O_5_ Layer for highly reversible Zn anode in aqueous zinc ion batteries. Energy Storage Mater. **52**, 40–51 (2022). https://doi.org/10.1016/j.ensm.2022.07.036

[S25] Y.F. Wang, L. Mo, X.X. Zhang, Y.K. Ren, T.T. Wei et al., Facet-termination promoted uniform Zn (100) deposition for high-stable zinc-ion batteries. Adv. Energy Mater. **13**, 2202875 (2023). https://doi.org/10.1002/aenm.202301517

[S26] M.J. Qiu, P. Sun, Y. Wang, L. Ma, C.Y. Zhi et al., Inside cover: anion-trap engineering toward remarkable crystallographic reorientation and efficient cation migration of Zn ion batteries. Angew. Chem. Int. Ed. **61**, e202206015 (2022). https://doi.org/10.1002/anie.202214654

[S27] H. F. Fan, M. Li, E. D. Wang, Anion-functionalized interfacial layer for stable Zn metal anodes. Nano Energy **103**, 107751 (2022). https://doi.org/10.1016/j.nanoen.2022.107751

[S28] S. Zhou, Y.P. Wang, H.T. Lu, Y.F. Zhang, C.Y. Fu et al., Anti-corrosive and zn-ion-regulating composite interlayer enabling long-life Zn metal anodes. Adv. Funct. Mater. **31**, 2101234 (2021). https://doi.org/10.1002/adfm.202104361

[S29] M.J. Qiu, L. Ma, P. Sun, Z.L. Wang, G.F. Cui, W.J. Mai, Manipulating interfacial stability via absorption-competition mechanism for long-lifespan Zn anode. Nano-Micro Lett. **14**, 103 (2022). https://doi.org/10.1007/s40820-021-00777-2

[S30] P. Sun, L. Ma, W.H. Zhou, M.J. Qiu, Z.L. Wang et al., Simultaneous regulation on solvation shell and electrode interface for dendrite-free zn ion batteries achieved by a low-cost glucose additive. Angew. Chem. Int. Ed. **60**, 18247–18255 (2021). https://doi.org/10.1002/anie.202105756

[S31] L.Y. Wang, W.W. Huang, W.B. Guo, Z.H. Guo, C.Y. Chang et al., Sn Alloying to inhibit hydrogen evolution of Zn metal anode in rechargeable aqueous batteries. Adv. Funct. Mater. **32**, 2104567 (2022). https://doi.org/10.1002/adfm.202108533

[S32] Y.Y. Wang, Z.J. Wang, W.K. Pang, W. Lie, J.A. Yuwono et al., Solvent control of water O−H bonds for highly reversible zinc ion batteries. Nat. Commun. **14**, 1234 (2023). https://doi.org/10.1038/s41467-023-38384-x

[S33] K.Q. Feng, B.C. Chen, B.J. Xi, C.X. Tian, B.Y. Sang et al., Antioxidant interfaces enabled by self-deoxidizing and self-dehydrogenating redox couple for reversible zinc metal batteries. Adv. Energy Mater. **14**, 2300678 (2024). https://doi.org/10.1002/aenm.202401053

[S34] B.K. Wu, B.B. Guo, Y.Z. Chen, Y.B. Mu, H.Q. Qu et al., High zinc utilization aqueous zinc ion batteries enabled by 3d printed graphene arrays. Energy Storage Mater. **54**, 75–84 (2023). https://doi.org/10.1016/j.ensm.2022.10.017

[S35] J. Feng, X.Y. Li, Y.X. Ouyang, H.Y. Zhao, N. Li et al., Regulating Zn^2+^ migration-diffusion behavior by spontaneous cascade optimization strategy for long-life and low N/P ratio zinc ion batteries. Angew. Chem. Int. Ed. **63**, e202310015 (2024). https://doi.org/10.1002/anie.202407194

[S36] Z. Xu, H. Li, Y.P. Liu, K.X. Wang, H.B. Wang et al., Durable modulation of Zn(002) plane deposition via reproducible zincophilic carbon quantum dots towards low N/P ratio zinc-ion batteries. Mater. Horiz. **10**, 3680–3693 (2023). https://doi.org/10.1039/D3MH00261F

[S37] L. Yang, Y.J. Zhu, F.L. Zeng, L.Y. Dong, J.C. Tao et al., Synchronously promoting the electron and ion transport in high-loading Mn_2.5_V_10_O_24∙5_.9H_2_O cathodes for practical aqueous zinc-ion batteries. Energy Storage Mater. **65**, 103162 (2024). https://doi.org/10.1016/j.fueleneab.2023.12.004

[S38] S.Y. Huang, P. Zhang, J. Lu, J.S. Kim, D.H. Min et al., Molecularly engineered multifunctional imide derivatives for practical Zn metal full cells. Energy Environ. Sci. **17**, 7870–7881 (2024). https://doi.org/10.1039/D4EE02867H

[S39] H. Yang, Y. Wan, K. Sun, M.D. Zhang, C.Z. Wang et al., Reconciling Mass Loading and Gravimetric Performance of MnO_2_ Cathodes by 3D-Printed Carbon Structures for Zinc-Ion Batteries. Adv. Funct. Mater. **33**, 2401234 (2023). https://doi.org/10.1002/adfm.202215076

[S40] W.J. Zheng, Z.B. Cui, C. Liu, L.B. Yuan, S.S. Li et al., Tailoring hierarchical MnO_2_ nanostructures on self-supporting cathodes for high-mass-loading zinc-ion batteries. Chem. Sci. **15**, 20303–20314 (2024). https://doi.org/10.1039/D4SC06182A

[S41] R.F. Yu, Y.F. Xu, X.X. Liu, G.X. Zhang, H.P. Du et al., Screen-printed high loading flexible Zn-PANI battery. Chem. Eng. J. **497**, 154788 (2024). https://doi.org/10.1016/j.cej.2024.154788

[S42] Z. L. Li, W. W. Cao, T. Hu, Y. C. Hu, R. Zhang et al., Deploying cationic cellulose nanofiber confinement to enable high iodine loadings towards high energy and high-temperature Zn-I_2_ battery. Angew. Chem. Int. Ed. **136**, e202317652 (2024). https://doi.org/10.1002/anie.202482011

[S43] X. Gao, C. Y. Zhang, Y. H. Dai, S. Y. Zhao, X. Y. Hu et al., Three-dimensional manganese oxide@carbon networks as free-standing, high-loading cathodes for high-performance zinc-ion batteries. Small Struct. **4**, 2300456 (2023).  https://doi.org/10.1002/sstr.202200316
